# Targeting the lactylation of ENO1 alleviates endothelial dysfunction in sepsis

**DOI:** 10.1002/ctm2.70597

**Published:** 2026-01-14

**Authors:** Xueru Xie, Tingyan Liu, Caiyan Zhang, Ye Cheng, Yajing Gao, Wenfeng Xiao, Haiyan Guo, Yutong Zhou, Yawei Yu, Kexin Wang, Yinghong Lin, Lisheng Xiao, Yingying Zhang, Weiguo Yang, Gangfeng Yan, Guoping Lu, Yufeng Zhou

**Affiliations:** ^1^ NHC Key Laboratory of Neonatal Diseases Children's Hospital of Fudan University and the Shanghai Key Laboratory of Medical Epigenetics, International Co‐laboratory of Medical Epigenetics and Metabolism, Ministry of Science and Technology, Institutes of Biomedical Sciences, Fudan University Shanghai China; ^2^ Department of Critical Care Medicine and Department of Emergency Children's Hospital of Fudan University, National Children's Medical Center, Fudan University Shanghai China; ^3^ Fujian Key Laboratory of Neonatal Diseases Xiamen Key Laboratory of Neonatal Diseases Xiamen Children`s Hospital (Children's Hospital of Fudan University at Xiamen) Xiamen China; ^4^ Pediatric Intensive Care Unit Shenzhen Children's Hospital Shenzhen China; ^5^ School of Public Health & Shanghai Institute of Infectious Disease and Biosecurity Fudan University Shanghai China

**Keywords:** endothelial dysfunction, ENO1, lactylation, sepsis

## Abstract

**Background:**

Elevated lactate is associated with vascular endothelial dysfunction, a factor that can contribute to organ failure in sepsis. However, the specific mechanisms involved have yet to be fully elucidated. Here, we investigated the role of enolase 1 (ENO1) lactylation in modulating the functions of endothelial cells (ECs) in sepsis pathogenesis.

**Methods:**

The septic mouse model was established using two methods: cecal ligation and puncture (CLP) and intraperitoneal injection of LPS. AAV‐ENO1 shRNA was administered to ablate ENO1 in vascular endothelial cells of mice. Tail vein injection of .5% Evans Blue Dye (EBD) was utilised to assess microvascular permeability in septic mice. Post‐translational modification (PTM) mass spectrometry was employed to detect key proteins undergoing lactylation in endothelial cells. Additionally, CCK‐8 assay, Transwell assay, and scratch wound healing assay were performed to evaluate the fundamental functions of ECs. Further investigations were conducted through Western blotting, Co‐immunoprecipitation (CO‐IP), RT‐qPCR, RNA immunoprecipitation (RIP) and RNA sequencing to examine genes/proteins involved in vascular endothelial injury and their interactions.

**Results:**

We found that elevated lactate in sepsis promoted the lactylation of ENO1 at the K71 residue, facilitated by the increased activity of the lactyltransferase P300. This modification reduced the binding of *TRIM21* mRNA to ENO1, thereby preventing its degradation by limiting the recruitment of CNOT6. Consequently, the stability and expression of *TRIM21* mRNA were enhanced. Elevated TRIM21 subsequently binds to vascular endothelial‐cadherin (VE‐Cadherin), promoting its ubiquitination and degradation, disrupting endothelial adherens junctions (AJs) and increasing endothelial permeability. Targeting the lactylation of ENO1 at K71 with a specific inhibitory peptide alleviated endothelial injury and improved survival rates in septic mice.

**Conclusions:**

These findings suggest that ENO1 lactylation plays a pivotal role in vascular endothelial dysfunction during sepsis. Inhibiting lactylation may offer a therapeutic strategy for sepsis treatment.

## Graphical abstract

Lactylation of ENO1 reduced the binding of TRIM21 mRNA to ENO1, thereby preventing mRNA degradation. Elevated TRIM21 subsequently binds to VE‐Cadherin, promoting its ubiquitination. Targeting the lactylation of ENO1 with a specific inhibitory peptide alleviated endothelial dysfunction.

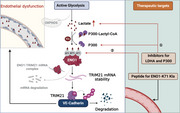

## 1 INTRODUCTION

Sepsis is a life‐threatening condition that is characterised by systemic organ dysfunction resulting from a dysregulated host response to infection (Sepsis‐3).^1^, ^2^, ^3^, ^4^ A defining pathological feature of sepsis is vascular endothelial dysfunction, which results in microvascular leakage, tissue edema, impaired perfusion, and ultimately multiple organ failure.^5^, ^6^, ^7^, ^8^ However, the molecular mechanisms responsible for endothelial barrier disruption in sepsis are still not fully understood.

Endothelial barrier integrity relies on AJs, with VE‐Cadherin serving as a central structural component.^9^, ^10^ Loss of VE‐Cadherin leads to junctional destabilisation and increased vascular permeability, a hallmark of septic endothelial injury.^8^, ^9^ Although inflammatory signals are known to trigger VE‐Cadherin internalisation and degradation,^6^ the upstream metabolic drivers and post‐transcriptional mechanisms that control VE‐Cadherin stability during sepsis remain unclear.

Lactate accumulation is a prominent metabolic feature of sepsis and is widely used as a prognostic biomarker in clinical practice.^1^, ^2^, ^11^, ^12^ Beyond reflecting metabolic stress, lactate has emerged as an active signalling molecule that regulates immune and EC functions.^13^, ^14^, ^15^, ^16^ In ECs, lactate has been shown to promote VE‐Cadherin degradation and increase vascular permeability.^17^ However, how lactate mechanistically drives endothelial barrier breakdown remains largely unresolved.

Lactylation, a recently identified PTM derived from lactate, provides a potential link between metabolic reprogramming and cellular dysfunction.^18^, ^19^, ^20^, ^21^ While initially described on histones, lactylation has since been detected on many non‐histone proteins and shown to regulate diverse biological processes.^18^, ^19^, ^20^, ^22^, ^23^ In the high‐lactate environment of sepsis, protein lactylation is markedly increased, yet the key endothelial targets and their functional relevance remain poorly defined.

ENO1 is a multifunctional protein best known for its role in glycolysis but also capable of binding RNA and regulating mRNA stability.^24^, ^25^, ^26^ Recent studies have highlighted the importance of ENO1's RNA‐binding function in controlling gene expression.^25^, ^27^ Whether ENO1 undergoes lactylation in sepsis and how such modification affects its RNA‐binding activity and endothelial barrier integrity have not been investigated.

In this study, we address the unresolved mechanism underlying VE‐Cadherin degradation in sepsis by defining a lactate‐driven regulatory pathway. We demonstrate that sepsis‐associated lactate accumulation induces P300‐mediated lactylation of ENO1 at lysine 71 in ECs. This modification weakens the binding of ENO1 to *TRIM21* mRNA, resulting in enhanced mRNA stability and increased TRIM21 protein expression. Elevated TRIM21 then promotes the ubiquitination and degradation of VE‐Cadherin, leading to AJs disruption and increased vascular permeability. Importantly, selective inhibition of ENO1 K71 lactylation restores endothelial barrier function and improves survival in septic mice.

Together, these findings identify ENO1 lactylation as a critical molecular link between metabolic dysregulation and endothelial barrier failure in sepsis. This work reveals a previously unrecognised post‐transcriptional mechanism controlling VE‐Cadherin stability and highlights ENO1 lactylation as a potential therapeutic target for vascular protection in sepsis.

## 2 RESULTS

### 2.1 Elevated lactate promoted endothelial lactylation and derived vascular barrier dysfunction in sepsis

**FIGURE 1:**
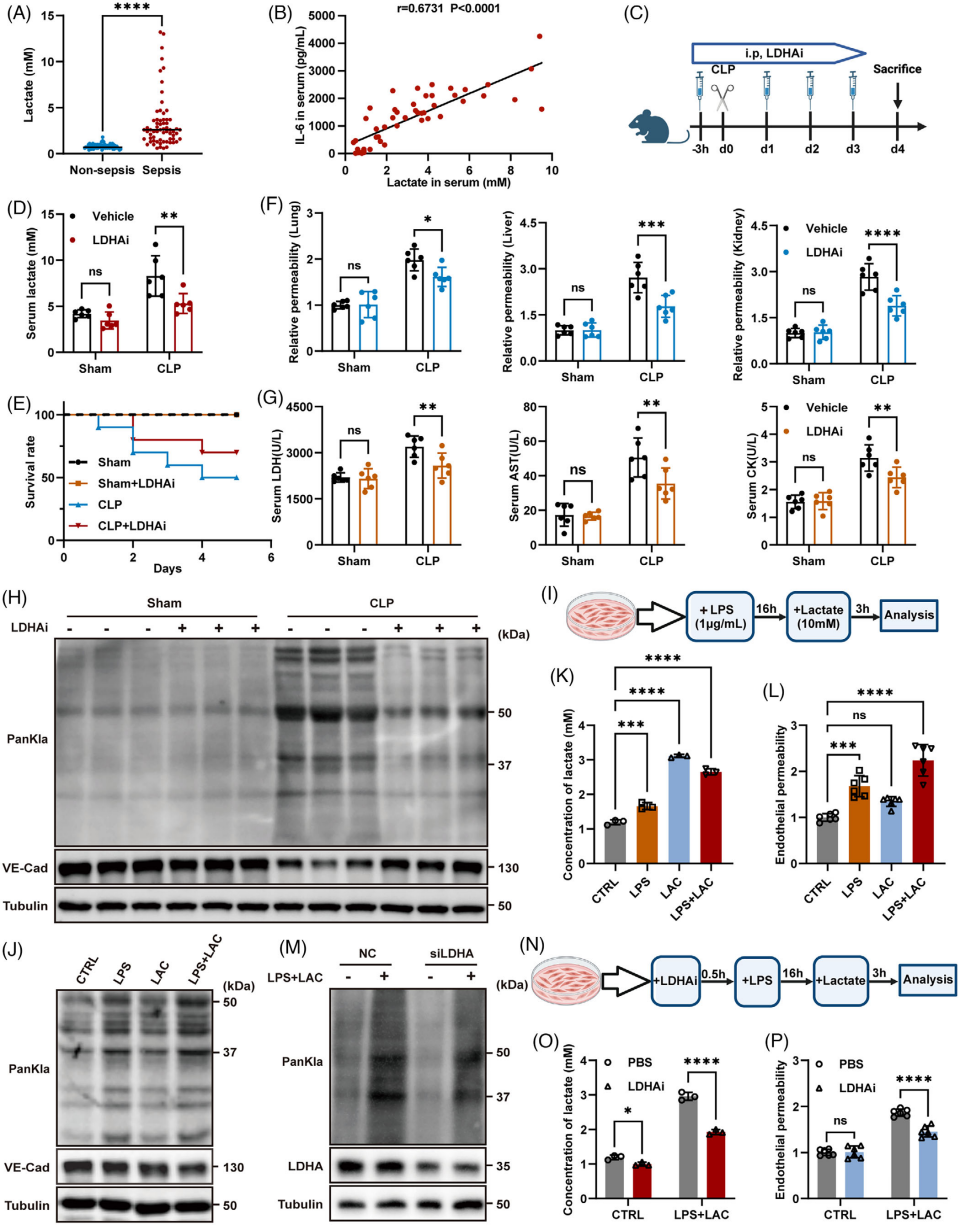
Lactylation‐induced vascular endothelial dysfunction in sepsis. (A) The levels of lactate in serum from sepsis patients (*n* = 65) and non‐sepsis controls (*n* = 65). (B) Correlation between IL‐6 levels and lactate levels in the serum from sepsis patients (*n* = 50). (C) Schematic illustration of CLP‐induced mouse model of sepsis generated by the administration of LDHAi. Age‐ and sex‐matched mice were administered with LDHAi (Oxamate, 750 mg/kg body weight) 3 h before CLP surgery and injected intraperitoneally for the following three days after modelling (schematic created with BioRender.com). (D) Serum lactate levels in CLP mice following LDHAi administration (*n* = 6 per group). (E) Survival rate of model mice after the administration of LDHAi (*n* = 10 per group). (F) Relative permeability of microvessels in the lungs, liver and kidneys in CLP mice following the administration of LDHAi as determined by Evans Blue Dye (EBD) absorbance at 610 nm (*n* = 6 per group). (G) LDH, AST and CK levels in serum of CLP mice (*n* = 6 per group). (H) Lactylation and VE‐Cadherin protein levels of blood vessels in CLP mice following LDHAi administration as detected by western blotting (*n* = 3 per group). (I) Schematic showing the experimental design in ECs. (J) Lactylation and VE‐Cadherin protein levels in HUVECs after stimulation with LPS and lactate were detected by western blotting. (K) Lactate levels in HUVECs (*n* = 3 per group). (L) Endothelial permeability(HUVECs) was detected by Transwell assays (*n* = 6 per group). (M) Changes of lactylation levels following the knockdown of LDHA in EA.HY926. (N) Schematic showing the experimental design of ECs following stimulation with LDHAi. (O) Lactate levels in HUVECs following stimulation with LDHAi (*n* = 3 per group). (P) Endothelial permeability (HUVECs) following LDHAi stimulation was detected by Transwell assays (*n* = 6 per group). Data are presented as the mean ± SD. ns, not significant, *p < .05, **p < .01, ***p < .001, ****p < .0001.

Previous clinical studies have demonstrated a strong association between elevated levels of lactate and the severity of sepsis.^12^, ^28^ Our analysis of serum lactate data from patients with sepsis showed that serum lactate levels were significantly higher in sepsis patients when compared to non‐septic controls (Figure 1A). Furthermore, serum lactate levels in sepsis patients were positively correlated with levels of the pro‐inflammatory cytokine IL‐6 (Figure 1B), thus indicating a link between lactate and the severity of inflammation in sepsis. To further investigate the role of lactate, we used a mouse model of CLP to replicate the clinical phenotype of sepsis. Analysis showed that serum lactate levels in the CLP group increased significantly with the progression of sepsis (Figure S1A). Furthermore, the survival rate of mice in the CLP group was significantly lower than that in the sham operation (Sham) group (Figure S1B). Haematoxylin and eosin staining demonstrated severe inflammatory infiltration and tissue damage in the lungs, liver, and kidneys of CLP mice (Figure S1C); this was accompanied by a significant increase in serum inflammatory cytokines (Figure S1D). Furthermore, upon administering EBD via tail vein injection, we observed increased microvascular permeability in the lungs, liver, and kidneys of CLP mice, thus suggesting that sepsis was associated with vascular damage(hepatic fenestrated capillaries may overestimate permeability changes, and thus results should be interpreted alongside non‐fenestrated organs, such as kidney and lung) (Figure S1E). Since elevated lactate levels are often accompanied by the lactylation of related proteins, we further investigated whether vascular injury in sepsis was also associated with this modification. Analysis of blood vessels in septic mice revealed significantly increased levels of lactylation and reduced expression levels of VE‐Cadherin, a key component of endothelial‐specific AJs, in the CLP group (Figure S1F). Immunofluorescence staining of mouse lung tissue provided further evidence of reduced VE‐Cadherin levels in CLP mice (Figure S1G). These findings suggested that the lactylation arising from high lactate levels may contribute to vascular damage in sepsis.

To investigate the effect of elevated lactate and lactylation in sepsis, we administered oxamic acid sodium (OXA), a lactate dehydrogenase (LDHA/LDH) inhibitor (LDHAi), to inhibit the production of lactate in septic mice^17^ (Figure 1C). By assessing the impact of different LDHAi doses on septic mouse survival and lactate levels, we established the optimal LDHAi dose (Figure S2). Under this dose, LDHAi significantly reduced serum lactate levels and improved the survival rate of septic mice (Figure 1D and E). Furthermore, LDHAi partially reduced microvascular permeability in CLP mice (Figure 1F) and reduced serum levels of LDH, AST, and CK, which are all markers of multi‐organ damage (Figure 1G). After inhibiting lactate production, we found that the extent of lactylation in blood vessels was significantly reduced and that VE‐Cadherin expression was restored (Figure 1H).

These findings indicate that in a high‐lactate environment, increased lactylation in the blood vessels of septic mice can lead to reduced VE‐Cadherin expression, thus resulting in increased vascular permeability and impaired microcirculation. Inhibiting lactylation by reducing serum lactate significantly ameliorated vascular injury and multiple organ failure in sepsis, thereby improving survival outcomes.

The activation and dysfunction of ECs are central to the pathophysiology of sepsis. Furthermore, the disruption of endothelial homeostasis is a key factor in vascular injury during sepsis, ultimately leading to dysfunction in multiple organs.^5^, ^6^ In the present study, we will investigate how elevated lactate and lactylation can impact endothelial function and homeostasis in sepsis. To simulate the high‐lactate environment of sepsis in vitro, we stimulated ECs with both LPS and lactate (Figure 1I). Analysis showed that when compared to LPS or lactate stimulation alone, the combined stimulation led to a more pronounced increase in lactylation (Figure S3A) and a greater reduction in VE‐Cadherin expression in ECs (Figure 1J), thus indicating more severe endothelial injury.

Furthermore, we observed that the expression of several other key proteins related to endothelial junctions was significantly reduced (Figure S3B), further confirming that the endothelial barrier had been disrupted. Furthermore, intracellular lactate levels in ECs increased significantly following LPS and lactate stimulation (Figure 1K). This led to increased endothelial permeability (Figure 1L), reduced cell proliferation (Figure S3C), and reduced cell migration (Figure S3D). Collectively, these results indicate significant endothelial dysfunction.

Next, we investigated whether inhibiting lactate production and reducing lactylation in ECs could restore endothelial function. The knockdown of LDHA significantly reduced lactylation in ECs (Figure 1M). Furthermore, we sought to inhibit LDHA enzyme activity pharmacologically to reduce lactylation (Figure 1N). By adding different concentrations of LDHAi, we observed a gradual reduction in the levels of lactylation in ECs (Figure S3E). At the optimal LDHAi concentration (5 mM), the intracellular level of lactate in ECs was significantly reduced (Figure 1O). Furthermore, endothelial permeability was reduced (Figure 1P), cell proliferation rate was increased (Figure S3F), and areas of migration had expanded (Figure S3G), thus indicating the restoration of endothelial function.

Notably, we found that lactylation persisted in ECs for some time, even after LPS and lactate stimulation had been discontinued (Figure S3H), thus suggesting that lactylation may have long‐lasting effects on ECs. These results indicated that the lactylation induced by elevated lactate levels can lead to endothelial injury. The inhibition of lactate production to reduce the extent of lactylation significantly improved endothelial function.

### 2.2 ENO1 was lactylated at K71 in ECs and contributed to lactate‐induced endothelial dysfunction

**FIGURE 2:**
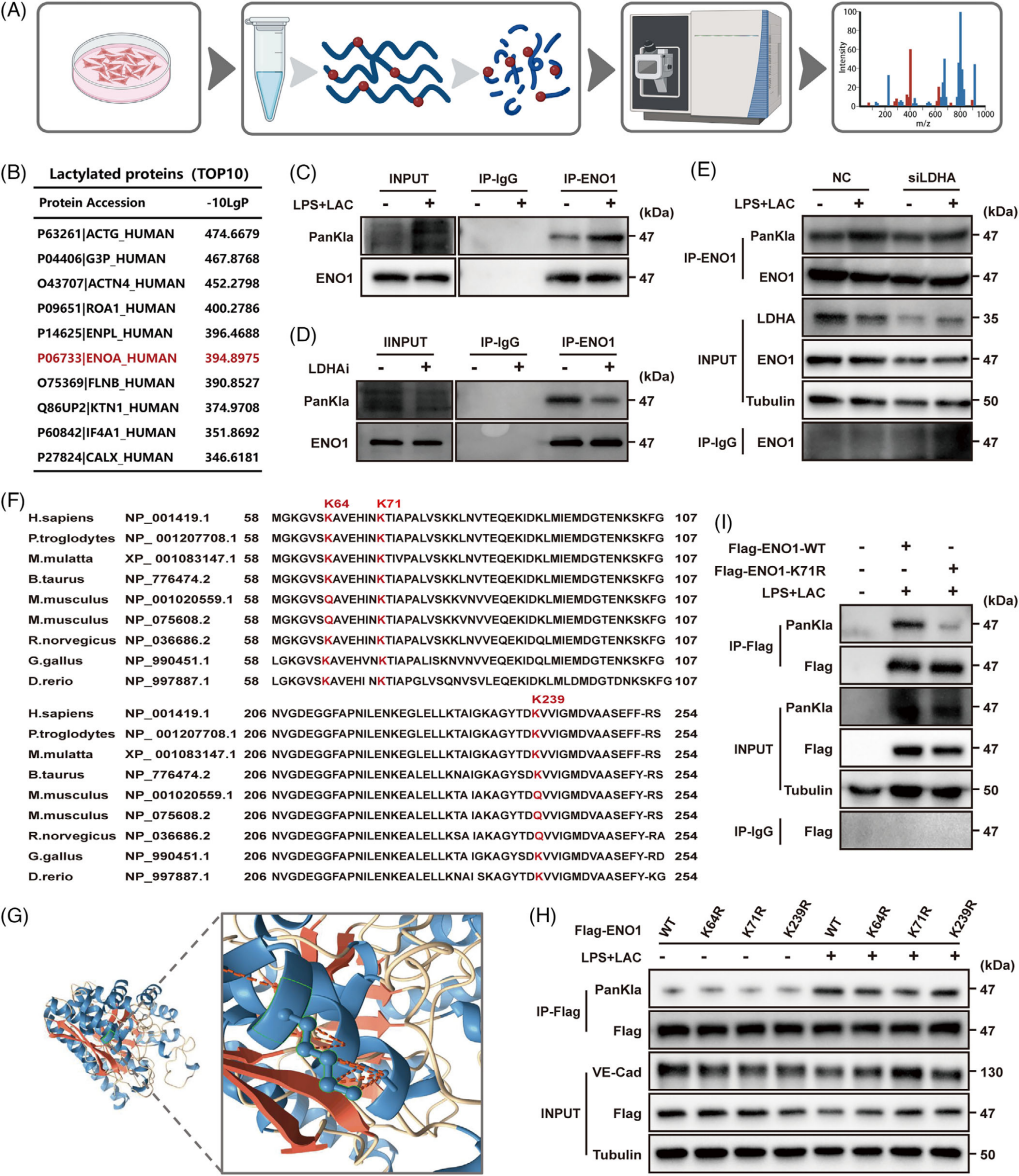
ENO1 K71 was identified as the key site of lactylation in ECs. (A) Schematic diagram showing the detection of lactylated protein by mass spectrometry (MS) (schematic created with BioRender.com). (B) Top 10 lactylated non‐histone proteins as detected by MS. ENO1 is marked in red. (C–E) The lactylation of ENO1 was detected under different conditions (C: LPS+Lactate stimulation; D: LDHAi stimulation; E: LDHA knockdown with siRNA) by western blotting after immunoprecipitation (IP). (F) Alignment of sequences around ENO1 K64, K71 and K239 from different species. The three lysines are marked in red. (G) Ribbon diagram of the crystal structure of human ENO1 protein at the K71 site. (H) The lactylation of ENO1 was detected after the overexpression of plasmids with different lysine mutations. (I) The lactylation of ENO1 was detected after the overexpression of Flag‐ENO1‐K71R plasmid in ENO1 knockdown ECs.

To further investigate the specific mechanism by which lactylation can exert effect on endothelial function, we next conducted LC‐MS/MS to identify lactylated proteins in ECs stimulated with LPS and lactate (Figure 2A). As research on lactylation has progressed, more non‐histone lactylation sites have been discovered beyond those originally detected in histones. The top 10 key proteins with the highest abundance of non‐histone lactylation sites are shown in Figure 2B. Additionally, we performed KEGG pathway analysis on the proteins identified through MS and found significant enrichment in the glycolysis pathway (Figure S4A). By integrating these proteomic results with lactylation profiling, we identified ENO1—a key glycolytic enzyme, as a likely contributor to endothelial dysfunction in sepsis. Moreover, previous studies have revealed that lactylation frequently occurs on enzymes associated with glycolysis, such as PKM and ALDOA.^29^, ^30^ Of these, ENO1 stands out as particularly noteworthy. However, the precise mechanism by which ENO1 exerts its effects in this study remains to be further elucidated.

Immunoprecipitation (IP) analysis revealed that the lactylation of ENO1 increased significantly following stimulation with LPS and lactate (Figure 2C), while the inhibition of intracellular lactate production caused a reduction in ENO1 lactylation (Figure 2D and E). Furthermore, the transcription of ENO1 did not change significantly, although the level of protein translation was slightly reduced (Figure S4B and C). Collectively, these results suggest that lactate does not affect the expression of ENO1 but instead modifies its lactylation.

IP‐MS analysis revealed three significant lactylation sites on ENO1: K64, K71, and K239. By analysing the structure of these sites, and their conservation across different species, we identified K71 as a key site for ENO1 lactylation (Figures 2F and G and S4D and E). Then, we constructed plasmids with mutations at these sites and found that the lactylation of ENO1 was most significantly reduced when the K71 site was mutated, further confirming that K71 represents the key site for ENO1 lactylation (Figure 2H).

Since high endogenous levels of ENO1 in ECs may influence the expression of exogenous ENO1, we generated ECs (EA.HY926) in which ENO1 had been knocked down and confirmed a significant reduction in the expression of ENO1 (Figure S4F). Using this cell line, we next verified that lactylation at the K71 site was suppressed after this specific site had been mutated (Figure 2I). Furthermore, we found that ENO1 enzyme activity was slightly reduced but not significantly different after stimulation with LPS plus lactate (Figure S4G), and that enzyme activity did not change significantly after K71 site mutation (Figure S4H), suggesting that lactylation did not influence ENO1 enzyme activity.

To further elucidate the role of ENO1, we constructed an adeno‐associated virus (AAV) shRNA specifically targeting ENO1 in vascular endothelial cells and administered it to mice via tail vein injection. This approach led to a marked reduction of ENO1 expression in blood vessels (Figure S5A). Compared to vehicle‐treated mice, those injected with AAV‐ENO1 exhibited significantly increased microvascular permeability in the liver, lungs and kidneys (Figure S5B). These findings suggest that ENO1 plays a critical role in maintaining endothelial barrier integrity and regulating vascular permeability.

### 2.3 P300 acted as the lactyltransferase mediating ENO1‐K71 lactylation in ECs

**FIGURE 3:**
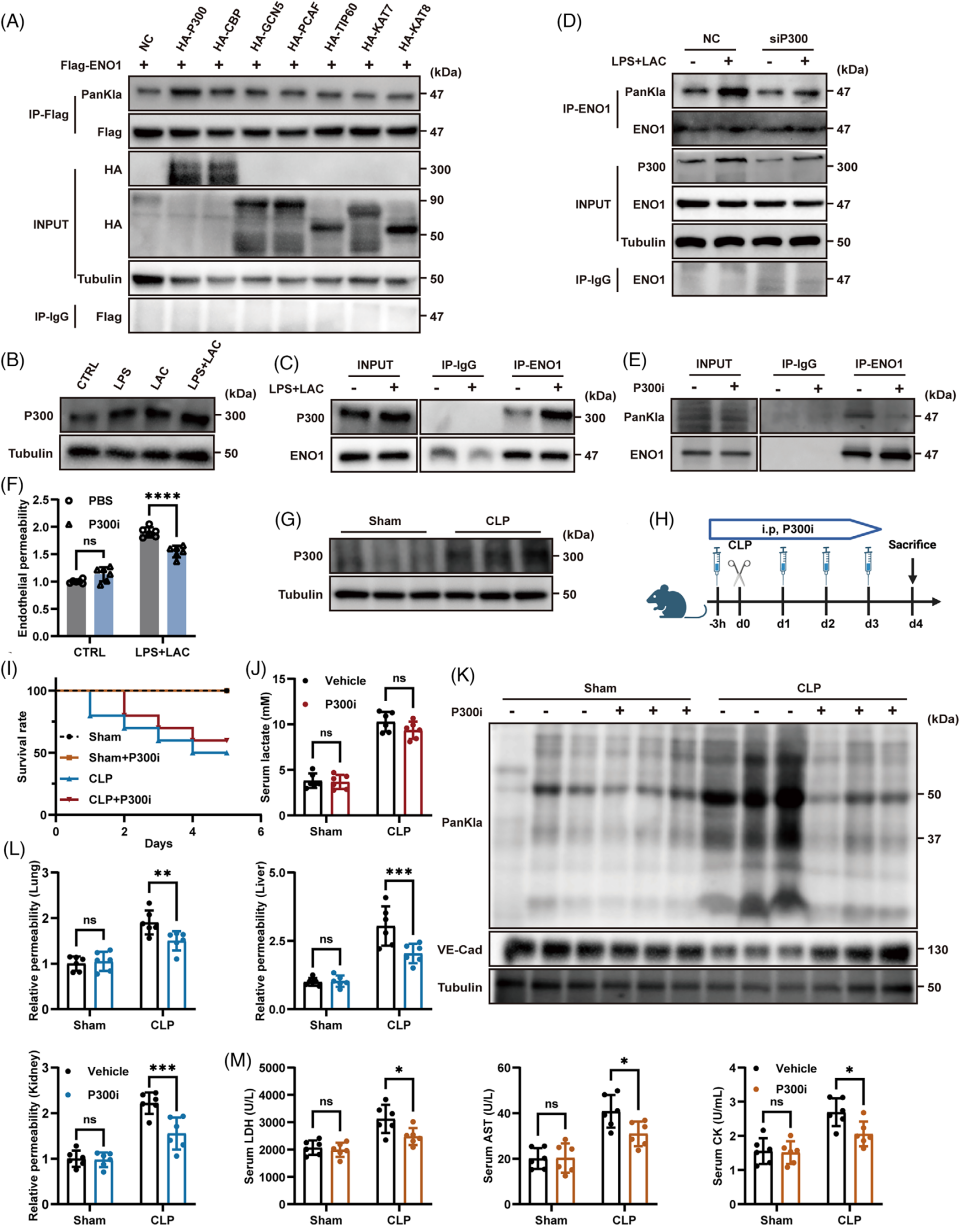
P300 promoted the lactylation of ENO1. (A) ENO1 and some acetyltransferases which were previously reported or potentially participating in lactylation were transfected simultaneously in ENO1 knock‐down ECs to screen the ‘writers’ of ENO1 lactylation. (B) Levels of P300 protein in HUVECs under different stimulation conditions, as detected by western blotting. (C) CoIP revealed interactions between P300 and ENO1. (D) Changes of ENO1 lactylation following P300 knockdown. (E) Changes of ENO1 lactylation following treatment with P300i. (F) Endothelial permeability following P300i treatment was detected by Transwell assays (*n* = 6 per group). (G) P300 protein levels in blood samples from mice. (H) Schematic illustration of a CLP‐induced mouse model of sepsis treated with P300i. Age‐ and sex‐matched mice were administered with P300i (C646, 30 mM/kg body weight) 3 h before CLP surgery and injected intraperitoneally for the next three days after modelling (schematic created with BioRender.com). (I) Survival rate of model mice following P300i administration (*n* = 10 per group). (J) Lactate levels in CLP mice following P300i administration (*n* = 6 per group). (K) Lactylation and VE‐Cadherin protein levels in the blood vessels of CLP mice following P300i administration were detected by western blotting (*n* = 3 per group). (L) Relative permeability of microvessels in the lungs, liver, and kidneys in CLP mice with P300i administration, as determined by Evans Blue Dye (EBD) absorbance at 610 nm (*n* = 6 per group). (M) Levels of LDH, AST and CK in serum samples from CLP mice (*n* = 6 per group). Data are presented as mean ± SD. ns, not significant, *p < .05, **p < .01, ***p < .001.

Next, we investigated the mechanisms responsible for the lactylation of ENO1. Recent studies have indicated that an increasing number of acetyltransferases, including P300 and TIP60, are involved in the lactylation of many proteins.^31^, ^32^ By constructing plasmids for various acetyltransferases (P300, CBP, GCN5, PCAF, TIP60, KAT7, KAT8), we found that P300 significantly promoted the lactylation of ENO1 (Figure 3A), thus suggesting that P300 may act as an upstream writer responsible for the lactylation of ENO1.

In addition, the expression level of P300 was significantly elevated following stimulation with LPS and lactate (Figure 3B, Figure S6A). CO‐IP analysis identified an interaction between ENO1 and P300 (Figure 3C). Immunofluorescence analysis also revealed colocalisation between the two proteins (Figure S6B‐C). Furthermore, following the knockdown of P300, we observed reduced levels of lactylation in ECs and ENO1, as well as increased expression of VE‐Cadherin (Figures S6D and E and 3D). These findings suggested that P300 may be the lactyltransferase that promotes the lactylation of ENO1 in ECs.

Next, we used C646, a P300 inhibitor (P300i), and found that increasing concentrations of P300i significantly inhibited the lactylation of ECs (Figure S6F).^31^, ^33^ At an optimal concentration of P300i (10 µM), ENO1 lactylation was also significantly suppressed (Figure 3E). Moreover, following P300i treatment, endothelial permeability was partially restored (Figure 3F), the cell proliferation rate increased (although not significantly), and areas of migration were expanded (Figure S6G and H). Collectively, these results suggest that the inhibition of P300 led to an improvement in endothelial function.

Further analysis revealed that P300 expression was significantly elevated in the vascular tissues of CLP mice (Figure 3G). To further investigate the effect of P300 on vascular endothelial lactylation in sepsis, we administered P300i via intraperitoneal injection in septic mice (Figure 3H). We first identified the optimal dose of P300i via a dose gradient (Figure S6I and J). Subsequent investigations revealed that that P300i significantly increased the survival rate of septic mice without affecting the survival of sham‐operated mice (Figure 3I). Furthermore, P300i did not influence serum lactate levels but significantly reduced lactylation in blood vessels while increasing the expression of VE‐Cadherin (Figure 3J and K).

Importantly, the administration of P300i partially reduced microvascular permeability in the lungs, liver, and kidneys of septic mice (Figure 3L). Moreover, after administering P300i, the serum levels of LDH, AST, and CK in septic mice all decreased, indicating a reduction in organ injury (Figure 3M).

Collectively, these findings suggested that P300 promoted the lactylation of ENO1 in ECs, leading to endothelial dysfunction and increased vascular permeability in sepsis. Furthermore, the inhibition of P300 significantly improved survival rates and alleviated disease symptoms in septic mice.

### 2.4 TRIM21 functioned as an E3 ubiquitin ligase that targeted VE‐Cadherin for proteasomal degradation in ECs

**FIGURE 4:**
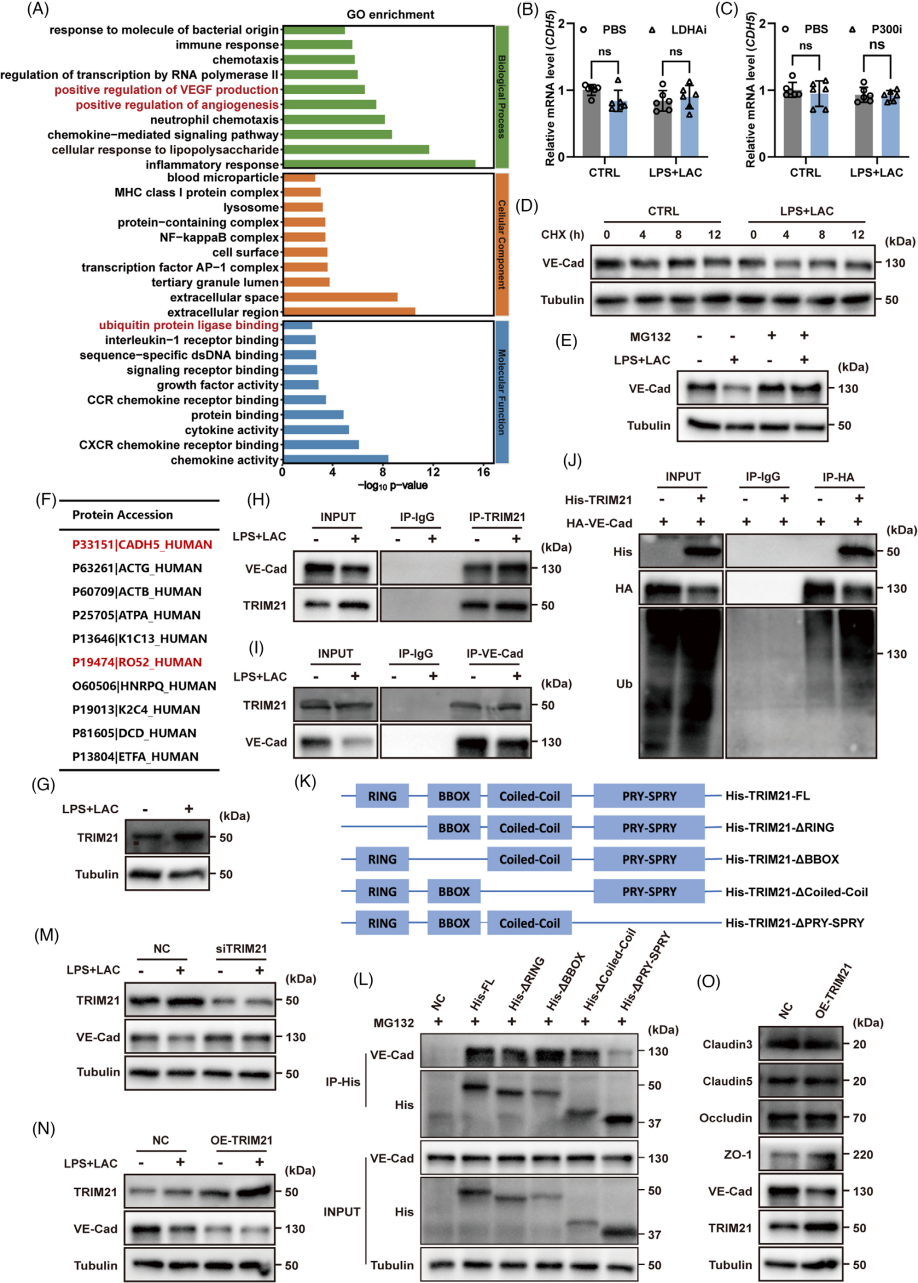
TRIM21 promoted the ubiquitination and degradation of VE‐Cadherin. (A) Gene ontology (GO) terms for the most enriched pathways in HUVECs following LPS and lactate treatment. (B, C) Expression levels of *CDH5* (VE‐Cadherin) mRNA under treatment with LDHAi and P300i in HUVECs, as determined by RT‐qPCR (*n* = 6 per group). (D) The degradation rate of VE‐Cadherin under CHX treatment was detected by western blotting. (E) VE‐Cadherin expression levels following treatment with MG132 were detected by western blotting. (F) Proteins binding to VE‐Cadherin were detected by IP‐MS. VE‐Cadherin (P33151) and TRIM21(P19474) are marked in red. (G) TRIM21 protein levels in ECs were detected by western blotting. (H, I) CoIP revealed interactions between TRIM21 and VE‐Cadherin in HUVECs. (J) CoIP revealed interactions between TRIM21 and VE‐Cadherin in 293T cells. (K) Schematic diagram showing the four functional domains of human TRIM21. (L) His‐tagged TRIM21 or mutant versions were transfected into EA.HY926. CoIP revealed interactions between VE‐Cadherin and different TRIM21 mutants. (M, N) VE‐Cadherin protein levels were detected after TRIM21 knockdown or overexpression. (O) The expression levels of endothelial barrier‐related proteins were detected after the overexpression of TRIM21. Data are presented as the mean ± SD. ns, not significant.

Based on the findings described above, we confirmed that P300 contributes to endothelial dysfunction by promoting lactylation of ENO1 at K71 sites. However, the specific mechanism by which the lactylation of ENO1‐K71 can influence endothelial function remained unclear. To address this, we performed RNA sequencing (RNA‐seq) on ECs following stimulation with LPS and lactate. RNA‐seq revealed the differential expression of genes related to vascular stability and ubiquitination pathways (Figure 4A). Further analysis indicated that the level of ubiquitination increased following stimulation with LPS and lactate, while treatment with the P300 inhibitor (P300i) significantly reduced the extent of ubiquitination in ECs (Figure S7A).

Furthermore, the expression levels of VE‐Cadherin protein were significantly increased (Figures 1H and 3K), although transcription did not change significantly when stimulated by LDHAi and P300i (Figure 4B and C), thus suggesting that the expression of VE‐Cadherin is regulated post‐transcriptionally. Ubiquitination is a common PTM. To determine whether the reduction in VE‐Cadherin levels was due to ubiquitination, we next compared VE‐Cadherin protein levels in ECs following treatment with cycloheximide (CHX), an inhibitor of eukaryotic protein synthesis. A significant reduction in the expression of VE‐Cadherin was detected following stimulation with LPS and lactate (Figure 4D). In contrast, treatment with MG132, a proteasome inhibitor, inhibited the degradation of VE‐Cadherin (Figure 4E). These results suggest that VE‐Cadherin undergoes ubiquitination and is degraded via the proteasome pathway. Next, we used IP‐MS to identify potential E3 ubiquitin ligases involved in the ubiquitination of VE‐Cadherin (P33151); this analysis led to the identification of TRIM21 (P19474) (Figures 4F and S7B). TRIM21, a member of the tripartite motif‐containing (TRIM) family, is a common E3 ubiquitin ligase that plays crucial roles in various diseases by ubiquitinating and degrading multiple proteins.^34^, ^35^ The expression level of TRIM21 in ECs was significantly increased following stimulation with LPS and lactate (Figure 4G). Furthermore, CO‐IP demonstrated an interaction between TRIM21 and VE‐Cadherin (Figures 4H and I). Immunofluorescence analysis also revealed colocalisation between TRIM21 and VE‐Cadherin (Figure S7C and D). Further validation in HEK293T cells confirmed that TRIM21 binds to and ubiquitinates VE‐Cadherin, thus leading to its degradation (Figure 4J). Additional, the combination of MG132 and ENO1‐K71R significantly restored VE‐Cadherin expression in ECs (Figure S6E). This further suggests that inhibition of the ubiquitination process and lactylation at the ENO1‐K71 site can preserve the integrity of the vascular endothelial barrier.

Specifically, in TRIM21‐KO ECs stimulated with LPS and lactate, overexpression of ENO1‐WT or ENO1‐K71R alone did not affect VE‐Cadherin expression. Notably, only upon TRIM21 re‐expression did ENO1‐K71R exhibit a protective effect, characterised by increased VE‐Cadherin expression. These results demonstrate that the functional consequence of ENO1‐K71 lactylation is TRIM21‐dependent (Figure S6F).

In accordance with previous studies, TRIM21 contains four major functional domains.^36^ We found that the PRY‐SPRY domain was crucial for the specific interaction between TRIM21 and VE‐Cadherin (Figure 4K and L). The knockdown of TRIM21 in ECs led to a significant increase in the protein levels of VE‐Cadherin (Figure 4M), whereas the overexpression of TRIM21 led to a significant reduction of VE‐Cadherin expression without affecting other tight junction proteins (Figure 4N and O). To further validate the function of TRIM21 in maintaining ECs function, LPS‐induced sepsis model was done with TRIM21 knockout (TRIM21‐KO) mice. Compared to wild‐type (WT) controls, TRIM21‐KO mice exhibited significantly increased expression of VE‐Cadherin in blood vessels, along with reduced microvascular permeability in the liver, lungs and kidneys following LPS‐induced sepsis (Figure S8A and B). These findings indicate that TRIM21 deletion alleviates endothelial injury and partially restores the endothelial barrier in septic mice.

Collectively, these results suggest that in sepsis, the elevation of TRIM21 contributes to increased endothelial permeability by promoting the ubiquitination and degradation of VE‐Cadherin, thereby disrupting vascular homeostasis.

### 2.5 ENO1‐K71 lactylation enhanced *TRIM21* mRNA stability by disrupting ENO1–CNOT6–mediated RNA degradation

**FIGURE 5:**
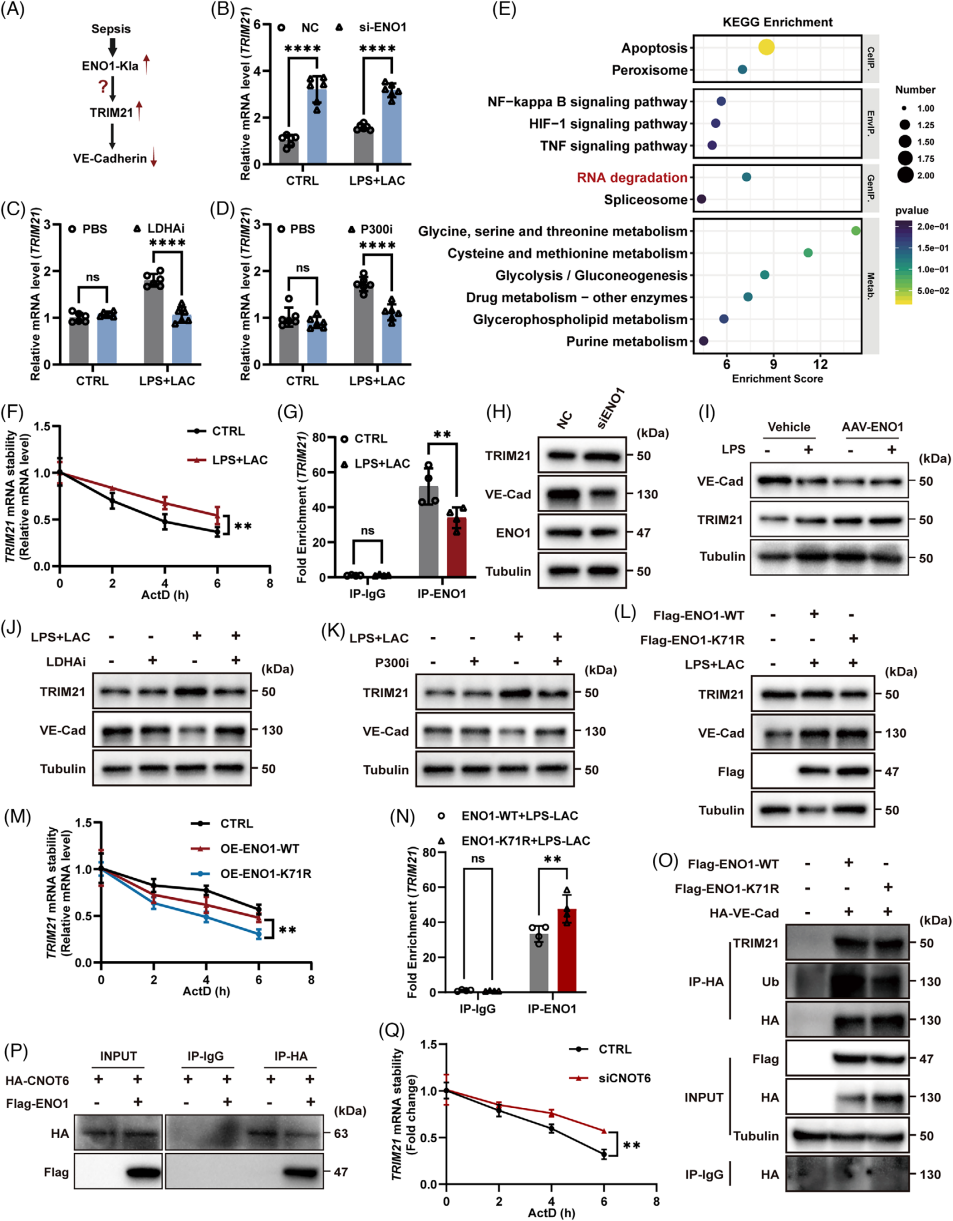
Lactylation of ENO1‐K71 enhanced the stability of *TRIM21* mRNA. (A) A schematic depicting the current problem to be solved (ENO1‐Kla: Lactylation of ENO1). (B–D) *TRIM21* mRNA expression levels under different conditions in HUVECs, as determined by RT‐qPCR (*n* = 6 per group). (E) KEGG enrichment showed that the RNA degradation pathway was enriched after ENO1 knockdown in HUVECs. (F) *TRIM21* mRNA stability in HUVECs when treated with actinomycin D (ActD) at the indicated time points, as determined by RT‐qPCR (*n* = 6 per group). (G) The levels of *TRIM21* mRNA combined to ENO1 protein were detected by RIP‐qPCR (*n* = 4 per group). (H) TRIM21 and VE‐Cadherin protein levels were detected by western blotting following the knockdown of ENO1. (I) VE‐Cadherin and TRIM21 protein levels of blood vessels in Vehicle‐treated and AAV‐ENO1‐treated mice following LPS administration were detected by western blotting. (J, K) TRIM21 and VE‐Cadherin protein levels were detected by western blotting following LDHAi and P300i treatment. (L) TRIM21 and VE‐Cadherin protein levels were detected by western blotting after overexpressing mutated or normal ENO1 plasmids. (M) *TRIM21* mRNA stability in ECs overexpressing different plasmids and treated with actinomycin D (ActD) at the indicated times points, as determined by RT‐qPCR (*n* = 6 per group). (N) The levels of *TRIM21* mRNA combined to ENO1 protein were detected by RIP‐qPCR after overexpressing mutated or normal ENO1 plasmids (*n* = 4 per group). (O) TRIM21 and VE‐Cadherin protein levels were detected after immunoprecipitation in 293T cells. (P) HA‐CNOT6 plasmid and Flag‐ENO1 plasmid were transfected in EA.HY926, and Co‐IP revealed interactions between CNOT6 and ENO1. (Q) *TRIM21* mRNA stability in EA.HY926 when treated with actinomycin D (ActD) at the indicated time points, as determined by RT‐qPCR (*n* = 6 per group). Data are presented as the mean ± SD. ns, not significant, *p < .05, **p < .01, ****p < .0001.

Our previous experiments demonstrated that both the lactylation of ENO1‐K71 and the elevation of TRIM21 could reduce the expression of VE‐Cadherin. Next, we investigated the specific relationship between ENO1‐K71 lactylation and TRIM21 expression (Figure 5A). We found that the knockdown of ENO1 in ECs resulted in a significant increase in the levels of *TRIM21* mRNA (Figure 5B). However, when ENO1‐K71 lactylation was inhibited by LDHAi and P300i, we observed a significant reduction in the levels of TRIM21 mRNA (Figure 5C and D). These findings suggest that the lactylation of ENO1‐K71 promoted the expression of *TRIM21* mRNA.

KEGG enrichment analysis further revealed that the knockdown of ENO1 exerted effect on RNA degradation pathways (Figure 5E). As an RNA‐binding protein, ENO1 is known to bind to RNA and influence stability.^25^, ^27^ Actinomycin D (ActD) assays revealed that the stability of *TRIM21* mRNA increased following stimulation with LPS and lactate (Figure 5F). RNA pull‐down assay demonstrated that *TRIM21* mRNA binds to ENO1 protein in ECs (Figure S9A). Subsequent RIP‐qPCR analysis revealed that ENO1 binds to TRIM21 mRNA, and that the extent of this binding decreased following stimulation with LPS and lactate (Figure 5G). Furthermore, the knockdown of ENO1 significantly increased the expression of TRIM21 but reduced the expression of VE‐Cadherin in ECs (Figure 5H). Similarly, specific deletion of ENO1 in vascular endothelial cells using AAV‐ENO1 resulted in a marked increase in TRIM21 expression and an decrease in VE‐Cadherin expression in blood vessels of mice (Figure 5I). However, when ENO1 lactylation was inhibited by P300i or LDHAi, the opposite effects were observed (Figure 5J and K). Collectively, these results indicated that the lactylation of ENO1 influences the binding of ENO1 to *TRIM21* mRNA, thereby affecting VE‐Cadherin ubiquitination. To further verify the specific relationship between the lactylation of ENO1‐K71 and *TRIM21* mRNA, we overexpressed both the wild‐type (WT) ENO1 plasmid and a K71‐mutated plasmid (K71R) in ENO1‐knockdown ECs. Subsequent analysis revealed that the mutation of ENO1‐K71 significantly reduced the levels of TRIM21 and increased the levels of VE‐Cadherin (Figure 5L). Moreover, the inhibition of ENO1‐K71 lactylation by mutation led to a reduction in the stability of *TRIM21* mRNA, thus accelerating its degradation (Figure 5M). Importantly, the mutation of ENO1‐K71 enhanced the binding of ENO1 to *TRIM21* mRNA (Figures 5N and S9B). In HEK293T cells and ENO1‐KD ECs, we further confirmed that the mutation of ENO1‐K71 reduced the expression of TRIM21 and reduced the ubiquitination of VE‐Cadherin (Figures 5O and S9C).

According to previous study, ENO1 has been shown to recruit the deadenylase CCR4‐NOT transcription complex subunit 6 (CNOT6) to the 3′ UTR of mRNA, which plays a crucial role in mRNA degradation by removing poly (A) tails of mRNA.^27^ To investigate whether ENO1 similarly recruits CNOT6 to mediate the degradation of *TRIM21* mRNA, we conducted a series of experiments. Co‐IP confirmed a clear interaction between ENO1 and CNOT6 (Figure 5P). Knockdown of CNOT6 using siRNA led to an increase both in *TRIM21* mRNA level and protein level, while overexpression of CNOT6 partially reduced TRIM21 protein expression (Figure S8A–D). Furthermore, actinomycin D (ActD) chase experiment showed that CNOT6 knockdown enhanced the stability of *TRIM21* mRNA, indicating that CNOT6 plays a crucial role in *TRIM21* mRNA degradation (Figure 5Q). Significantly, compared to ENO1‐WT, ENO1‐K71R maintained a stronger binding with CNOT6 under LPS and lactate stimulation (Figure S10E). These results indicate that lactylation at the K71 site disrupts ENO1–CNOT6 complex formation.

Therefore, these findings elucidated the mechanism by which the lactylation of ENO1‐K71 can influence VE‐Cadherin ubiquitination by altering the stability of *TRIM21* mRNA.

### 2.6 Inhibiting ENO1‐K71 lactylation restored endothelial function and alleviates sepsis‐induced injury

**FIGURE 6:**
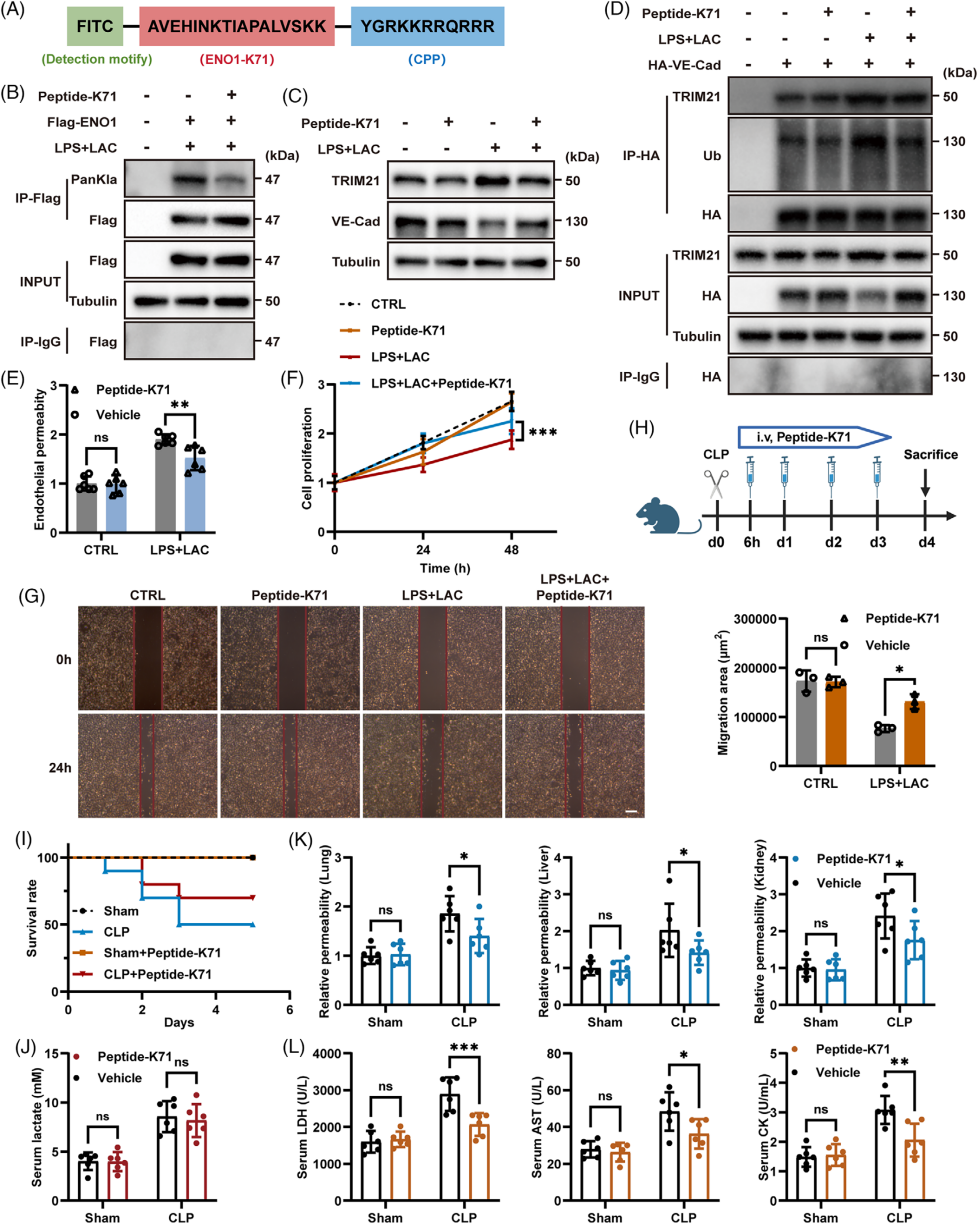
Targeting the lactylation of ENO1 K71 with a peptide inhibitor alleviated endothelial dysfunction in sepsis. (A) Schematic illustration showing the design of Peptide‐K71. Green: fluorescent modification (FITC, as detection modification). Red: inhibitory sequences for ENO1 lactylation (ENO1‐K71). Blue: cell‐penetrating peptide (CPP). (B) Lactylation levels of ENO1 when treated with Peptide‐K71 as detected by western blotting after immunoprecipitation. (C) TRIM21 and VE‐Cadherin protein levels under Peptide‐K71 treatment were detected by western blotting. (D) TRIM21 and VE‐Cadherin protein levels under Peptide‐K71 treatment were detected after immunoprecipitation in 293T cells. (E) Endothelial permeability following Peptide‐K71 treatment was detected in Transwell systems (*n* = 6 per group). (F) Cell proliferation of HUVECs after Peptide‐K71 treatment was determined by CCK8 absorbance at 450 nm (*n* = 5 per group). (G) Cell migration areas of HUVECs were measured after 24 h with Peptide‐K71 treatment (on the left is the migration image, and on the right is the quantitative analysis). (H) Schematic illustration of CLP‐induced mouse model of sepsis and Peptide‐K71 treatment. Age‐ and sex‐matched mice were administered with Peptide‐K71 (3 mg/kg body weight) 3 h before CLP surgery and injected intraperitoneally for the next three days after modelling. (I) Survival rate of model mice following Peptide‐K71 administration (*n* = 10 per group). (J) Serum lactate levels in mice following Peptide‐K71 administration (*n* = 6 per group). (K) Relative permeability of microvessels in the lungs, liver and kidneys of mice as determined by EBD absorbance at 610 nm (*n* = 6 per group). (L) levels of LDH, AST and CK levels in the serum of CLP mice (*n* = 6 per group). Data are presented as mean ± SD. ns, not significant, *p < .05, **p < .01, ***p < .001.

Our previous experiments suggested that targeting LDHA or P300 can inhibit the lactylation of ENO1 in ECs, thereby improving survival in a mouse model of sepsis. However, given the broad functional roles of LDHA and P300, which are both involved in various signalling pathways,^37^, ^38^ it was then necessary to find inhibitors that can specifically target the lactylation of ENO1‐K71. Based on previous studies relating to more established PTM intervention strategies, such as ubiquitination, phosphorylation, and glycosylation, along with a recent report on lactylation interventions,^19^, ^39^, ^40^, ^41^ we designed a specific short peptide (Peptide‐K71). This peptide competes with the ENO1‐K71 site for lactylation in ECs, thereby inhibiting lactylation at this site in vivo (Figures 6A and S10A). According to Peptide‐K71 sequence, we also constructed a control sequence in which lysine (K) at the 71 site was mutated to arginine (R) (Figure S10B).

Since Peptide‐K71 was tagged with a FITC fluorescent modification, we first used flow cytometry to determine the optimal concentration and treatment duration required for the peptide to enter cells (Figure S10C and D). The treatment of ECs with Peptide‐K71 significantly inhibited the lactylation of ENO1, indicating that Peptide‐K71 was specific to this modification (Figure 6B). Additionally, Peptide‐K71 reduced the expression of TRIM21 and subsequently reduced the ubiquitination of VE‐Cadherin (Figure 6C and D). Furthermore, after treatment with Peptide‐K71, endothelial permeability decreased, cell proliferation rate increased, and areas of migration expanded, thus indicating an improvement in endothelial function (Figure 6E–G).

To further validate the specificity of ​Peptide‑K71, we performed in vitro competitive and saturation experiments (Figure S12A). Results showed that Peptide‐K71 selectively inhibits ENO1‐K71 lactylation and downstream TRIM21 induction in ENO1‐WT overexpressing ECs, while these effects are abolished in ENO1‐K71R overexpressing ECs, demonstrating Peptide‐K71‐dependent specificity and on‐target efficacy. To address whether the effects of Peptide‐K71 are TRIM21‐dependent, we tested Peptide‐K71 in TRIM21‐KO cells/mice and observed that its protective effects on VE‐Cadherin stability were markedly attenuated (Figure S12B and C). In TRIM21‐KO ECs, replenishing TRIM21 also confirmed that the effect of Peptide‐K71 depends on TRIM21 (Figure S12D).

Finally, we evaluated the efficacy of Peptide‑K71 in a sepsis mouse model. We administered different specific dose of Peptide‐K71 to CLP mice via tail vein injection (Figure 6H). Based on serum lactate level measurements and survival outcomes, we determined the optimal dose of peptide‐K71 (Figure S13A and B). At this dosage, we measured the plasma concentration of Peptide‐K71 at different time points after administration using LC‐MS/MS (Figure S13C). Advanced analysis showed that Peptide‐K71 improved the survival rate of CLP mice to some extent, but did not induce a significant change in serum lactate levels (Figure 6I and J). Furthermore, microvascular permeability in CLP mice decreased, and the serum levels of LDH, AST, and CK were partially reduced (Figure 6K and L). Collectively, these findings indicate that Peptide‐K71 was able to alleviate vascular injury and multiple organ failure in septic mice, thereby improving survival outcomes. This finding provides a promising new therapeutic direction for the clinical treatment of patients with sepsis.

## 3 DISCUSSION

ECs are often considered as unconventional immune cells. During sepsis, a systemic infection, these cells tend to become excessively activated, thus resulting in impaired microcirculation, inadequate tissue perfusion, and life‐threatening organ failure. VE‐Cadherin, a vascular‐specific protein, is a key component of endothelial AJs and plays a crucial role in maintaining the integrity of endothelial connections.^10^ During sepsis, damage‐associated molecular patterns (DAMPs) and various pro‐inflammatory cytokines disrupt the functionality of the endothelial barrier by inducing the endocytosis and degradation of VE‐Cadherin.^42^, ^43^ For example, our previous studies have shown that IL‐6 can induce endothelial dysfunction.^44^ To further elucidate the impact of IL‐6 on endothelial integrity, we stimulated HUVECs with increasing concentrations of IL‐6. As shown in Figure S14, IL‐6 stimulation led to a dose‐dependent reduction in VE‐Cadherin expression, increased endothelial permeability, and decreased cell proliferation. These findings provide direct evidence that IL‐6 contributes to endothelial dysfunction. As shown in Figure 1B, we found that serum lactate levels in sepsis patients were positively correlated with levels of IL‐6. Taken together, these results provide additional evidence that lactate plays a critical role in the development of endothelial dysfunction.

Other research has shown that ECs can transport lactate via GPR81 and monocarboxylate transporters (MCTs).^16^, ^17^ As the specific role of lactate is gradually being elucidated, studies have shown that elevated levels of lactate in sepsis can also contribute to the degradation of VE‐Cadherin, thereby increasing endothelial permeability.^16^, ^17^ However, given the broad and diverse effects of lactate, the specific mechanisms by which lactate metabolism leads to the degradation of VE‐Cadherin and endothelial dysfunction remain unclear. In the present study, we found that the lactylation induced by lactate in ECs could promote the ubiquitination and degradation of VE‐Cadherin, thus providing new insights into how lactate contributes to the degradation of VE‐Cadherin and endothelial permeability.

As a newly discovered PTM, lactylation is been increasingly recognised for its role in a variety of diseases. Notably, in the high‐lactate environment characteristic of sepsis, lactylation levels are significantly elevated, thus contributing to lung inflammation and acute kidney injury by influencing the functionality of various proteins.^45^, ^46^ Furthermore, during sepsis, macrophages secrete exosomes containing lactylated and acetylated HMGB1; these exosomes can disrupt junctional proteins, such as VE‐Cadherin and Claudin‐5, resulting in endothelial dysfunction.^23^ However, the precise mechanisms underlying this disruption remain unclear. In our study, the focus is on ECs. Notably, we observed that endothelial lactylation persisted transiently after withdrawal of LPS and lactate stimulation in vitro. This finding does not conflict with the clinical improvement of vascular permeability following lactate clearance. Vascular recovery represents a system‐level outcome, whereas lactylation is a cell‐intrinsic PTM with stable regulatory properties.

Specially, we found that elevated lactate induces the lactylation of ENO1, which subsequently affects VE‐Cadherin expression and contributes to endothelial dysfunction. In addition to its role in glycolysis, ENO1 can also function as an RNA‐binding protein, participating in the regulation of RNA stability. Over recent years, an increasing body of research has focused on the RNA‐binding function of ENO1.^25^, ^27^ In septic ECs, we found that the lactylation level of ENO1 was significantly elevated, with the K71 site being a key location for this modification. From the perspective of RNA binding, we observed that ENO1 can bind to the mRNA of the E3 ubiquitin ligase TRIM21, thus resulting in reduced levels of *TRIM21* mRNA and subsequent degradation. However, the lactylation at the K71 site reduced the ability of ENO1 to bind and degrade *TRIM21* mRNA, thus leading to the increased expression of TRIM21. From the perspective of enzymatic activity, we found that lactylation did not influence the enzyme activity of ENO1; we hypothesise that this may be related to the K71 site not being located in the active regulatory region of the enzyme. However, some studies have reported the RNA‐binding ability of that ENO1 can influence its enzymatic activity and alter the concentrations of glycolytic metabolites.^25^ This discrepancy may be attributed to differences in the RNA‐binding sites of ENO1 or the different cell types involved. Further research is now needed to determine the factors that can regulate the enzymatic activity of ENO1 in septic ECs.

In our study, TRIM21, was regulated by ENO1 lactylation and could bind to VE‐Cadherin, leading to its ubiquitination and degradation. This discovery provides a new perspective on the reduction in VE‐Cadherin expression. Previous studies, using TRIM21 knockout mice, reported that the inflammatory response was significantly weakened, and that survival rates were notably improved during LPS‐induced sepsis.^36^ Furthermore, TRIM21 has been shown to promote the release of inflammatory factors such as IL‐1β by mediating the ubiquitination and degradation of SIRT5.^35^ IL‐1β can reduce the production of cAMP and CREB‐mediated VE‐Cadherin transcription, thus leading to pulmonary vascular endothelial damage in acute respiratory distress syndrome (ARDS).^47^ In the present study, we showed that the combined stimulation of LPS and lactate did not affect VE‐Cadherin transcription but rather altered its degradation at the protein level. The discrepancy between our findings and previous research may be due to the impact of lactate. Studies have shown that lactate can alter the protein levels of VE‐Cadherin without affecting its transcription,^17^ thus suggesting that lactate and inflammatory factors can influence VE‐Cadherin via different pathways. This further highlights the critical role of lactate and lactylation in the regulation of endothelial function. In addition to TRIM21, other E3 ubiquitin ligases have also been reported to be involved in the degradation of VE‐Cadherin. For instance, p120‐catenin regulates the endocytosis and degradation of VE‐Cadherin induced by Kaposi's sarcoma‐associated ubiquitin ligase K5.^48^ Tiruppathi et al. demonstrated that CHFR mediated the K48 ubiquitination of VE‐Cadherin in septic mice.^43^ Given that VE‐Cadherin contains multiple ubiquitination sites, it may undergo different types of ubiquitination mediated by various E3 ubiquitin ligases. However, our study did not specifically investigate the sites and types of ubiquitination of VE‐Cadherin mediated by TRIM21; these omissions need to be addressed by future research.

P300 functions as a pleiotropic enzyme, regulating various post‐translational modifications, including histone acetylation, and influencing multiple signalling cascades.^49^, ^50^ Therefore, we cannot rule out the possibility that the phenotypic changes observed following P300 inhibition may be partially attributed to broader biological effects independent of ENO1 lactylation. We clarify that P300 inhibition in our study serves primarily as a mechanistic tool to demonstrate the involvement of P300‐mediated lactylation in endothelial dysfunction, rather than as a pathway‐specific therapeutic intervention.

With the advancement and maturation of peptide synthesis technology, and the discovery of various PTMs in fundamental research, the artificial synthesis of specific peptides has gradually become a key strategy for manipulating protein PTMs.^19^, ^39^, ^40^, ^41^, ^51^ Based on these studies, we designed and synthesised Peptide‐K71 in an attempt to specifically inhibit ENO1‐K71 lactylation. In both in vivo and in vitro experiments, Peptide‐K71 significantly improved endothelial function in sepsis by inhibiting ENO1‐K71 lactylation, and increased the survival rate of septic mice, thus demonstrating promising potential for future applications. However, Peptide‐K71 is currently positioned as a proof‐of‐concept tool rather than a fully optimised therapeutic candidate. As an artificially designed molecule, Peptide‐K71 requires further in‐depth research. First, we did not perform an unbiased, lactylome‐wide analysis; therefore, potential effects of Peptide‐K71 on other lactylated proteins cannot be fully excluded. Such comprehensive specificity assessments were beyond the scope of the present study but will be essential in future work. Second, although MS‐based measurements of plasma Peptide‐K71 concentrations at multiple time points provide preliminary evidence of in vivo exposure, a complete pharmacokinetic and biodistribution profile—including tissue distribution, metabolic clearance, and half‐life—was not established. Future studies will focus on peptide optimisation and rigorous evaluation of specificity, pharmacokinetics, and safety to support further translational development. We believe that clearly defining these limitations enhances the transparency and rigor of the present study.

In summary, we found that an elevated lactate level in sepsis promotes the lactylation of ENO1 K71 in ECs via the action of P300. Lactylation at the ENO1 K71 site reduced the binding of *TRIM21* mRNA to ENO1, thus leading to the reduced degradation of *TRIM21* mRNA and increased protein expression. Further research revealed that TRIM21 could bind to VE‐Cadherin and exert E3 ubiquitin ligase functionality, causing the ubiquitination and degradation of VE‐Cadherin. This degradation disrupted endothelial AJs and increased endothelial permeability. The targeted reduction of lactylation at ENO1‐K71 using inhibitory peptides led to an improvement of endothelial function to some extent and alleviated sepsis‐related symptoms, potentially providing a therapeutic benefit for the clinical treatment of sepsis.

## 4 METHODS

### 4.1 Study patients

This clinical study was approved by the Research Ethics Board of the Children's Hospital of Fudan University in Shanghai, China (approval reference number: 2023 (249)). Sixty five  patients under the age of 18 year and diagnosed with sepsis based on the Sepsis‐3 criteria, were recruited from the paediatric Intensive Care Unit of the Children's Hospital of Fudan University. Two milliliters of whole blood were collected from each patient within 24 h of the sepsis diagnosis. Age‐ and sex‐matched healthy children were recruited as controls. Serum obtained from the whole blood samples was analysed to determine the levels of inflammatory factors (IL‐6) and lactate.

### 4.2 Mice

Based on previous research, male are more prone to inflammatory storms and immunosuppression than female when sepsis occurs; therefore, we selected male mice for this study.^52^, ^53^ C57BL/6J male mice (6–8 weeks‐of‐age) were purchased from SLAC Laboratory Animal Co. TRIM21‐KO mice were obtained from Shanghai Model Organisms. Experimental mice were housed, bred, and maintained under specific pathogen‐free conditions, with a 12‐h light/dark cycle. All experiments were conducted in compliance with relevant laws and institutional guidelines, under the supervision of the Animal Studies Committee of the Children's Hospital of Fudan University.

### 4.3 The mouse model of CLP

CLP were performed to induce sepsis, as previously described.^54^ First, 2.5% Avodin (15 mL/kg body weight) was administered for abdominal anaesthesia, and the abdominal wall was incised by 1–2 cm layer‐by‐layer to expose the cecum. The lower cecum was then ligated using 6‐0 surgical silk thread at 1/3 to 1/2 of the distance from the tip of the cecum, followed by puncture with a 20‐gauge needle. After allowing a small volume of fecal contents to extrude, the cecum was repositioned into the abdominal cavity, and the abdominal wall was closed layer‐by‐layer. Mice in the sham group underwent the same surgical procedure but without CLP. All mice were rehydrated by the intraperitoneal injection of .9% normal saline at the end of the procedure.

### 4.4 LPS‐induced mouse model

Mice in the experimental groups were injected intraperitoneally with 200 µL of LPS (30 mg/kg) dissolved in sterile PBS. Mice in the control group received intraperitoneal injections of an equivalent volume of PBS.

### 4.5 Cell culture and treatments

EA.HY926 cells and 293T cells were purchased from the Cell Bank, Shanghai Institute for Biological Science, Chinese Academy of Science, and cultured in DMEM medium with 10% fetal bovine serum and 1% PS. Human Umbilical Vein Endothelial cells (HUVECs) were purchased from the American Type Culture Collection and cultured in specific Endothelial Cell Growth Medium. Cell lines were authenticated using the profiles of short tandem repeats reported within the last three years. All cells were maintained in humidified cell incubators in 5% CO_2_ at 37°C. EA.HY926 and HUVECs were stimulated with 10 mM L‐lactate and 1 µg/mL of LPS for a certain period of time. In other experiments, OXA, C646, and Peptide‐K71 were stimulated .5 h earlier than L‐lactate.

### 4.6 Peptide‐K71 synthesis

Peptide‐K71 was chemically synthesised by a commercial company. Based on the IP‐MS results for ENO1, along with amino acid sequences and protein structure surrounding the K71 site, we identified a target peptide sequence that could inhibit lactylation at the ENO1‐K71 site (AVEHINKTIAPALVSKK). Drawing from reported sequences of cell‐penetrating peptides (CPPs) in the literature and incorporating company recommendations, we determined that the CPP sequence (YGRKKRRQRRR) needed to be added to the C‐terminus of the target sequence to facilitate cellular uptake. To enhance the detection of the peptide's cellular uptake efficiency, a FITC fluorescent modification was added at the N‐terminus. The company then conducted structural analysis and chemically synthesised the final target peptide, designated Peptide‐K71, which included the target sequence, CPP, and FITC. The control peptide sequence was identical to Peptide‐K71, except that the lysine (K) at position 71 was mutated to arginine (R).

### 4.7 In vivo administration in mice

Oxamic acid sodium (OXA, 750 mg/kg body weight), a common LDHA inhibitor, was injected intraperitoneally 3 h before the CLP or sham operation to inhibit the production of lactate. To inhibit vascular lactylation, mice were injected with C646 (30 nmol/g body weight) 3 h prior to CLP or sham surgery. Peptide‐K71 or vehicle was administered via tail vein injection (3 mg/kg body weight) 3 h before the CLP or sham operation.

### 4.8 Injection of adeno‐associated virus (AAV)

An AAV serotype with high endothelial transduction efficiency (ENT), combined with the vascular endothelial cell‐specific promoter TIE, was used to achieve targeted‐knockdown gene (*ENO1*) expression in vascular endothelial cells (AAV‐ENO1 shRNA, targeted sequence: CCCGGCTTTCAATGTGATCAA). AAV‐ENO1 shRNA was constructed by GeneChem Co., Ltd. Mice were administered 200 µL of AAV‐ENO1 shRNA via tail vein injection at a viral titer of 2.5×10^12^ v.g./mL).

### 4.9 Cell transfection

Transfection of siRNAs or siNC were performed with Lipofectamine RNAiMAX reagent and the transfection of plasmids or vector was performed with Lipofectamine 2000 reagent in accordance with the manufacturer's instructions. For detailed siRNA sequences, see Supporting Information 2: Table S1.

### 4.10 Plasmid construction

All wild type plasmids were purchased from Tsingke Biotechnology Co., Ltd. Flag‐ENO1‐K64R, Flag‐ENO1‐K71R, Flag‐ENO1‐239R were constructed by Kits in accordance with manufacturer's instructions. Plasmids related to the partial deletion of TRIM21 domain were generated by a Seamless Cloning Kit, according to the manufacturer's instructions.

### 4.11 Measurement of lactate levels

Intracellular lactate levels were determined by commercial kits. Serum was collected from the peripheral blood of experimental mice by centrifugation at 2000 rpm for 20 min; then, serum lactate levels were measured using the same kits with intracellular lactate.

### 4.12 Enzyme‐linked immunosorbent assay (ELISA)

The protein levels of IL‐6, TNF‐α and IL‐1β were measured in the serum of mice with DuoSet ELISA kits in accordance with the manufacturer's instructions. All samples were detected in duplicate.

### 4.13 Protein extraction and Western blotting

Cells or tissues were first lysed in RIPA buffer with protease and phosphatase inhibitors and then centrifuged at 16 000 × *g* at 4°C for 20 min to prepare a supernatant. Protein concentrations were determined by BCA to ensure that the same concentrations of total protein were blotted. Total protein was resolved by electrophoresis, then transferred to PVDF membranes, and incubated with primary antibodies at 4°C overnight. The next day, the cells were washed and incubated with secondary antibodies. Membranes were then visualised using a Molecular Imager (ChemiDocTM XRS+ Imaging System). Representative figures from three biological experiments are shown.

### 4.14 Immunofluorescence staining and confocal analysis

Cells were seeded onto glass coverslips for immunofluorescence staining according to the commercially available kit. Images were captured using a laser confocal microscope.

### 4.15 Immunoprecipitation (IP)

Protein extracts were incubated overnight with primary antibody using a rotary mixer at 4°C. The protein–antibody complex was then incubated with Protein A/G magnetic beads using a rotating mixer at 4°C for 2 h. Next, we added 1× SDS loading buffer to the protein–antibody–magnetic beads complex, and boiled the samples at 95°C for 10 min. The next steps were the same as the protocol used for western blotting.

### 4.16 Quantitative reverse transcription PCR (RT‐qPCR)

Total RNA was extracted from cells using Trizol Reagent. Then, cDNAs were synthesised using the PrimeScript II First Strand cDNA Synthesis Kit. Finally, cDNAs were amplified with the SYBR Premix Ex Taq RT‐PCR kit using a Roche 480 Real Time PCR System. The levels of target mRNAs were normalised to internal housekeeping controls. Expression levels (fold‐change) were calculated using the 2^−ΔΔCT^ method. The primer sequences for all genes are provided in Supporting Information 2: Table S2.

### 4.17 RNA‐seq assay and data analysis

Following stimulation with Lactate and LPS, we then extracted total RNA from ECs. Next, we used the TruSeq RNA Sample Preparation Kit (Illumina) for library preparation. Next, libraries at a final concentration of 10 pM were clustered onto a single read flow cell by cBot and sequenced to 150 bp using an Illumina NovaSeq 6000 instrument (Illumina). Library construction and sequencing were performed by oeBiotech. Co. Ltd. Differential gene expression was then analysed by the standard Illumina sequence analysis pipeline. Differentially expressed genes were selected as those with a false discovery rate < .5, a fold‐change ≥ 1.5 or ≤ .5, and a *p* value < .05.

### 4.18 RNA immunoprecipitation (RIP)

RIP was performed using the Millipore RIP Kit in accordance with the manufacturer's instructions. First, approximately 1 × 10^7^ cells were lysed using RIP lysis buffer. Then, cell lysis was co‐incubated with primary antibody or IgG antibody as a negative control at 4°C overnight. Finally, the target RNA bound to the protein was extracted, and the RNA level was detected by RT‐qPCR.

### 4.19 mRNA stability assay

ECs were treated with actinomycin D at a final concentration of 1000 ng/mL for 0, 2, 4, and 6 h. Subsequently, cells were collected and RNA was extracted for reverse transcription. The mRNA transcript levels were then detected by RT‐qPCR.

### 4.20 Vascular permeability assay

The vascular permeability assay was based on the intravenous injection of Evans Blue in mice, as described previously. In brief, all mice were administered with 200 µL of .5% Evans Blue intravenously and then sacrificed by CO_2_ inhalation 30 min later. Following dissection, we collected 50 to 200 mg of tissue from the organs of interest and places these samples into 1.5 mL tubes. First, the samples were weighed; then, we added 500 µL of formamide and incubated for 48 h at 55°C to extract Evans Blue from the tissues. Following incubation, we centrifuged the formamide/Evans Blue mixture to pellet any remaining tissue fragments, and measured absorbance at 610 nm. Then, we calculated relative permeability in the tissues of the same weight.

### 4.21 Cell proliferation assay

Cell proliferation was assayed by with the Cell‐Counting Kit 8 in accordance with the manufacturer's instructions. Cells were then seeded in 96‐well plates at a density of 5 × 10^3^ cells per well. Following stimulation, the culture media was replaced with fresh medium (100 µL per well) containing 10 µL of CCK‐8 solution at different times (0, 24, 48 h). We incubated CCK8 solution with cells in a cell incubator for 2 h and then measured absorbance at 450 nm with a multimode microplate reader.

### 4.22 Endothelial permeability assay

For endotheliam permeability assays, Fluorescein Isothiocyanate–Dextran (FITC‐dextran, FD40) was used to evaluate the permeability of ECs. Next, 200 µL of cell suspension in culture medium was inoculated into the upper chamber of a Transwell plate, and 400 µL medium was added to the lower chamber. Following stimulation, the old medium was aspirated and the upper and lower chambers were washed three times with PBS. Then, 400 µL of PBS was added to the lower chamber, and 200 µL of PBS containing FD40 was added to the upper chamber. Incubation was performed for 20 min in a cell incubator; then, we collected PBS from the lower chamber. The fluorescence intensity at 510 nm of absorbed light was determined under excitation at 480 nm.

### 4.23 Cell migration assay

Cells were cultured in six‐well plates to 80%–90% confluency. Then, we used a 200 µL sterile pipette tip to create a scratch on the cell monolayer. Then, we used a microscope to acquire immediate images of the scratches (0 h). Then, the cells were returned to the incubator. Images of cell migration were observed again 24 h later. Then, we used Image J to measure the areas occupied by scratches. Cell mobility was calculated and analysed statistically.

### 4.24 Detection of LDH, AST, and CK

The levels of LDH, AST, and CK in serum were detected by commercial kits in accordance with the manufacturer's instructions.

### 4.25 Flow cytometry

Peptide‐K71 was used to stimulate ECs and then the cells were digested with pancreatic enzymes. Subsequently, the cells were washed twice with PBS and resuspended with 300 µL of FACS (5% FBS + 95% PBS). FITC fluorescence intensity was then measured by flow cytometry (BD, Franklin Lake, NJ, USA).

### 4.26 RNA pull down

RNA pull down was performed using the Bersinbio RNA pull‐down Kit in accordance with the manufacturer's instructions.

### 4.27 Mass spectrometry

Proteins were first separated by SDS‐PAGE gels and stained with Coomassie blue. Gel lanes were then sliced into different bands. The specific analysis process was performed as the manufacturer's instructions, and binding proteins were used for lactylation identification by MS in the Institute of Biomedical Sciences, Fudan University.

### 4.28 Assessment of successful induction of sepsis in mice

As clinical diagnostic criteria cannot be directly applied to animal models, mice were considered septic when they exhibited^1^, ^17^:

- significantly elevated serum lactate levels;
- marked hypothermia (core temperature reduction >2°C–3°C from baseline);
- increased organ injury markers, including AST, LDH, and CK.

These criteria were evaluated prior to survival analysis and vascular permeability assessment to ensure model robustness and disease severity.

### 4.29 Statistical analysis

Statistical analyses were performed using GraphPad Prism 10.0. For in vitro assays, data represent at least three independent experiments; in vivo sample sizes are detailed in figure legends. Data distribution and variance homogeneity were first evaluated. To compare two groups, we used the unpaired two‐tailed Student's *t*‐test (parametric) or Mann–Whitney *U* test (non‐parametric). For multi‐group comparisons, one‐way or two‐way ANOVA was conducted, followed by Tukey's or Bonferroni post hoc tests for all‐pair or selected‐pair comparisons, respectively. Welch's correction was applied in cases of heteroscedasticity. Non‐parametric multi‐group analyses were performed using the Kruskal–Wallis test with Dunn's post hoc adjustment. Spearman's correlation was used for association analyses. Data are presented as mean ± SD, with a two‐sided *p* < .05 defining statistical significance.

The manufacturers and catalogue numbers (Cat. Nos.) of the reagents used in this ​study​ are detailed in Supporting Information 2: Table S3. The dilution ratios, product catalog numbers, and other relevant details about western blotting antibody are detailed in Supporting Information 2: Table S4. Quantification for all western blots are detailed in Supporting Information 3.

## AUTHOR CONTRIBUTIONS

**Xueru Xie**: Writing—original draft; methodology; formal analysis; data curation; conceptualisation. **Tingyan Liu**: Resources; methodology; conceptualisation. **Caiyan Zhang**: Visualisation; methodology. **Ye Cheng**: Methodology. **Yajing Gao**: Conceptualisation. **Wenfeng Xiao**: Supervision; resources. **Haiyan Guo**: Supervision; conceptualisation. **Yutong Zhou**: Methodology. **Yawei Yu**: Formal analysis. **Kexin Wang**: Data curation. **Yinghong Lin**: Visualisation; methodology. **Lisheng Xiao**: Methodology. **Yingying Zhang**: Supervision; resources. **Weiguo Yang**: Resources. **Gangfeng Yan**: Resources. **Guoping Lu**: Supervision; resources; project administration; funding acquisition. **Yufeng Zhou**: Writing—review & editing; supervision; resources; project administration; funding acquisition; conceptualisation.

## CONFLICT OF INTEREST STATEMENT

The authors declare no competing interests.

## ETHICS STATEMENT

This clinical study was approved by the Research Ethics Board of the Children's Hospital of Fudan University in Shanghai, China (approval reference number: 2023 (249)). All mice experiments were conducted in compliance with relevant laws and institutional guidelines, under the supervision of the Animal Studies Committee of the Children's Hospital of Fudan University.

## Supporting information



Supporting Figures

Supporting Tables

Supporting Information

## Data Availability

Raw data from the RNA‐seq has been deposited in the GEO database under accession number GSE281679. Additional data are available from the authors upon request.
