# Supplementary material for: Targeting the lactylation of ENO1 alleviates endothelial dysfunction in sepsis
Source: Clin Transl Med. 2026 Jan 14;16(1):e70597. doi: 10.1002/ctm2.70597 (PMC12801394; doi:10.1002/ctm2.70597)
Supplement: Supplementary file 1 — Supporting Figures [file CTM2-16-e70597-s001.docx]

**Supplementary Figure:**

**
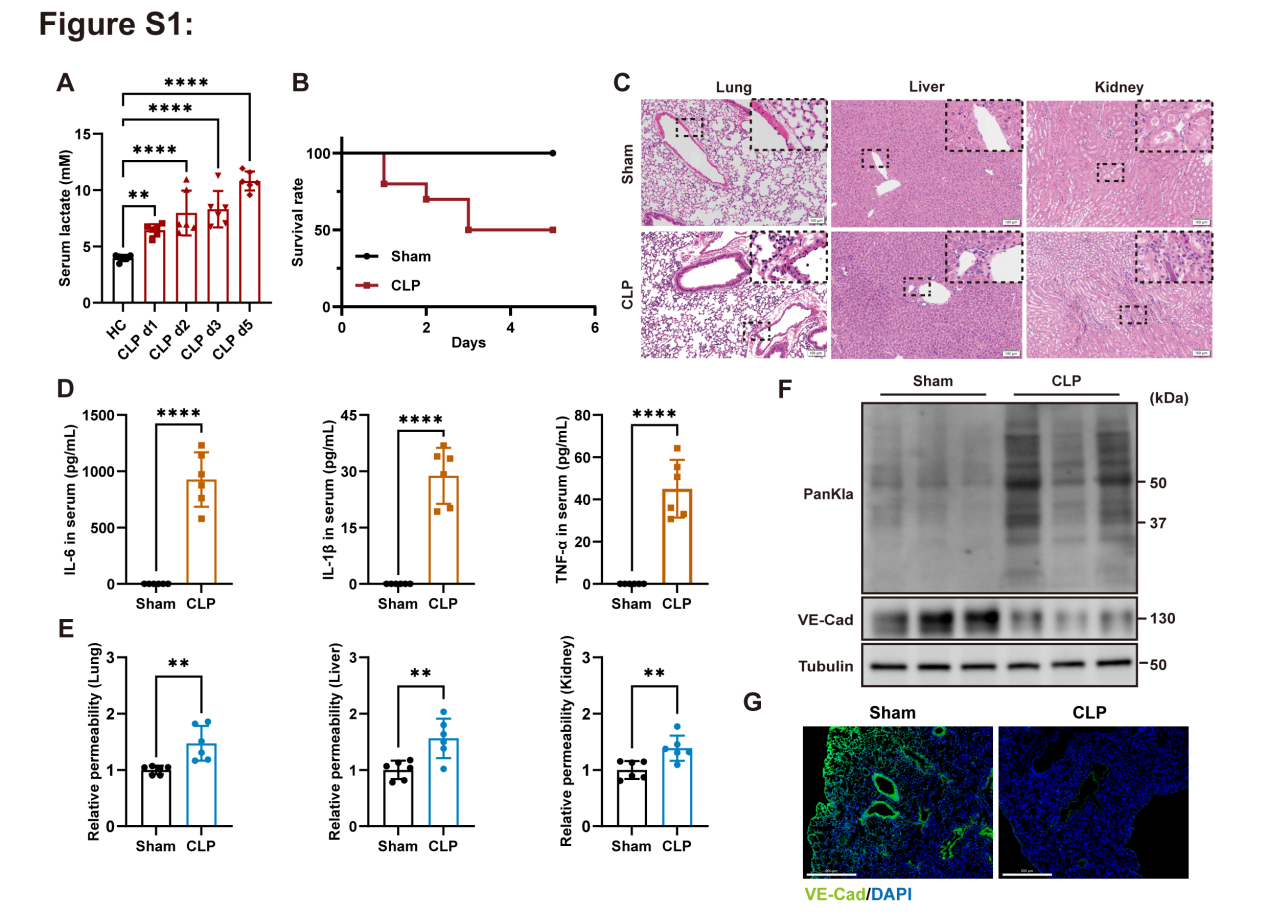
Figure S1. Elevated lactate was associated with increased vascular lactylation and vascular permeability in sepsis mice.** (A) Serum lactate levels of CLP mice at different timepoints (n=6 per group). (B) Survival rate of Sham and CLP mice (n=10 per group). (C) Representative images of H&E staining in the lungs, liver and kidneys in Sham and CLP mice (scale bars, 100 μm). The small box shows the location of zoomed image. (D) The levels of IL-6, IL-1β and TNF-α in serum from mice in the Sham and CLP groups (n=6 per group). (E) Relative permeability of microvessels in the lungs, liver and kidneys was determined by Evans Blue Dye (EBD) absorbance at 610 nm (n=6 per group). (F) Lactylation and VE-Cadherin protein levels in vascular tissues from the Sham and CLP mice were detected by western blotting (n=3 per group). (G) Immunofluorescence detection of VE‑Cadherin expression in pulmonary blood vessels of mice. Green: VE-Cad; Blue: DAPI. Scale bar, 500 μm. Data are presented as mean ± SD. ns, not significant, **P < 0.01,, ****P < 0.0001.


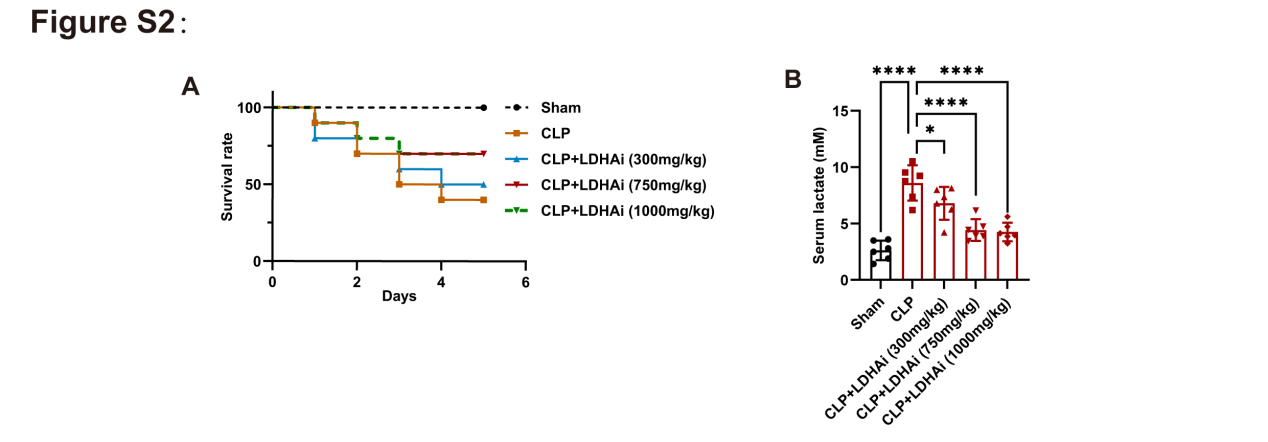


**Figure S2. Determined the optimal dose of LDHAi for efficacy.** (A) Survival rate of CLP mice under different doses of LDHAi (n=10 per group). (B) Serum lactate levels of CLP mice under different doses of LDHAi (n=6 per group).


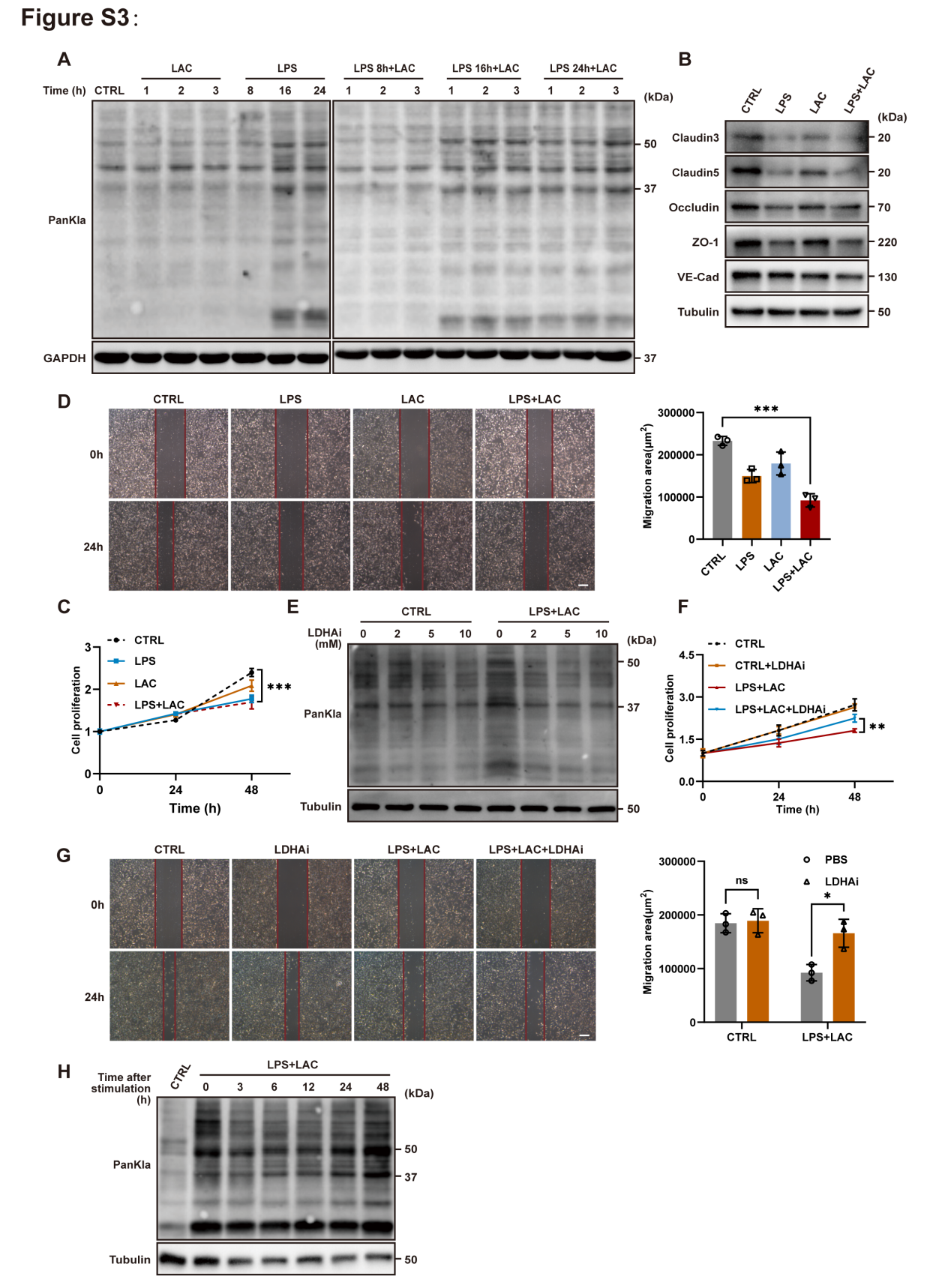


**Figure S3. ECs underwent lactylation after stimulation with LPS and lactate.** (A) Lactylation levels in HUVECs after stimulation at different times and detected by western blotting (B). Expression levels of different endothelial barrier-related proteins were detected after stimulation with LPS and lactate. (C) Cell proliferation of HUVECs was determined by CCK8 absorbance at 450 nm (n=5 per group). (D) Cell migration areas of HUVECs were measured after 24 h (on the left is the migration image, and on the right is the quantitative analysis). (E) Levels of lactylation in HUVECs stimulated in response to different concentrations of LDHAi as detected by western blotting. (F) Cell proliferation of HUVECs following LDHAi stimulation was determined by CCK8 absorbance at 450 nm (n=5 per group). (G) Cell migration areas of HUVECs were measured after 24h of LDHAi stimulation (on the left is the migration image, and on the right is the quantitative analysis). (H) Levels of lactylation in HUVECs were detected after stimulation with LPS and lactate for different times. Data are presented as mean ± SD. ns, not significant, *P < 0.05, **P < 0.01, ***P < 0.001.


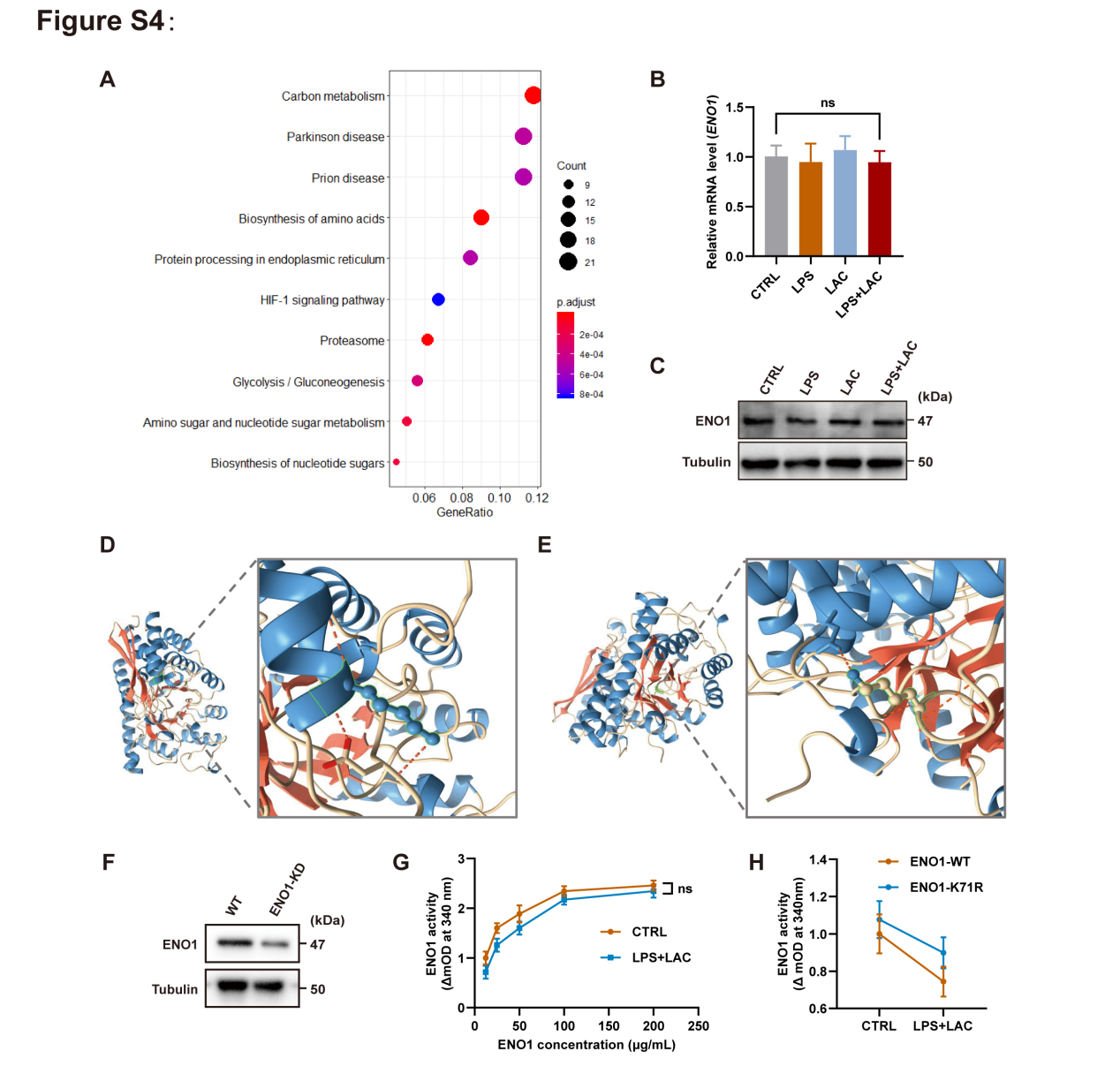


**Figure S4. Structure of different lactylated sites of ENO1 and their effects on enzyme activity.** (A) KEGG enrichment showed that glycolysis pathway was enriched in HUVECs after LPS and lactate stimulation. (B) ENO1 mRNA expression levels in HUVECs following stimulation with LPS and lactate as determined by RT-qPCR. (C) ENO1 protein levels in HUVECs after stimulation with LPS and lactate were determined by western blotting. (D) Ribbon diagram of the crystal structure of human ENO1 protein at the K64 site. (E) Ribbon diagram of the crystal structure of human ENO1 protein at the K239 site. (F) ENO1 protein levels were knocked down in EA.HY926 and confirmed by western blotting. (G) The enzyme activity of ENO1 in ECs was detected. (H) The enzyme activity of ENO1 was detected after different plasmids were overexpressed in ENO1 knockdown ECs. Data are presented as mean ± SD. ns, not significant.


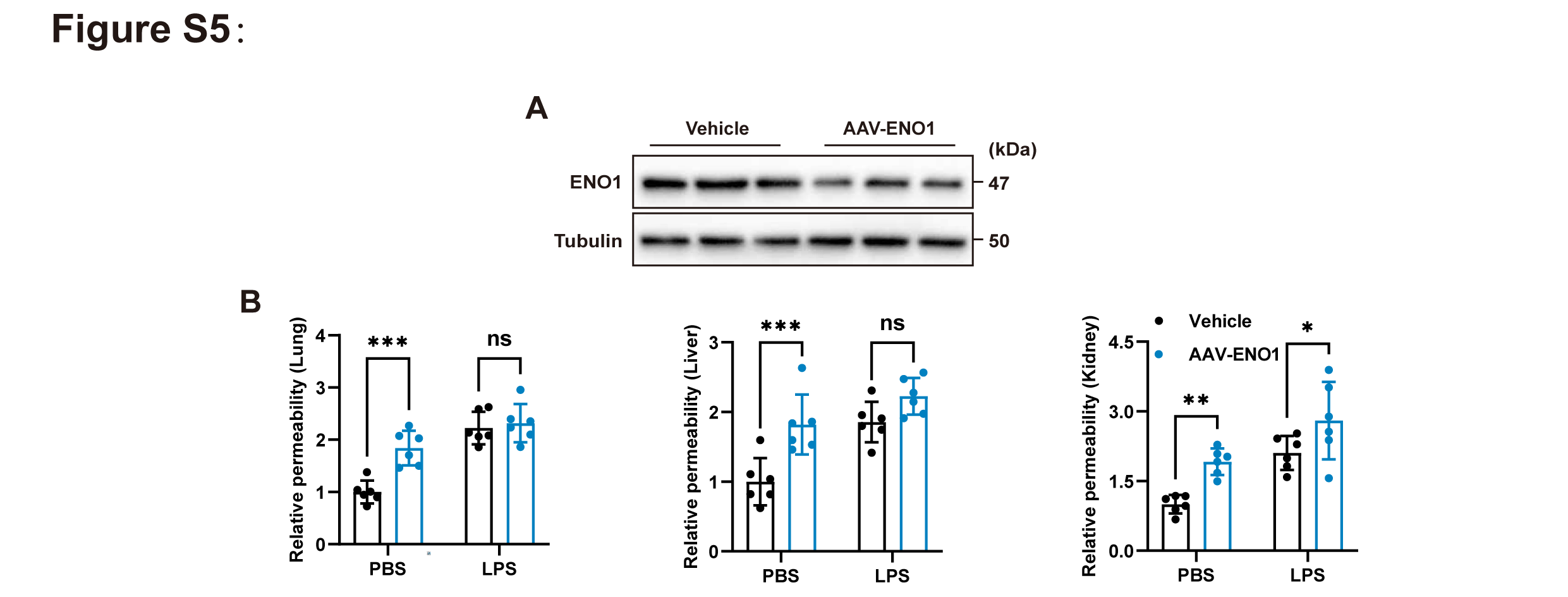


**Figure S5. Targeted deletion of ENO1 in vascular ECs led to increased vascular permeability in mice.** (A) ENO1 protein levels in blood vessels were detected by western blotting. (B) Relative permeability of microvessels in the lungs, liver and kidneys in Vehicle-treated and AAV-ENO1-treated mice following LPS administration were determined by Evans Blue Dye (EBD) absorbance at 610 nm (n = 6 per group). Data are presented as the mean ± SD. ns, not significant, *P < 0.05, ***P < 0.001.


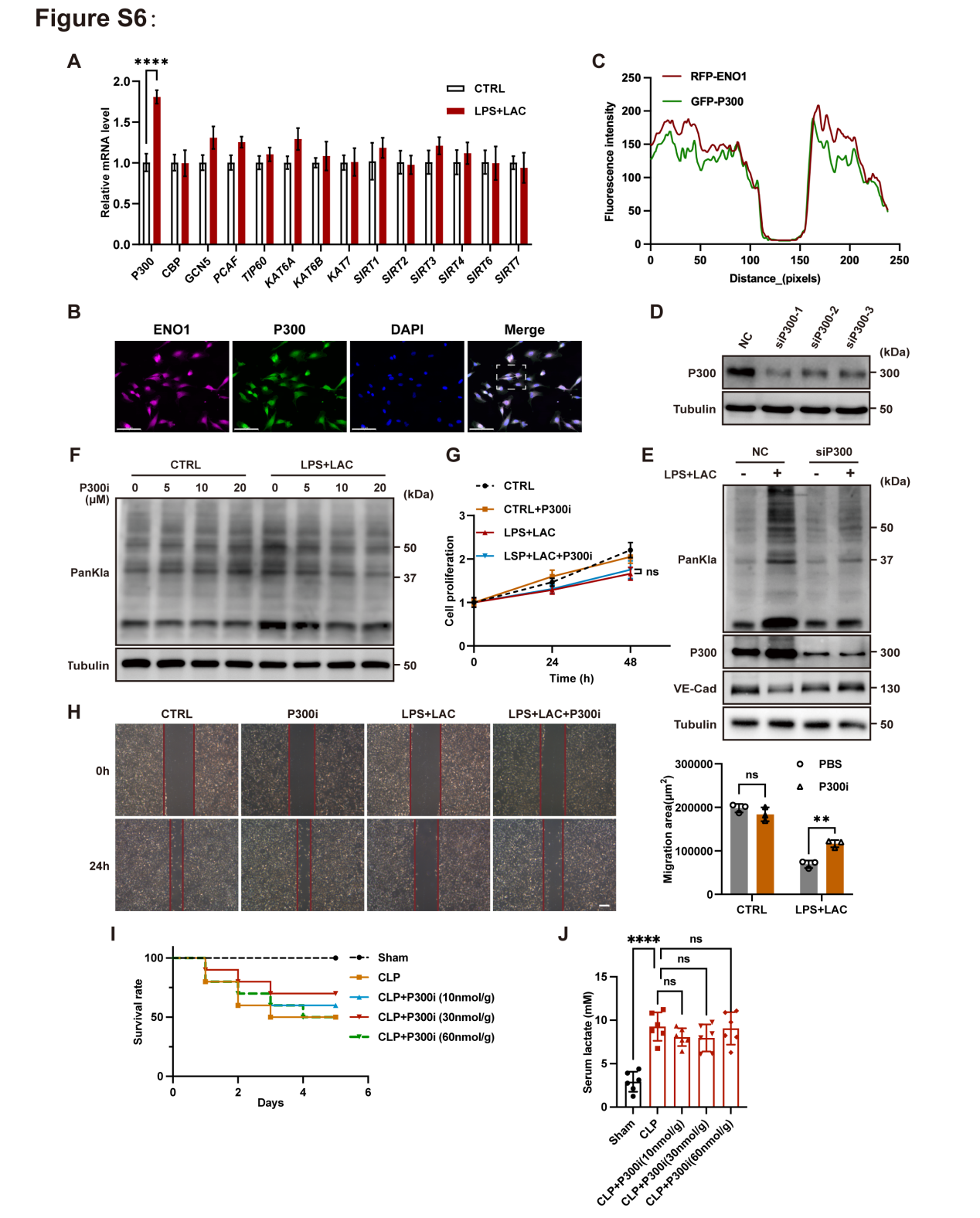


**Figure S6. A P300 inhibitor reduced endothelial permeability by reducing endothelial lactylation.** (A) The mRNA expression levels of acetyltransferases and deacetyltransferases in HUVECs as determined by RT-qPCR (n=6 per group). (B) Immunofluorescence examined the interaction between ENO1 and P300. Purple: ENO1; Green: P300; Blue: DAPI. Scale bar, 100 μm. (C) High-resolution colocalization analysis data of (B). (D) P300 protein levels after knockdown by different siRNAs as detected by western blotting. (E) Changes of lactylation and VE-Cadherin expression following the knockdown of P300. (F) Levels of lactylation in HUVECs when stimulated by different concentrations of P300i (C646) and detected by western blotting. (G) Cell proliferation following P300i treatment as determined by CCK8 absorbance at 450nm (n=5 per group). (H) Cell migration areas of HUVECs were measured after 24 h of treatment with P300i (on the left is the migration image, and on the right is the quantitative analysis). (I) Survival rate of CLP mice under different doses of P300i (n=10 per group). (J) Serum lactate levels of CLP mice under different doses of P300i (n=6 per group). Data are presented as mean ± SD. ns, not significant, **P < 0.01, ****P < 0.0001.


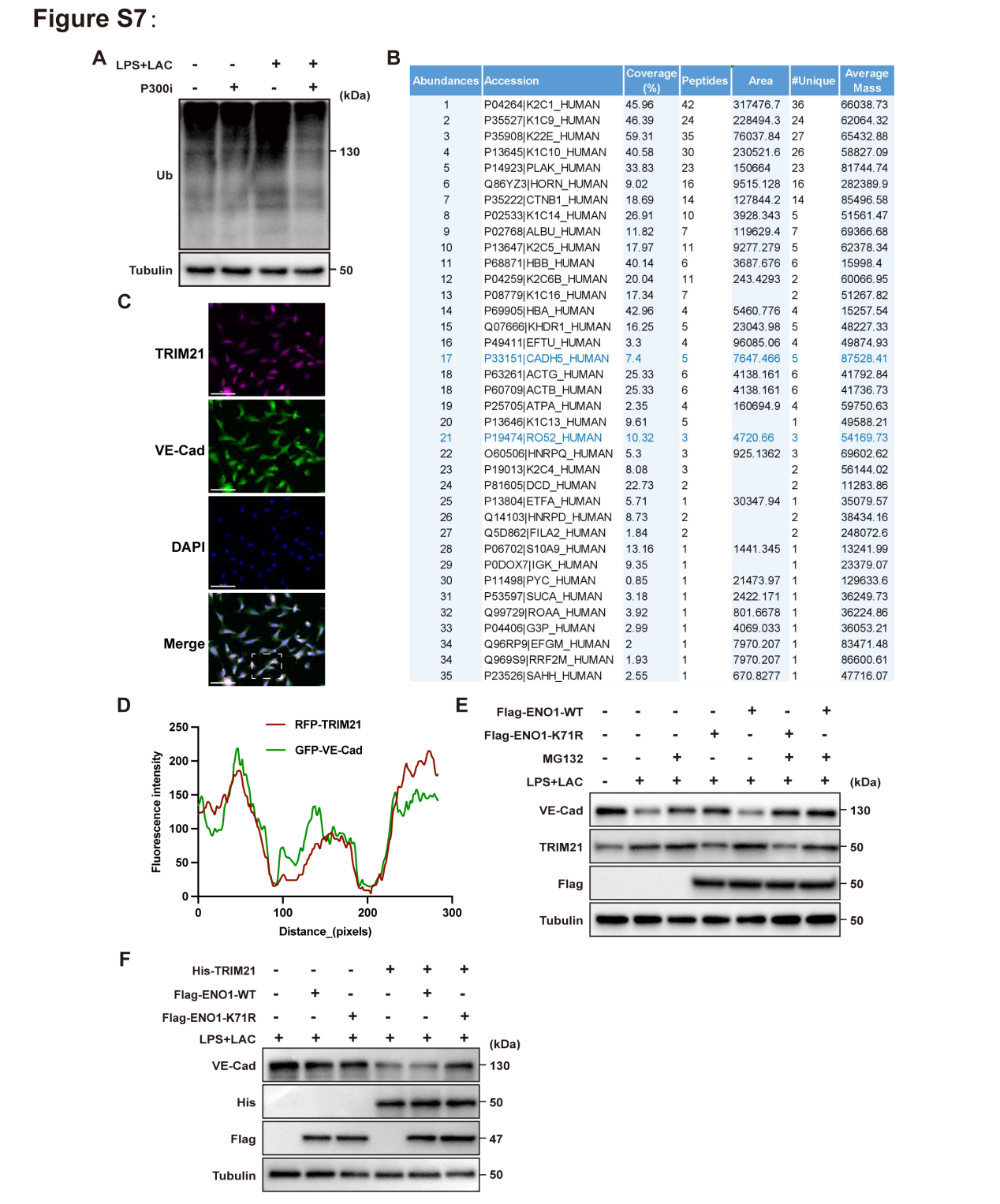


**Figure S7. TRIM21 was an E3 ubiquitin ligase that bound to VE-Cadherin.** (A) Ubiquitination levels in ECs were detected by western blotting after P300i treatment. (B) By performing immunoprecipitation of VE-Cadherin in EA.HY926 followed by mass spectrometry analysis, the table shows the proteins identified as potential binding partners of VE-Cadherin, including their abundance ranking, number of peptides, and other related parameters. (C) Immunofluorescence examined the interaction between TRIM21 and VE-Cadherin. Purple: TRIM21; Green: VE-Cadherin; Blue: DAPI. Scale bar, 100 μm. (D) High-resolution colocalization analysis data of (C). (E) TRIM21 and VE-Cadherin protein levels were detected by western blotting after combined treatment with Flag-ENO1-K71R and MG132. (F) After overexpressing His-TRIM21 in TRIM21-KO ECs and separately overexpressing ENO1-WT and ENO1-K71R, the expression of VE-Cadherin was detected by Western blotting.


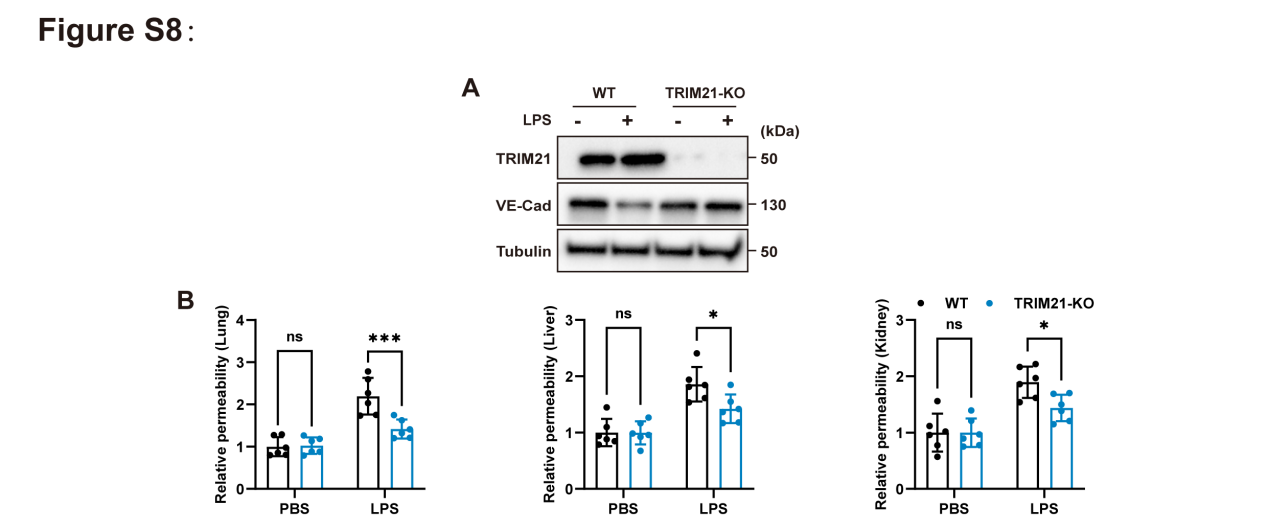


**Figure S8. Knockout of TRIM21 reduced vascular permeability in septic mice.** (A) TRIM21 and VE-Cadherin protein levels of blood vessels in WT and TRIM21-KO mice following LPS administration as detected by western blotting. (B) Relative permeability of microvessels in the lungs, liver and kidneys in WT and TRIM21-KO mice following the administration of LPS as determined by Evans Blue Dye (EBD) absorbance at 610 nm (n = 6 per group). Data are presented as mean ± SD. ns, not significant, *P < 0.05, ***P < 0.001.


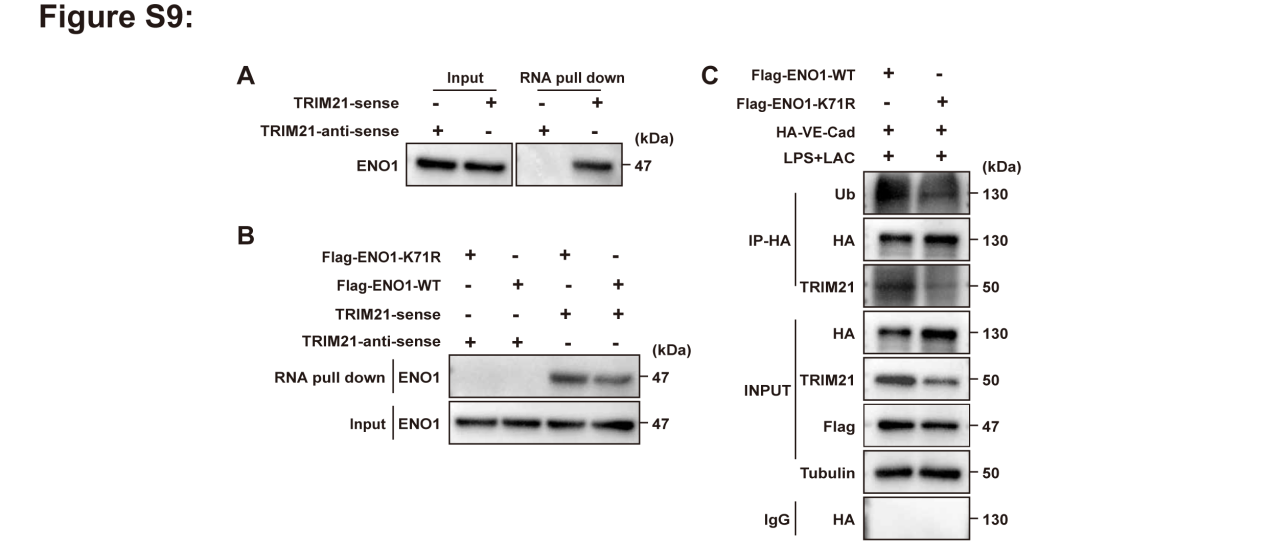


**Figure S9. TRIM21 mRNA bound to ENO1 protein and modulated the ubiquitination of VE‑Cadherin.** (A) RNA pull down assay demonstrated that TRIM21 mRNA could bind to ENO1 protein in EzCs. (B) In ENO1-KD ECs, ENO1-WT and ENO1-K71R were respectively overexpressed, and then the binding of TRIM21 mRNA to ENO1 was detected by RNA pull down. (C) ENO1-WT and ENO1-K71R were respectively overexpressed in ENO1-KD ECs, and the ubiquitination level of VE-Cadherin along with its binding to TRIM21 were detected by immunoprecipitation.


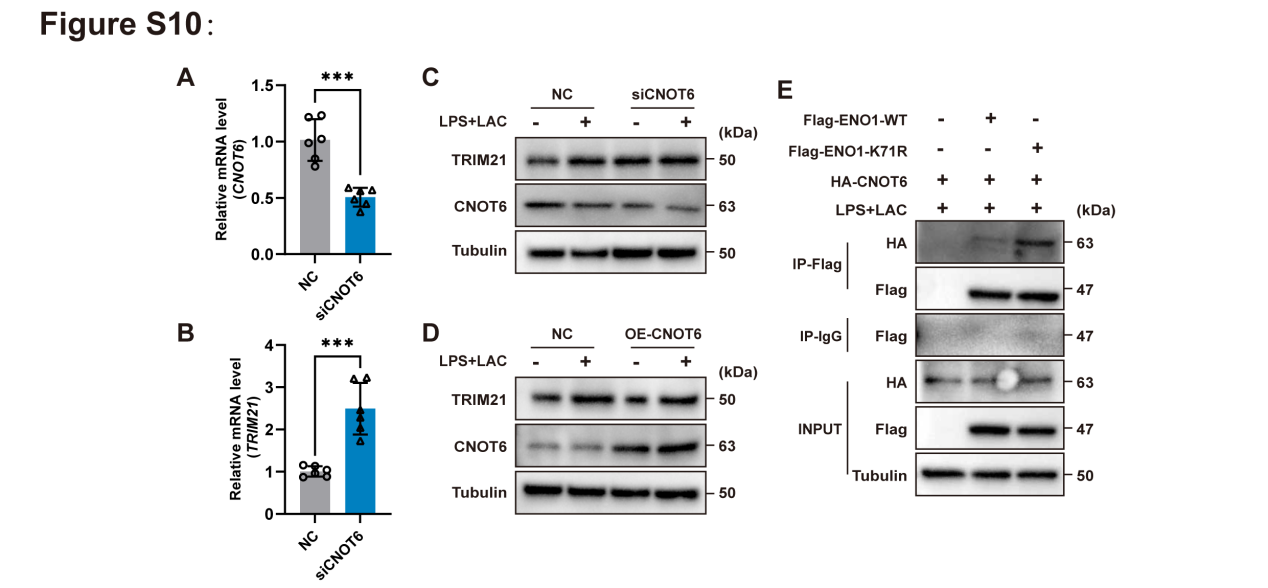


**Figure S10. CNOT6 affected the expression level of TRIM21.** (A-B) *CNOT6* and *TRIM21* mRNA expression levels after siCNOT6 transfected in EA.HY926, were determined by RT-qPCR (n=6 per group). (C-D) TRIM21 and CNOT6 protein levels were detected by western blotting after CNOT6 knockdown or overexpression. (E) Co-IP in ENO1-knockdown ECs compared the interaction between CNOT6 and ENO1-WT versus ENO1-K71R under LPS and lactate stimulation. Data are presented as the mean ± SD. ***P < 0.001.


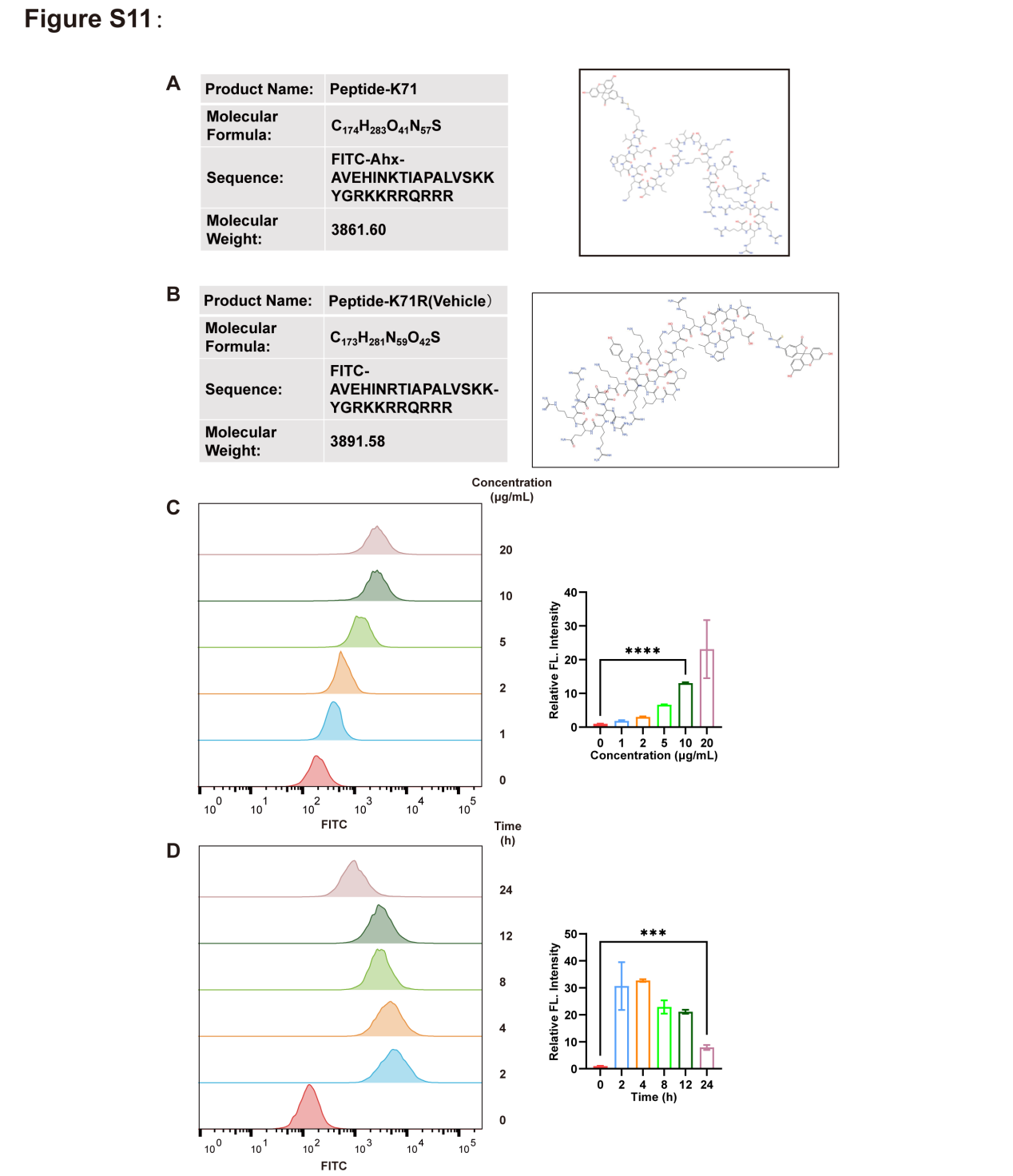


**Figure S11. Structure, concentration and treatment times with Peptide-K71.** (A) Molecular information and chemical structural formula of Peptide-K71. (B) Molecular information and chemical structural formula of Peptide-K71R (as a negative control). (C) Fluorescence intensity of HUVECs when stimulated by Peptide-K71 at different concentrations and detected by flow cytometry. Quantitative analysis of fluorescence intensity is shown in the right. (D). Fluorescence intensity of ECs when stimulated by Peptide-K71 for different times and detected by flow cytometry. Quantitative analysis of fluorescence intensity is shown on the right. Data are presented as mean ± SD. ***P < 0.001, ****P < 0.0001.


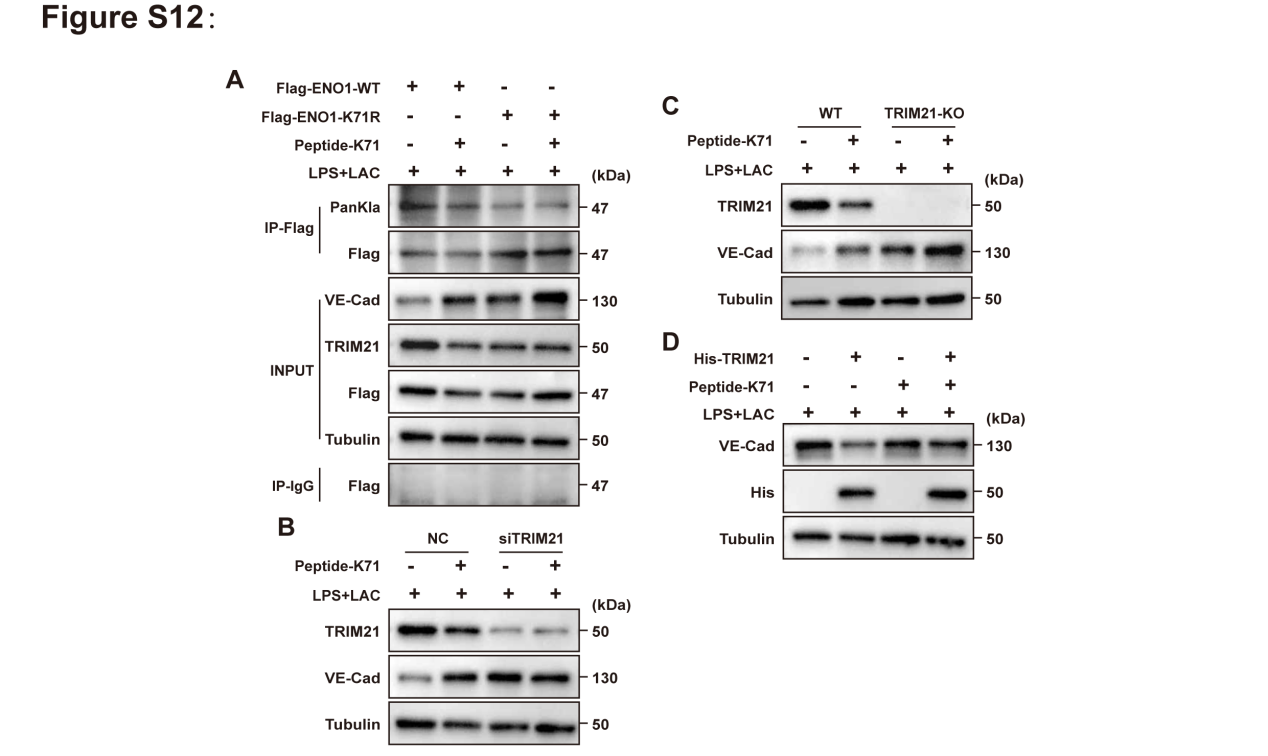


**Figure S12. Peptide‑K71 showed specificity in suppressing ENO1 lactylation and elevating VE‑Cadherin protein levels.** (A) ENO1-WT and ENO1-K71R were respectively overexpressed in ENO1-KD ECs, and then the lactylation level of ENO1 and the expression levels of TRIM21 and VE-Cadherin were detected in the presence or absence of Peptide-K71. (B) Western blotting detected the effect of Peptide-K71 on VE-Cadherin expression in ECs following siRNA-mediated knockdown of TRIM21. (C) Western blotting detected the effect of Peptide-K71 on VE-Cadherin expression in TRIM21-KO mice of sepsis. (D) After reconstituting TRIM21-KO ECs with His-TRIM21 and administering Peptide-K71, the expression of VE-Cadherin was detected by Western blotting.


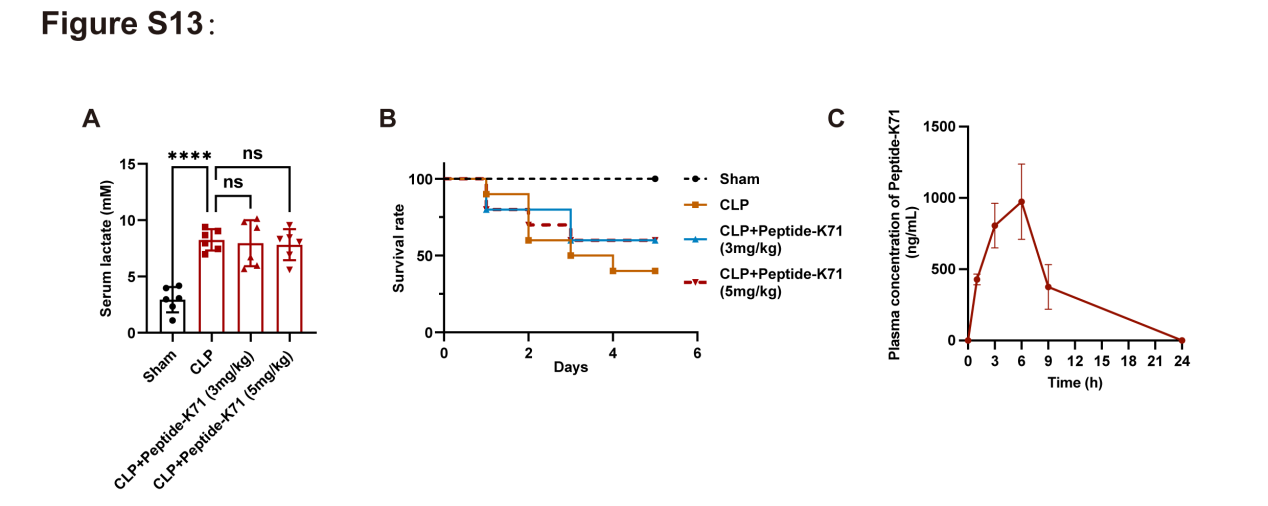


**Figure S13. Evaluated the optimal dose and pharmacokinetics of Peptide‑K71.** (A) Serum lactate of CLP mice under different doses of Peptide-K71 (n=6 per group). (B) Survival rate of CLP mice under different doses of Peptide-K71 (n=10 per group). (C) At different time points after tail vein injection of Peptide-K71(1h、3h、6h、9h、24h) , the plasma concentration of Peptide-K71 was detected by LC-MS/MS (n=3 per group). Data are presented as mean ± SD. ns, not significant, ****P < 0.0001.


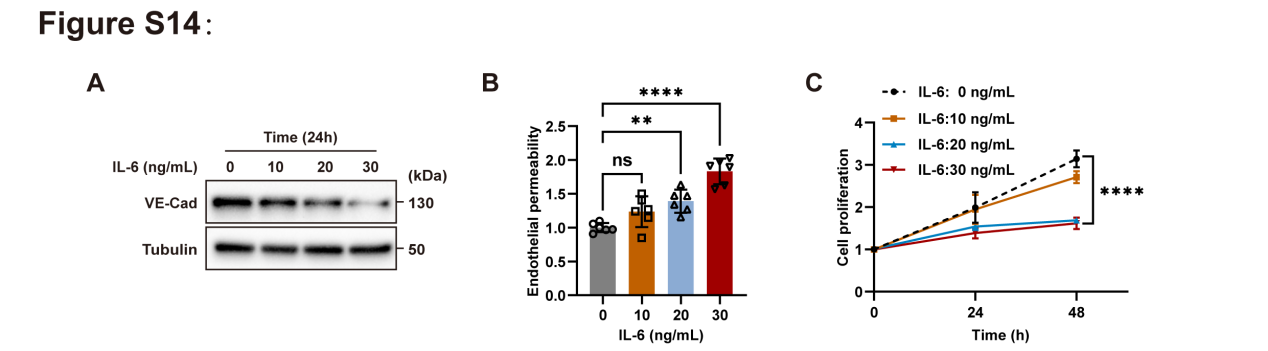


**Figure S14. IL-6 induced vascular endothelial dysfunction in sepsis.** (A) VE-Cadherin protein levels in HUVECs after stimulation with different concentration of IL-6 were detected by western blotting. (B) Endothelial permeability of HUVECs was detected by Transwell assays (n=6 per group).（C）Cell proliferation after IL-6 treatment was determined by CCK8 absorbance at 450nm (n=3 per group). Data are presented as the mean ± SD. ns, not significant, **P < 0.01, ****P < 0.0001.

Singer, M., et al. (2016). "The Third International Consensus Definitions for Sepsis and Septic Shock (Sepsis-3)." Jama 315(8): 801-810.

IMPORTANCE: Definitions of sepsis and septic shock were last revised in 2001. Considerable advances have since been made into the pathobiology (changes in organ function, morphology, cell biology, biochemistry, immunology, and circulation), management, and epidemiology of sepsis, suggesting the need for reexamination. OBJECTIVE: To evaluate and, as needed, update definitions for sepsis and septic shock. PROCESS: A task force (n = 19) with expertise in sepsis pathobiology, clinical trials, and epidemiology was convened by the Society of Critical Care Medicine and the European Society of Intensive Care Medicine. Definitions and clinical criteria were generated through meetings, Delphi processes, analysis of electronic health record databases, and voting, followed by circulation to international professional societies, requesting peer review and endorsement (by 31 societies listed in the Acknowledgment). KEY FINDINGS FROM EVIDENCE SYNTHESIS: Limitations of previous definitions included an excessive focus on inflammation, the misleading model that sepsis follows a continuum through severe sepsis to shock, and inadequate specificity and sensitivity of the systemic inflammatory response syndrome (SIRS) criteria. Multiple definitions and terminologies are currently in use for sepsis, septic shock, and organ dysfunction, leading to discrepancies in reported incidence and observed mortality. The task force concluded the term severe sepsis was redundant. RECOMMENDATIONS: Sepsis should be defined as life-threatening organ dysfunction caused by a dysregulated host response to infection. For clinical operationalization, organ dysfunction can be represented by an increase in the Sequential [Sepsis-related] Organ Failure Assessment (SOFA) score of 2 points or more, which is associated with an in-hospital mortality greater than 10%. Septic shock should be defined as a subset of sepsis in which particularly profound circulatory, cellular, and metabolic abnormalities are associated with a greater risk of mortality than with sepsis alone. Patients with septic shock can be clinically identified by a vasopressor requirement to maintain a mean arterial pressure of 65 mm Hg or greater and serum lactate level greater than 2 mmol/L (>18 mg/dL) in the absence of hypovolemia. This combination is associated with hospital mortality rates greater than 40%. In out-of-hospital, emergency department, or general hospital ward settings, adult patients with suspected infection can be rapidly identified as being more likely to have poor outcomes typical of sepsis if they have at least 2 of the following clinical criteria that together constitute a new bedside clinical score termed quickSOFA (qSOFA): respiratory rate of 22/min or greater, altered mentation, or systolic blood pressure of 100 mm Hg or less. CONCLUSIONS AND RELEVANCE: These updated definitions and clinical criteria should replace previous definitions, offer greater consistency for epidemiologic studies and clinical trials, and facilitate earlier recognition and more timely management of patients with sepsis or at risk of developing sepsis.

1. Singer, M., et al., The Third International Consensus Definitions for Sepsis and Septic Shock (Sepsis-3). Jama, 2016. 315(8): p. 801-10.

1. Singer, M., et al., The Third International Consensus Definitions for Sepsis and Septic Shock (Sepsis-3). Jama, 2016. 315(8): p. 801-10.

2. Certo, M., et al., Lactate modulation of immune responses in inflammatory versus tumour microenvironments. Nat Rev Immunol, 2021. 21(3): p. 151-161.

1. Singer, M., et al., The Third International Consensus Definitions for Sepsis and Septic Shock (Sepsis-3). Jama, 2016. 315(8): p. 801-10.

2. Certo, M., et al., Lactate modulation of immune responses in inflammatory versus tumour microenvironments. Nat Rev Immunol, 2021. 21(3): p. 151-161.

3. Cecconi, M., et al., Sepsis and septic shock. Lancet, 2018. 392(10141): p. 75-87.

1. Singer, M., et al., The Third International Consensus Definitions for Sepsis and Septic Shock (Sepsis-3). Jama, 2016. 315(8): p. 801-10.

2. Certo, M., et al., Lactate modulation of immune responses in inflammatory versus tumour microenvironments. Nat Rev Immunol, 2021. 21(3): p. 151-161.

4. Chen, Y., et al., Metabolic regulation of homologous recombination repair by MRE11 lactylation. Cell, 2024. 187(2): p. 294-311.e21.
