# Supplementary material for: Targeting the lactylation of ENO1 alleviates endothelial dysfunction in sepsis
Source: Clin Transl Med. 2026 Jan 14;16(1):e70597. doi: 10.1002/ctm2.70597 (PMC12801394; doi:10.1002/ctm2.70597)
Supplement: Supplementary file 2 — Supporting Tables [file CTM2-16-e70597-s002.pdf]

Table S1: The list of siRNA sequences (Homo)

Gene	Sense	Anti-sense
siLDHA-1	CATGTACGTTGCTATCCAGGC	CTCCTTAATGTCACGCACGAT
siLDHA-2	CCTGCCCTGGTTAGCAAGAA	GGCGTTCGCACCAAACTTAG
siLDHA-3	CCCACAGGCACGATCTGTT	GTTGGGCAGGGTTAGCACC
siENO1-1	CTGCACAGGAGTACCAGGAGA	GCACAAACTCTGCGTGAATCC
siENO1-2	TGCCAAACCAGATGATGCCT	ATAGCCCATAGGCGGGTTGA
siENO1-3	CTCAGAGCCAGTTTCTGCCA	AGGCACCTGTCCCTGTGA
siP300-1	CTAGGGGTCTTCTCGGCTTG	GATACGTGGTCAGCCAAGGG
siP300-2	CCCGAATCGCCGTGAAGAAA	TTTACAAGACTCCTCGGCCTTG
siP300-3	GTCGCGCTGTCTCCCC	TCATCCAGACGTTTGTTGAAGTC
siTRIM21-1	CCAGAATGTTTGCGCTGGAC	GACACAGACTTTCCCACACTGA
siTRIM21-2	AAAGGGCAGCGAGCGATT	AGGAAAGACTGCATTCGGGT
siTRIM21-3	TCGGCAGACTCGTTCATCTG	TTCCAGTTGGAGTTCGAGGC
siCNOT6-1	GGAGGAUGUAUACAAUUAUTT	AUAAUUGUAUACAUCCUCCTT
siCNOT6-2	GGACCUGUCAUCUAAUAAATT	UUUAUUAGAUGACAGGUCCTT
SiCNOT6-3	CCUCCAAGGUCUUGGAUUATT	UAAUCCAAGACCUUGGAGGTT

Table S2: Primer sequences for RT-qPCR (Homo)

Gene	Forward primer	Reverse primer
ACTB	CATGTACGTTGCTATCCAGGC	CTCCTTAATGTCACGCACGAT
ENO1	CCTGCCCTGGTTAGCAAGAA	GGCGTTCGCACCAAACTTAG
CDH5	CCCACAGGCACGATCTGTT	GTTGGGCAGGGTTAGCACC
TRIM21	CTGCACAGGAGTACCAGGAGA	GCACAAACTCTGCGTGAATCC
P300	TGCCAAACCAGATGATGCCT	ATAGCCCATAGGCGGGTTGA
CBP	CTCAGAGCCAGTTTCTGCCA	AGGCACCTGTCCCTGTGA
GCN5	CTAGGGGTCTTCTCGGCTTG	GATACGTGGTCAGCCAAGGG
PCAF	CCCGAATCGCCGTGAAGAAA	TTTACAAGACTCCTCGGCCTTG
TIP60	GTCGCGCTGTCTCCCC	TCATCCAGACGTTTGTTGAAGTC
KAT6A	CCAGAATGTTTGCGCTGGAC	GACACAGACTTTCCCACACTGA
KAT6B	AAAGGGCAGCGAGCGATT	AGGAAAGACTGCATTCGGGT
KAT7	TCGGCAGACTCGTTCATCTG	TTCCAGTTGGAGTTCGAGGC
SIRT1	AGGCCACGGATAGGTCCATA	GTGGAGGTATTGTTTCCGGC
SIRT2	CCCTCTCACCCTCTGGAGAC	TTCCGCAGGAAGTCCATGTC
SIRT3	GGTAGTTGAACGGGTCGAGG	CACTCACATCGTCCCTGCC
SIRT4	GGGGTGTGAAGTGTCCGTAG	ACCCAATGGAGGCTTTCGAG
SIRT6	GGAAGGTGTGGGAACTGGC	CGTGGGGACCCCTGAAGTC
SIRT7	ACTTGGTCGTCTACACAGGC	CTCAGGTCGGCAGCACTAAC
CNOT6	ACAGAACAACCACCTCCAAGG	TGTAGTCCCAGTTTAGCGCC

Table S3: **KEY RESOURCES TABLE**

Chemicals		
LAC (L-lactate)	Absin, Shanghai,China	abs42020226
LDHAi (Oxamic acid sodium)	MedChemexpress, New Jersey, NJ, USA	HY-W013032A
Tribromoethanol	Nanjing Aibei Biotechnology, Nanjing, China	Cat#M2920
Evans Blue	Sangon Biotech, Shanghai, China	A602025
Formamide	Sangon Biotech, Shanghai, China	A600212-0500
DAPI	Wuhan Servicebio Technology, Wuhan, China	G1012
LPS (LPS from Escherichia coli O55:B5)	Sigma, Milpitas, CA, USA	L2880
FD40	Sigma, Milpitas, CA, USA	60842-46-8
Coomassie blue	ShareBio, Shanghai, China	SB-WB207
P300i (C646)	MCE	HY-13823
Trizol	Invitrogen, Carlsbad, CA, USA	15596018CN
Cycloheximide (CHX)	MCE	HY-12320
MG132	MCE	HY-13259
Lipofectamine2000	Invitrogen, Carlsbad, CA, USA	11668-019
Lipofectamine RNAiMAX	Invitrogen, Carlsbad, CA, USA	13778-150
RIPA Buffer	Thermo Fisher Scientific, Waltham, MA, USA	89901
HaltTM Protease & Phosphatase Inhibitor Cocktail（100x）	Thermo Fisher Scientific, Waltham, MA, USA	1861281
Anti-HA Magnetic Beads	Beyotime, Shanghai, China	P2121
Anti-Flag Magnetic Beads	Beyotime, Shanghai, China	P2115
Anti-His Magnetic Beads	Beyotime, Shanghai, China	P2135
Mouse IgG Magnetic Beads	Beyotime, Shanghai, China	P2171
Rabbit IgG Magnetic Beads	Beyotime, Shanghai, China	P2173
Actinomycin D (ActD)	Cell Signaling Technology, Boston, MA，USA	15021S
Peptide-K71	ComBiosz, Suzhou, China	N/A
Peptide-K71R (Vehicle)	ComBiosz, Suzhou, China	N/A
FBS	Gibco, Carlsbad，CA，USA	10091148
DMEM	Gibco, Carlsbad，CA，USA	10-013-CVR
Critical commercial assays		
Plasmid Extraction Kit	Vazyme, Nanjing, China	DC203-01

Mut Express II Fast Mutagenesis Kit V2	Vazyme, Nanjing, China	C214-02
Seamless Cloning Kit	ShareBio, Shanghai, China	SB-HM001
Lactate Assay Kit	Sigma, Milpitas, CA, USA	MAK064
Mouse IL-6 ELISA Kit	Invitrogen, Carlsbad, CA, USA	85-88-7064-86
Mouse IL-1β ELISA Kit	Invitrogen, Carlsbad, CA, USA	88-7013A
Mouse TNF-α ELISA Kit	Invitrogen, Carlsbad, CA, USA	88-7324
Lactate dehydrogenase (LDH) Assay Kit	Nanjing Jiancheng Bioengineering Institute, Nanjing, China	A020-1-2
Aspartate aminotransferase (AST) Assay Kit	Nanjing Jiancheng Bioengineering Institute, Nanjing, China	C010-2-1
Creatine kinase (CK) Assay Kit	Nanjing Jiancheng Bioengineering Institute, Nanjing, China	A032-1-1
CCK-8 Cell Counting Kit	Vazyme, Nanjing, China	A311-01
PrimeScript RT Reagent Kit	Takara, Tokyo, Japan	RR037A
SYBR Premix Ex Taq RT-PCR Kit	Takara, Tokyo, Japan	RR420A
Magna RIP™ RNA-Binding Protein Immunoprecipitation Kit	Millipore, Billerica, MA, Miltenyi, Germany	17-700
ENO1 Enzyme Assay Kit	Abcam, Cambridge, UK	ab117994
Fluorescent Immunohistochemistry Staining Kit	Absin, Shanghai,China	abs996
Oligonucleotides		
siRNA sequences	This paper (see Table S1)	N/A
Primer sequences for RT-qPCR	This paper (see Table S2)	N/A

Table S4: Antibody-Related Information

Antibodies	Reagent Manufacturer	Product catalog numbers	Dilution ratios
GAPDH	Proteintech, Wuhan, China	60004-1-Ig	1:3000
β-Tubulin	Abcam, Cambridge, UK	ab6046	1:3000
PanKla	PTM Biolab, Hangzhou, China	PTM-1401RM	1:1000
VE-Cad	Cell Signaling Technology, Boston, MA, USA	2500S	1:1000
ENO1	Abcam, Cambridge, UK	ab155102	1:2000
LDHA	Cell Signaling Technology, Boston, MA, USA	2012S	1:1000
P300	Cell Signaling Technology, Boston, MA, USA	70088S	1:1000
TRIM21	Cell Signaling Technology, Boston, MA, USA	92043S	1:1000
Ub	Cell Signaling Technology, Boston, MA, USA	43124S	1:1000
CD31	Abcam, Cambridge, UK	ab9498	1:1000
Claudin3	Proteintech, Wuhan, China	16456-1-AP	1:1000
Claudin5	Proteintech, Wuhan, China	29767-1-AP	1:1000
Occludin	Proteintech, Wuhan, China	27260-1-AP	1:1000
ZO-1	Proteintech, Wuhan, China	21773-1-AP	1:1000
CNOT6	Proteintech, Wuhan, China	17935-1-AP	1:1000
IgG	Cell Signaling Technology, Boston, MA, USA	2729S	1:1000
Flag (Rabbit)	Proteintech, Wuhan, China	20543-1-AP	1:1000
Flag (Mouse)	Proteintech, Wuhan, China	66008-4-Ig	1:1000
His	Proteintech, Wuhan, China	66005-1-Ig	1:1000
HA	Proteintech, Wuhan, China	51064-2-AP	1:1000
