# Supplementary material for: Targeting the lactylation of ENO1 alleviates endothelial dysfunction in sepsis
Source: Clin Transl Med. 2026 Jan 14;16(1):e70597. doi: 10.1002/ctm2.70597 (PMC12801394; doi:10.1002/ctm2.70597)
Supplement: Supplementary file 3 — Supporting Information [file CTM2-16-e70597-s003.docx]

**
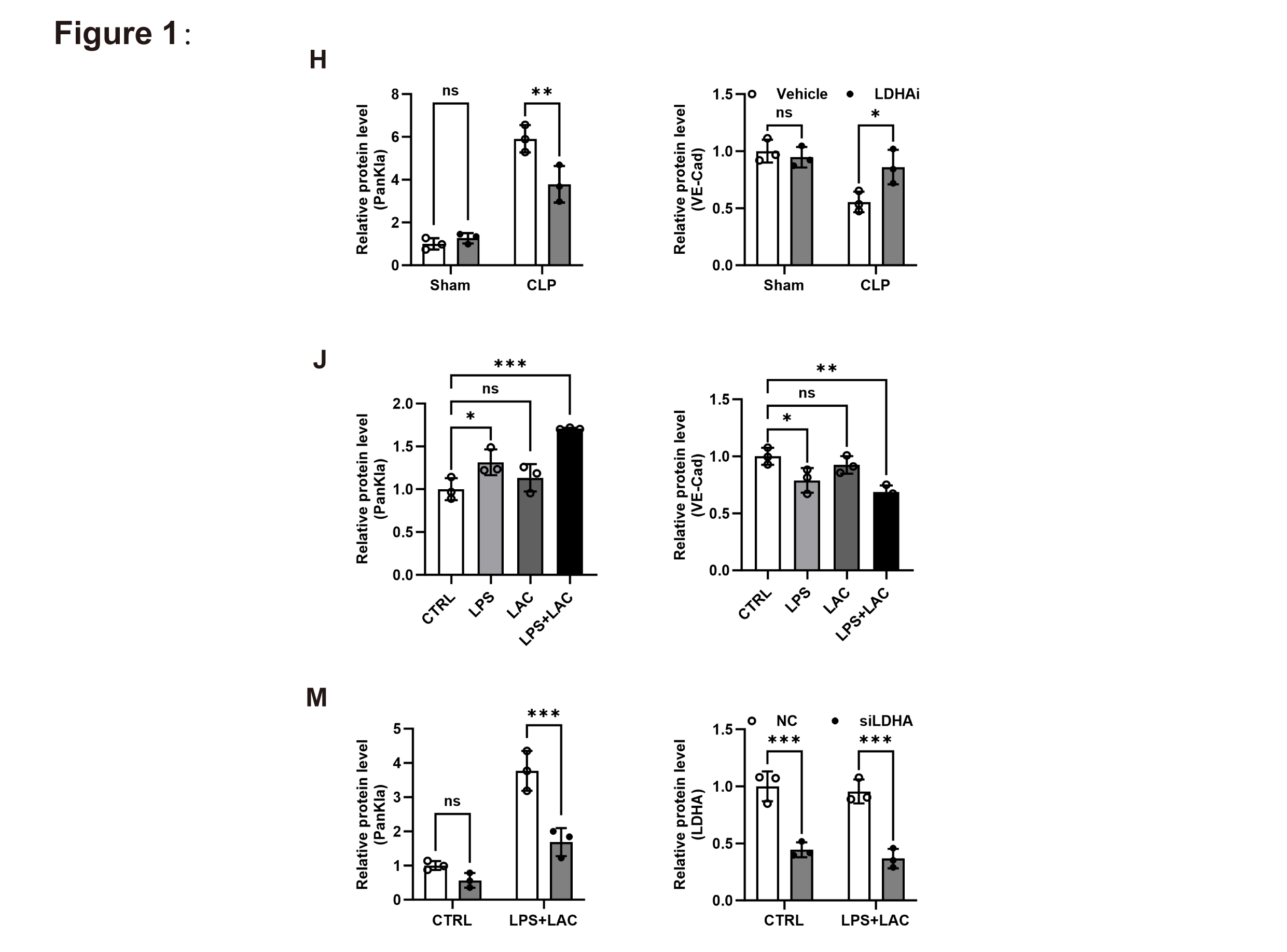
**

**Figure 1.** **Quantification of western blots in Figure 1.** (H) Relative protein level of PanKla and VE-Cadherin in Figure 1H (n=3 per group). (J) Relative protein level of PanKla and VE-Cadherin in Figure 1J (n=3 per group). (M) Relative protein level of PanKla and LAHA in Figure 1M (n=3 per group).. Data are presented as the mean ± SD. ns, not significant, *P < 0.05, **P < 0.01, ***P < 0.001.


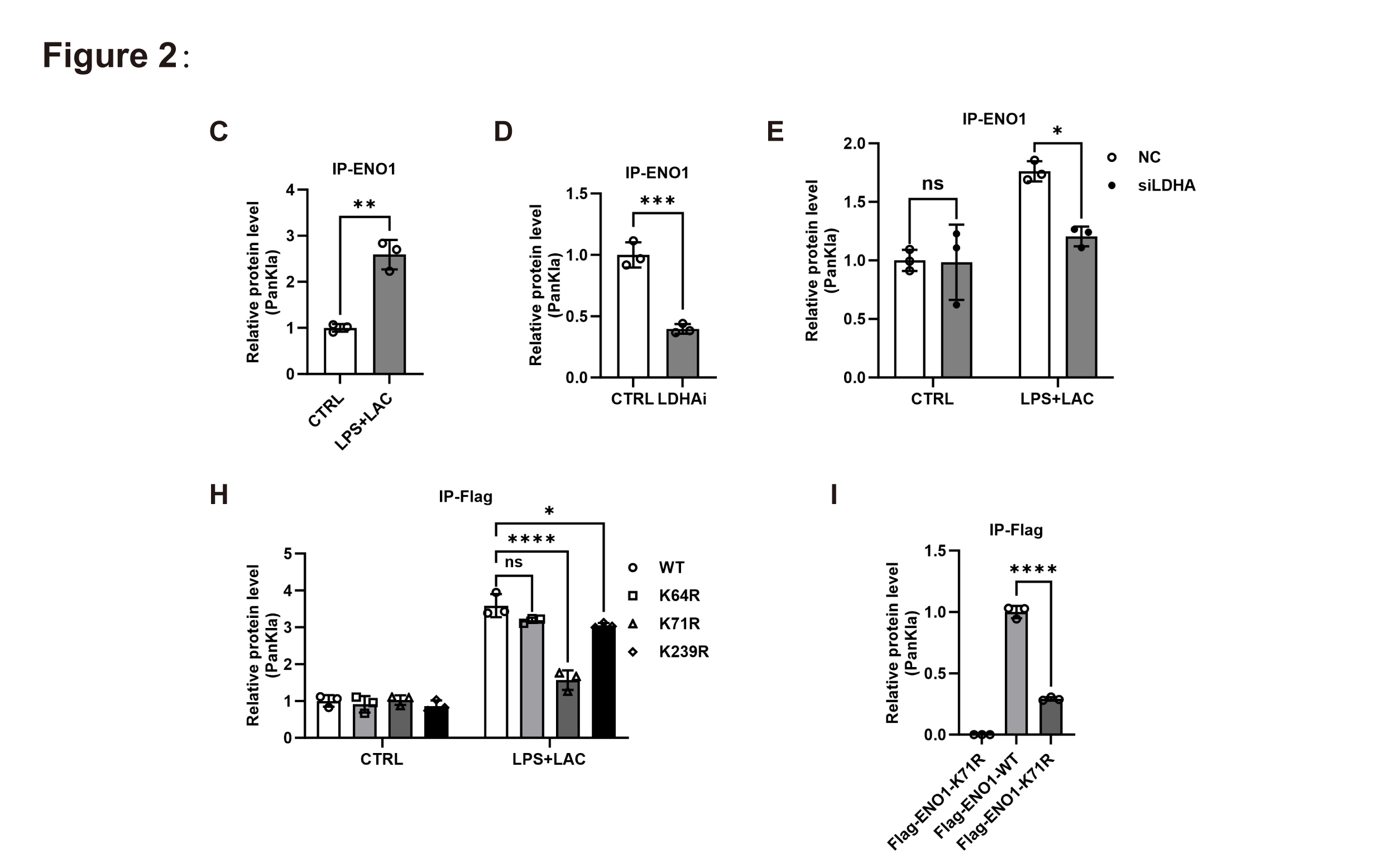


**Figure 2. Quantification of western blots in Figure 2.** (C) Relative protein level of PanKla in IP group in Figure 2C (n=3 per group). (D) Relative protein level of PanKla in IP group in Figure 2D (n=3 per group). (E) Relative protein level of PanKla in IP group in Figure 2E (n=3 per group). (H) Relative protein level of PanKla in IP group in Figure 2H (n=3 per group). (I) Relative protein level of PanKla in IP group in Figure 2I (n=3 per group). Data are presented as the mean ± SD. ns, not significant, *P < 0.05, **P < 0.01, ***P < 0.001, ****P < 0.0001.


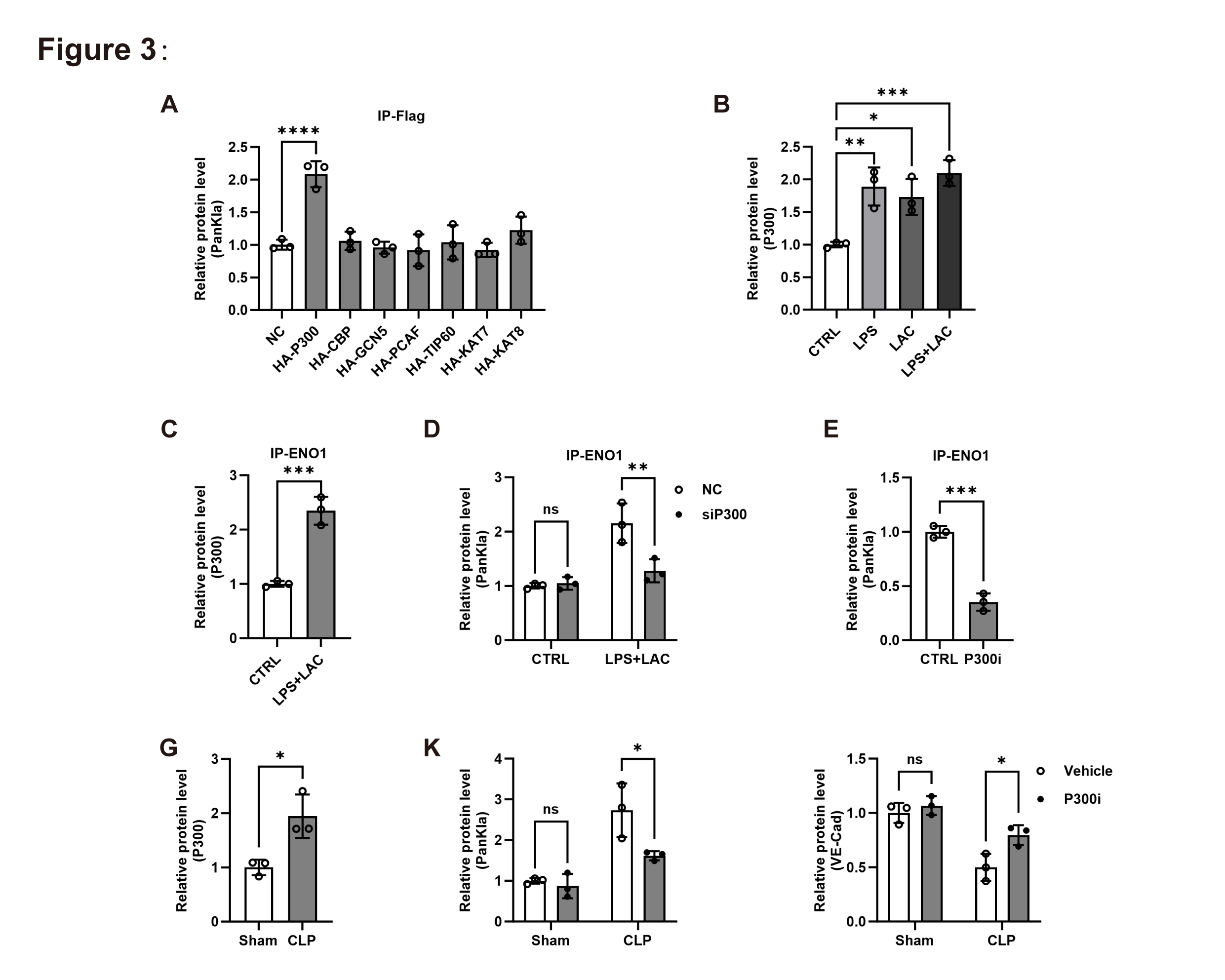


**Figure 3. Quantification of western blots in Figure 3.** (A) Relative protein level of PanKla in IP group in Figure 3A (n=3 per group). (B) Relative protein level of P300 in Figure 3B (n=3 per group). (C) Relative protein level of P300 in IP group in Figure 3C (n=3 per group). (D) Relative protein level of PanKla in IP group in Figure 3D (n=3 per group). (E) Relative protein level of PanKla in IP group in Figure 3E (n=3 per group). (G) Relative protein level of P300 in Figure 3G (n=3 per group). (K) Relative protein level of PanKla and VE-Cadherin in Figure 3K (n=3 per group). Data are presented as mean ± SD. ns, not significant, *P < 0.05, **P < 0.01, ***P < 0.001,****P < 0.0001.


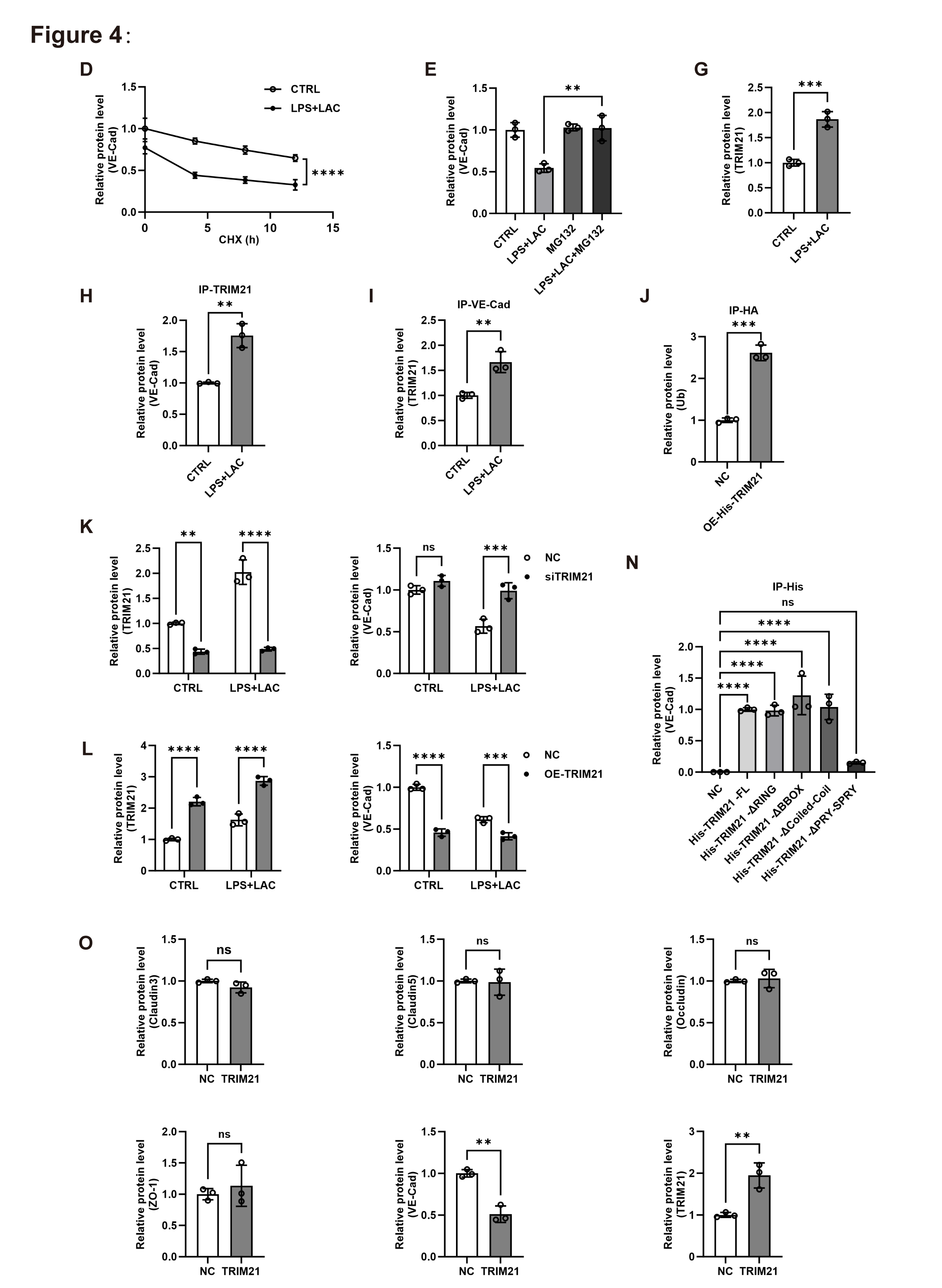


**Figure 4. Quantification of western blots in Figure 4.** (D) Relative protein level of VE-Cadherin in Figure 4D (n=3 per group). (E) Relative protein level of VE-Cadherin in Figure 4E (n=3 per group). (G) Relative protein level of TRIM21 in Figure 4G (n=3 per group). (H) Relative protein level of VE-Cadherin in IP group in Figure 4H (n=3 per group). (I) Relative protein level of TRIM21 in IP group in Figure 4I (n=3 per group). (J) Relative protein level of Ub in IP group in Figure 4J (n=3 per group). (L) Relative protein level of VE-Cadherin in IP group in Figure 4L (n=3 per group). (M) Relative protein level of TRIM21 and VE-Cadherin in Figure 4M (n=3 per group). (N) Relative protein level of TRIM21 and VE-Cadherin in Figure 4N (n=3 per group). (O) Relative protein level of TRIM21、VE-Cadherin 、Claudin3、Claudin5、Occludin and ZO-1 in Figure 4O (n=3 per group). Data are presented as mean ± SD. ns, not significant, *P < 0.05, **P < 0.01, ***P < 0.001,****P < 0.0001.


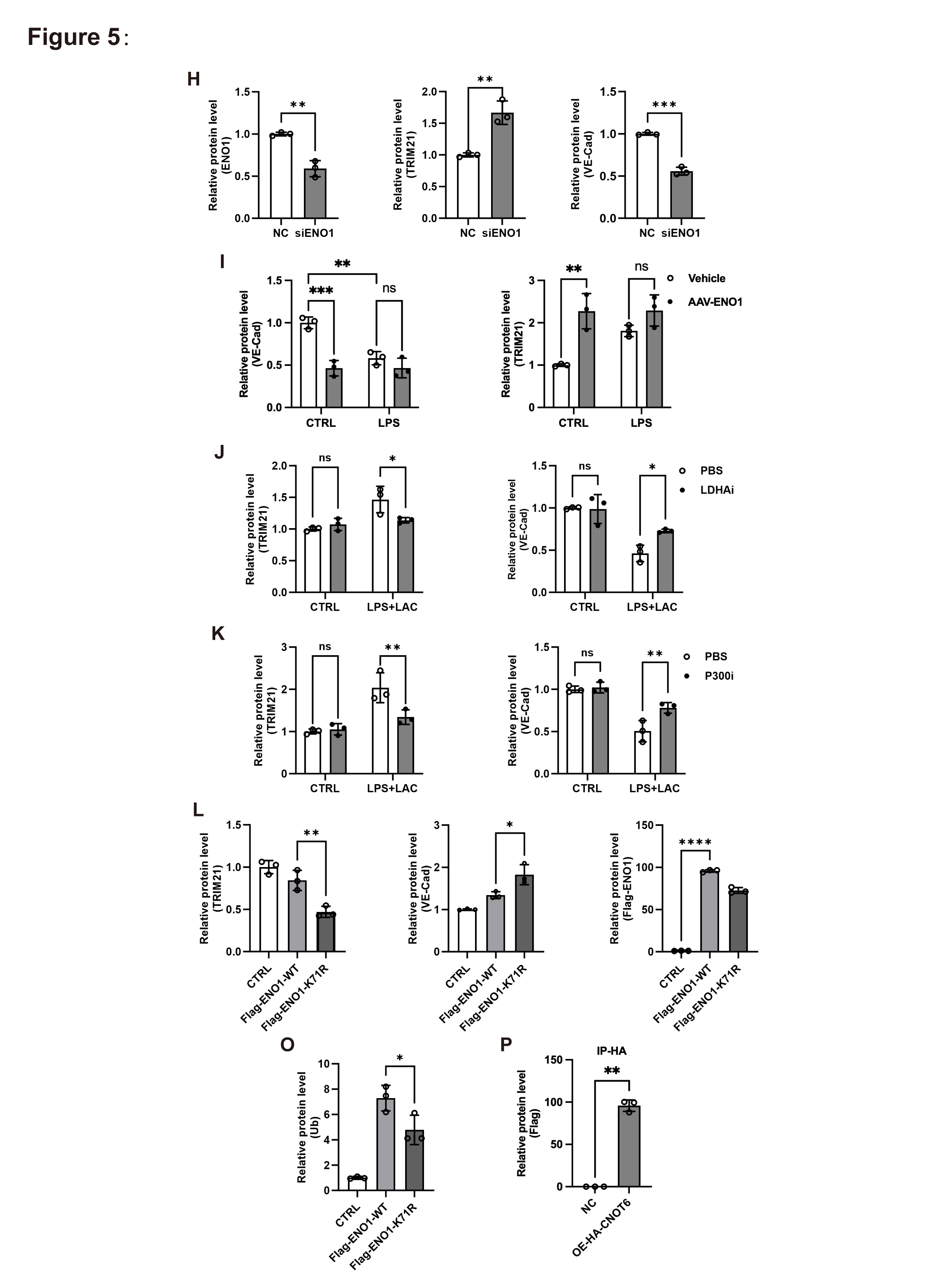


**Figure 5. Quantification of western blots in Figure 5.** (H) Relative protein level of TRIM21、VE-Cadherin and ENO1 in Figure 5H (n=3 per group). (I) Relative protein level of TRIM21 and VE-Cadherin in Figure 5I (n=3 per group). (J) Relative protein level of TRIM21 and VE-Cadherin in Figure 5J (n=3 per group). (K) Relative protein level of TRIM21 and VE-Cadherin in Figure 5K (n=3 per group). (L) Relative protein level of TRIM21、VE-Cadherin and Flag-ENO1 in Figure 5L (n=3 per group). (O) Relative protein level of Ub in IP group in Figure 5O (n=3 per group). (P) Relative protein level of Flag in IP group in Figure 5P (n=3 per group). Data are presented as mean ± SD. ns, not significant, *P < 0.05, **P < 0.01, ***P < 0.001,****P < 0.0001.


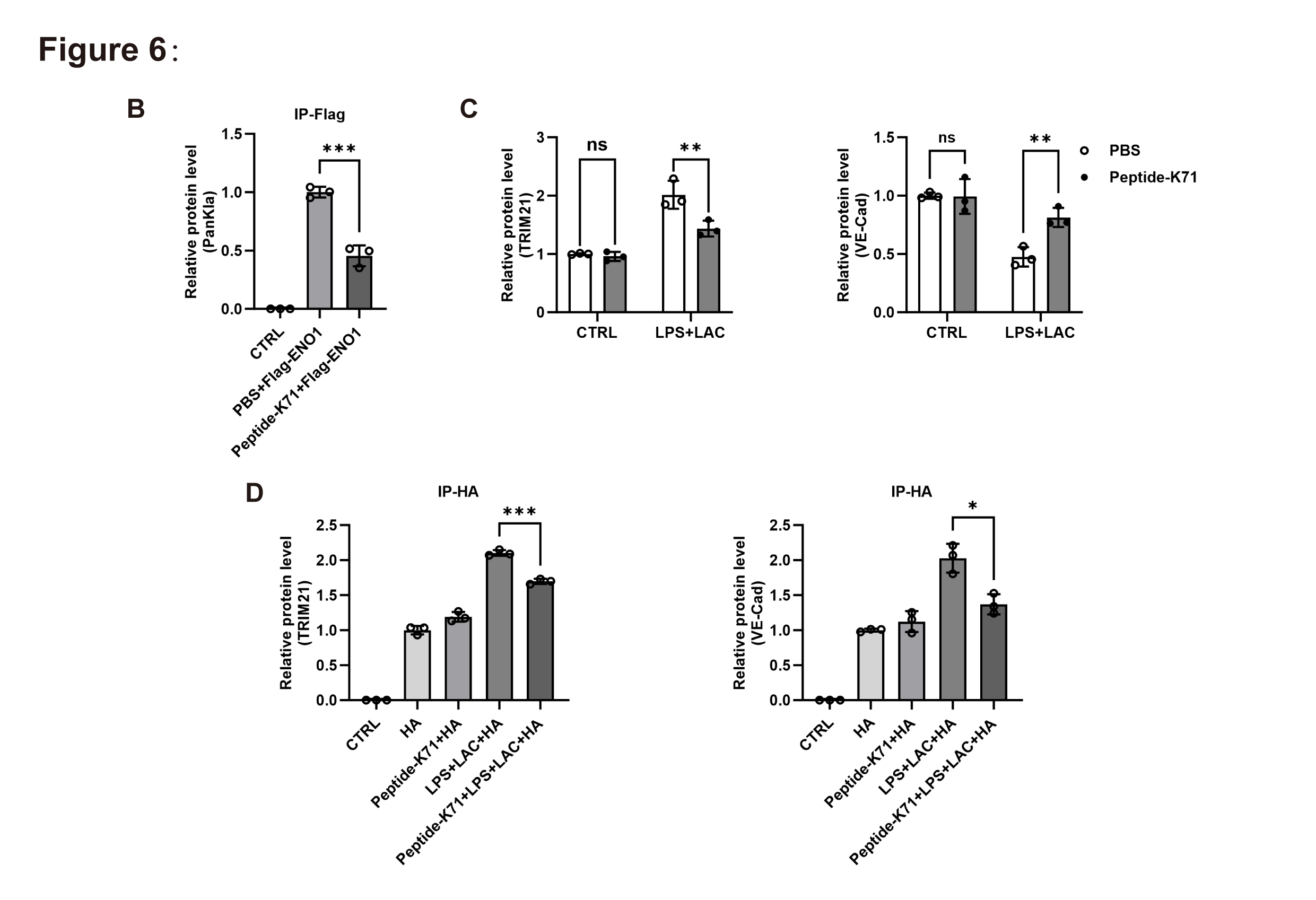


**Figure 6. Quantification of western blots in Figure 5.**  (B)Relative protein level of PanKla in IP group in Figure 6B (n=3 per group). (C) Relative protein level of TRIM21 and VE-Cadherin in IP group in Figure 6C (n=3 per group). (D) Relative protein level of TRIM21 and VE-Cadherin in IP group in Figure 6D (n=3 per group). Data are presented as mean ± SD. ns, not significant, *P < 0.05, **P < 0.01, ***P < 0.001.


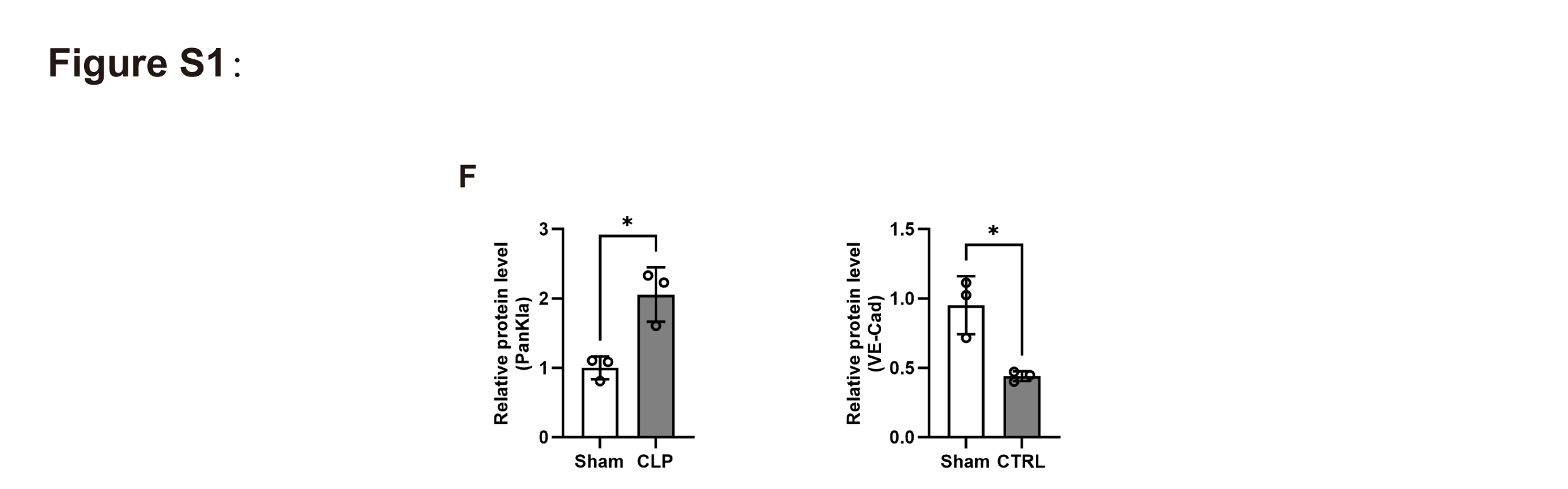


**Figure S1. Quantification of western blots in Figure S1.** (F) Relative protein level of PanKla and VE-Cadherin in Figure S1F (n=3 per group). Data are presented as mean ± SD. *P < 0.05.


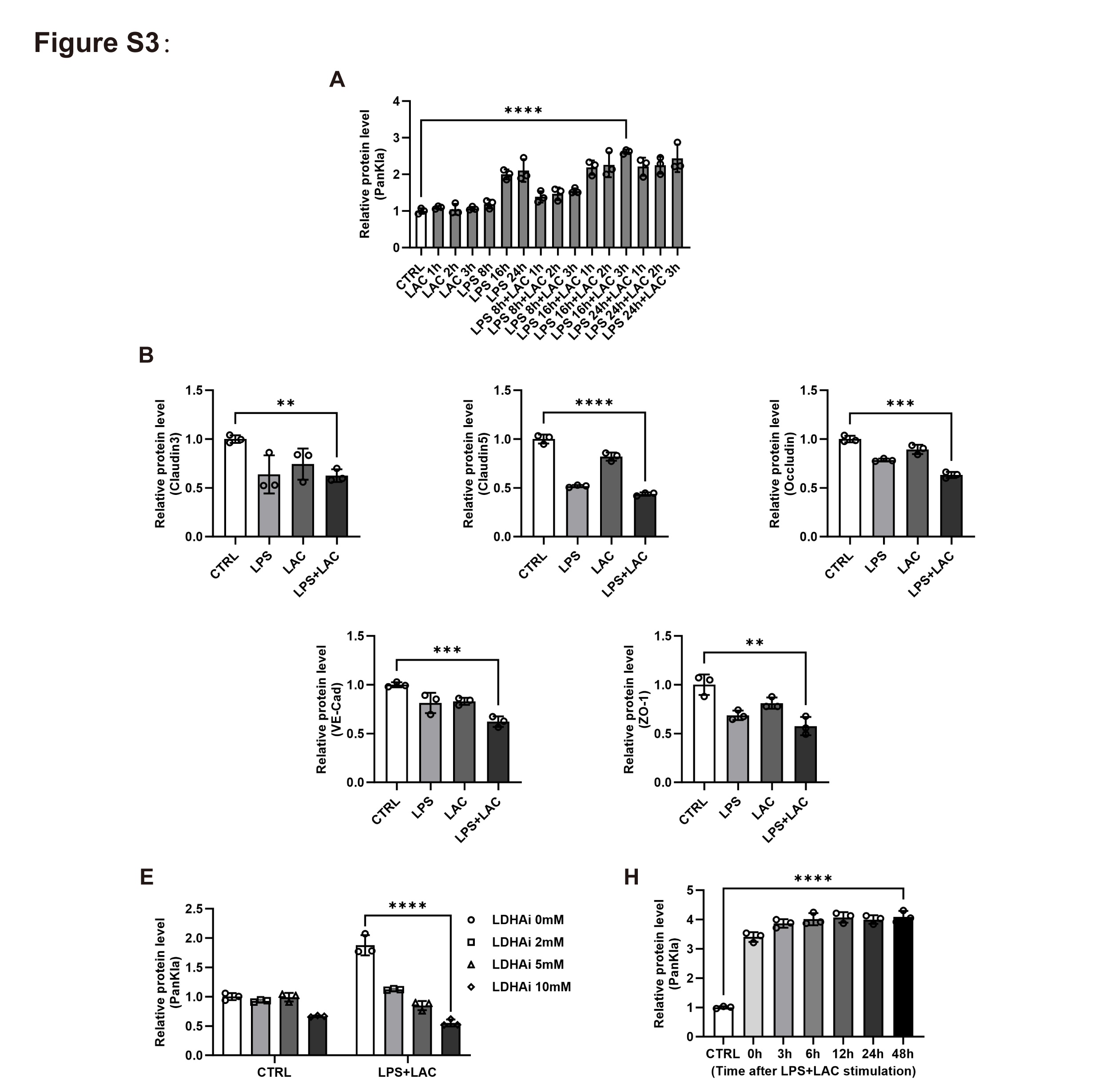


**Figure S3. Quantification of western blots in Figure S3.** (A) Relative protein level of PanKla in Figure S3A (n=3 per group). (B) Relative protein level of VE-Cadherin 、Claudin3、Claudin5、Occludin and ZO-1 in Figure S3B (n=3 per group). (E) Relative protein level of PanKla in Figure S3E (n=3 per group). (H) Relative protein level of PanKla in Figure S3H (n=3 per group). Data are presented as mean ± SD. **P < 0.01, ***P < 0.001,****P < 0.0001.


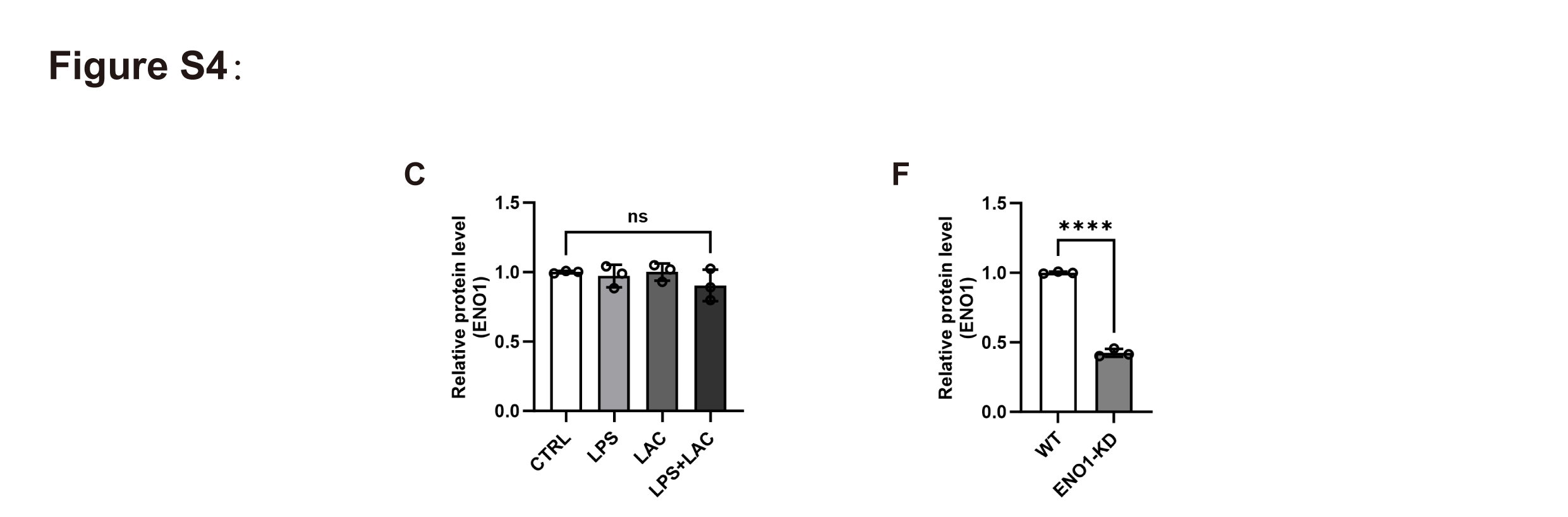


**Figure S4. Quantification of western blots in Figure S4.** (C) Relative protein level of ENO1 in Figure S4C (n=3 per group). (F) Relative protein level of ENO1 in Figure S4F (n=3 per group). Data are presented as mean ± SD. ns, not significant, ***P < 0.001.


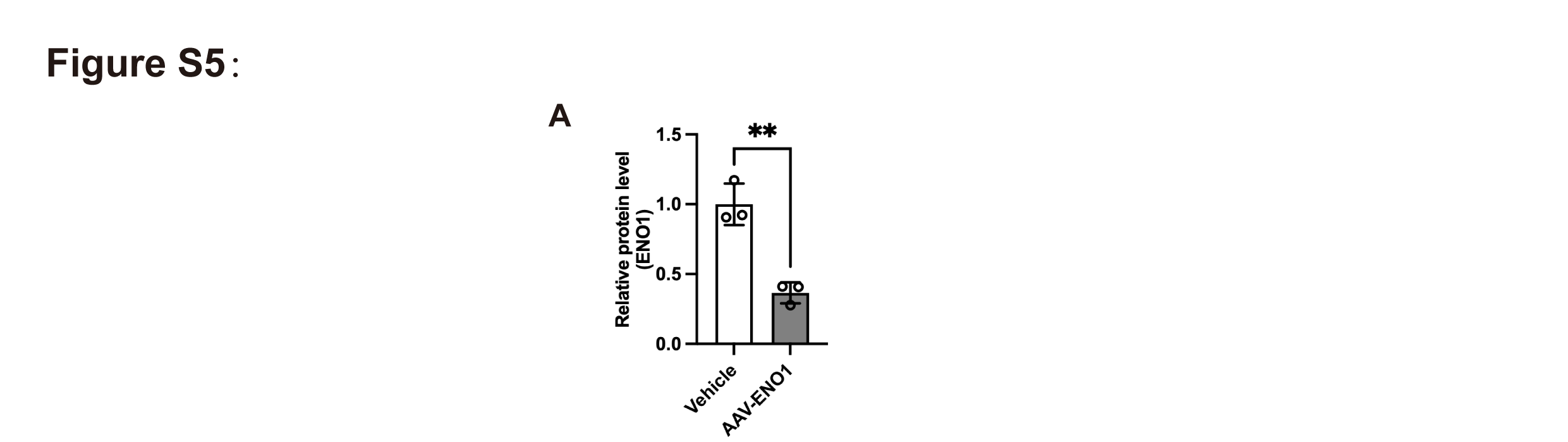


**Figure S5. Quantification of western blots in Figure S5.** (A) Relative protein level of ENO1 in Figure S5A (n=3 per group). Data are presented as the mean ± SD. **P < 0.01.


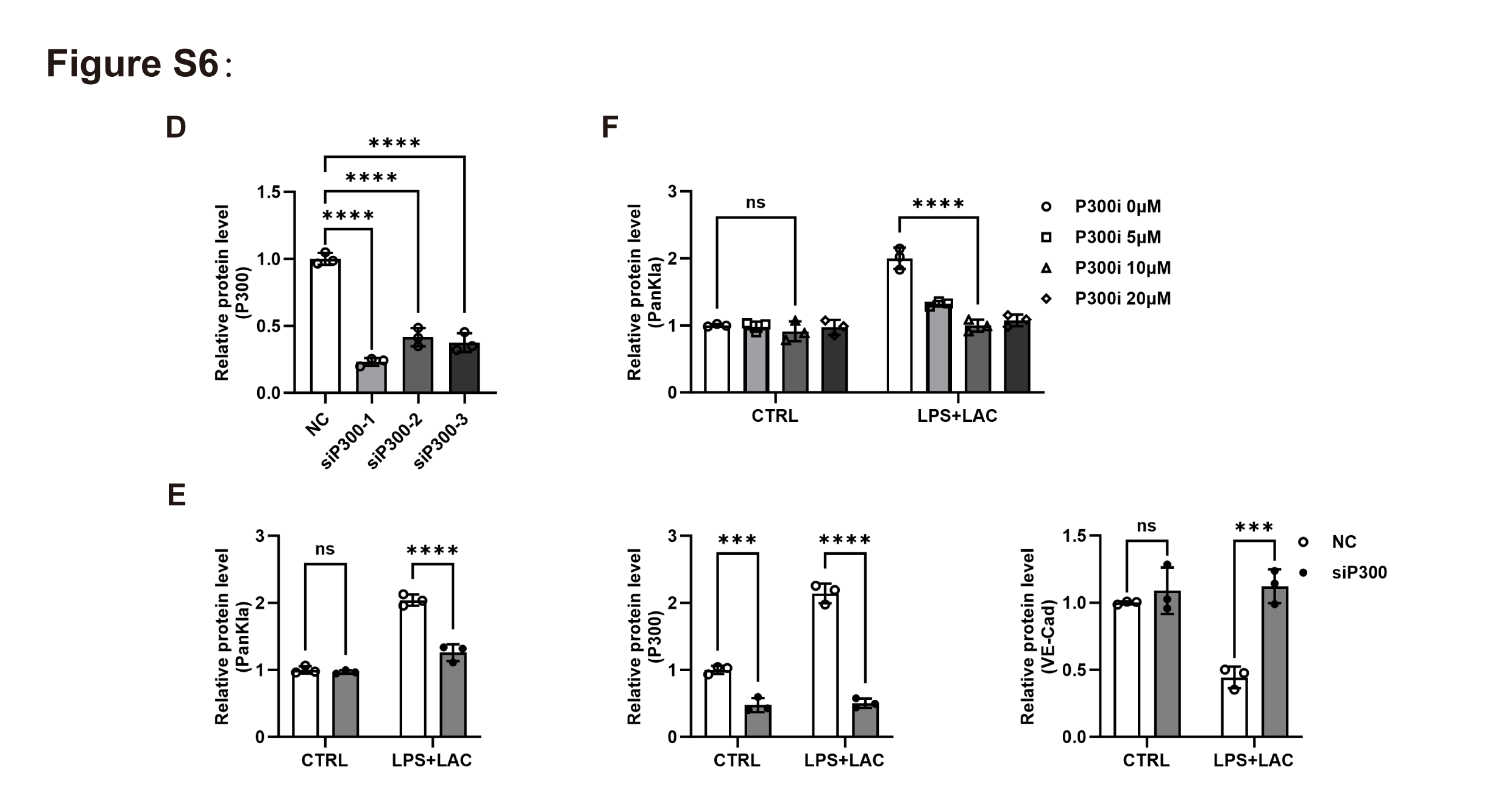


**Figure S6. Quantification of western blots in Figure S6.** (D) Relative protein level of P300 in Figure S6D (n=3 per group). (E) Relative protein level of PanKla、P300 and VE-Cadherin in Figure S6E (n=3 per group). (F) Relative protein level of PanKla in Figure S6F (n=3 per group). Data are presented as mean ± SD. ns, not significant, ***P < 0.001, ****P < 0.0001.


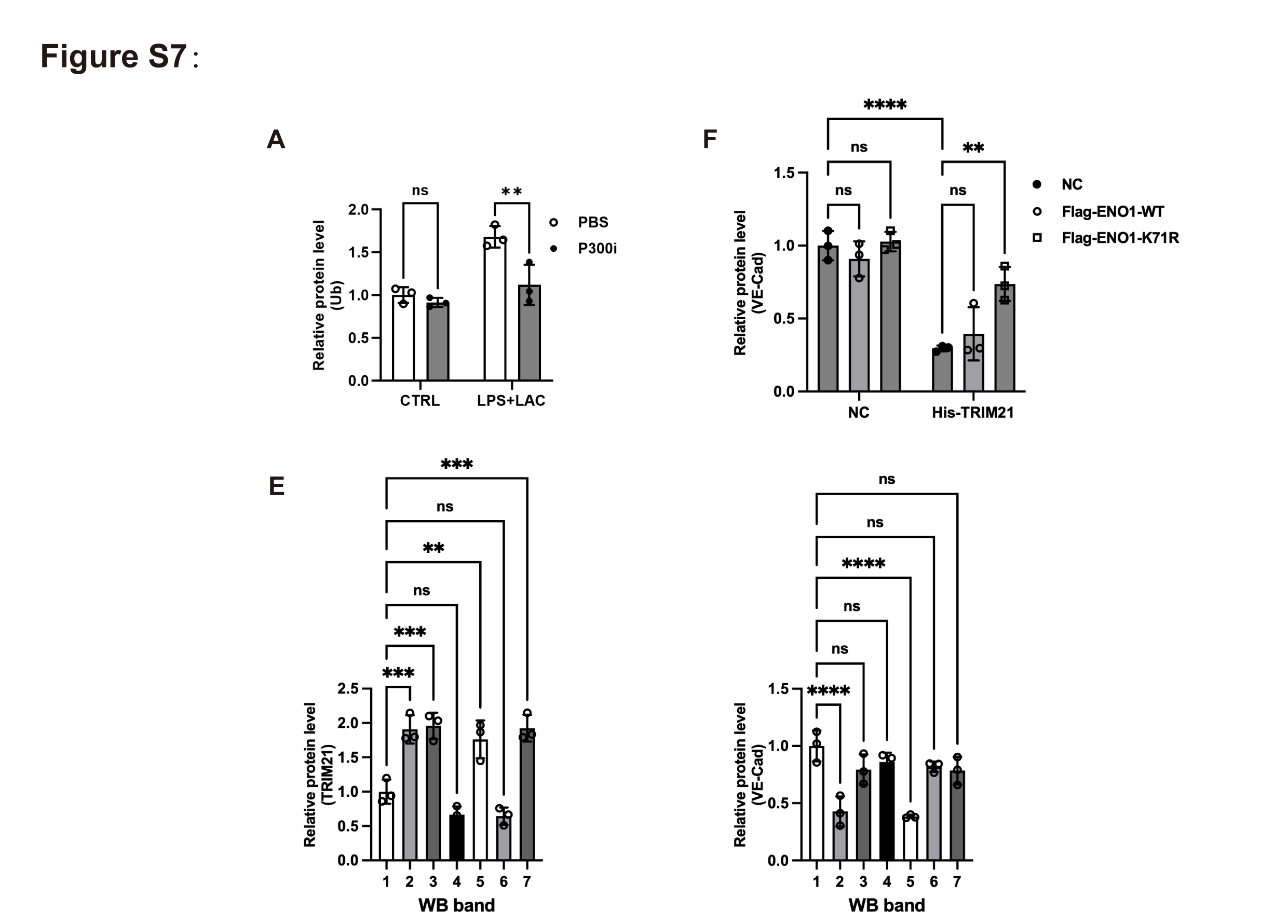


**Figure S7. Quantification of western blots in Figure S7.** (A) Relative protein level of Ub in Figure S7A (n=3 per group). (E) Relative protein level of TRIM21 and VE-Cadherin in Figure S7E (n=3 per group). (F) Relative protein level of VE-Cadherin in Figure S7F (n=3 per group). Data are presented as mean ± SD. ns, not significant, **P < 0.01, ***P < 0.001, ****P < 0.0001.


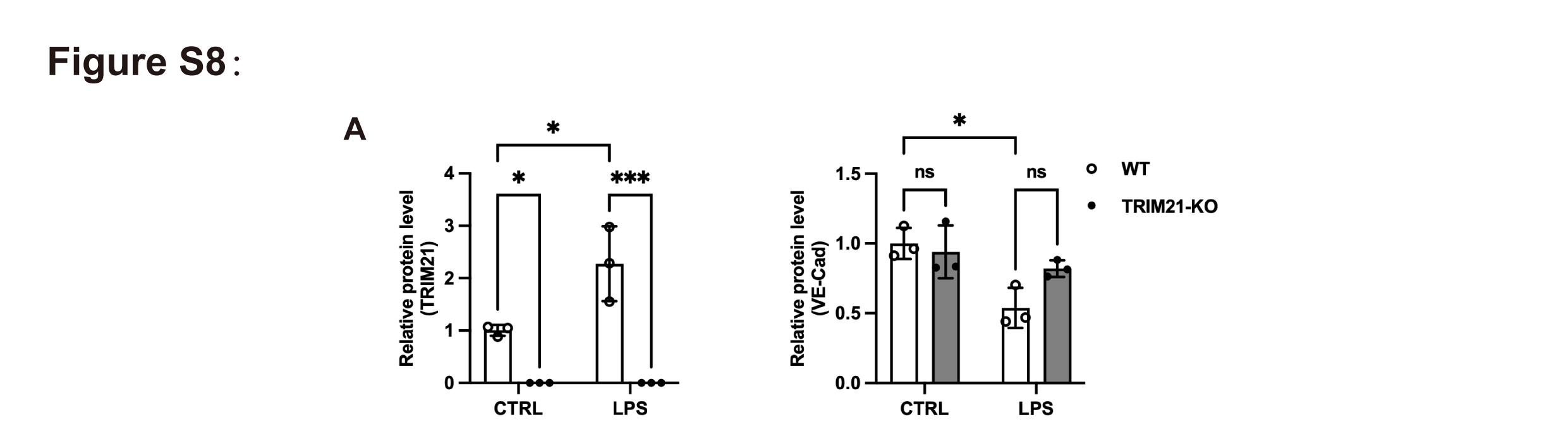


**Figure S8. Quantification of western blots in Figure S8.** (A) Relative protein level of TRIM21 and VE-Cadherin in Figure S8A (n=3 per group). Data are presented as mean ± SD. ns, not significant, *P < 0.05, ***P < 0.001.


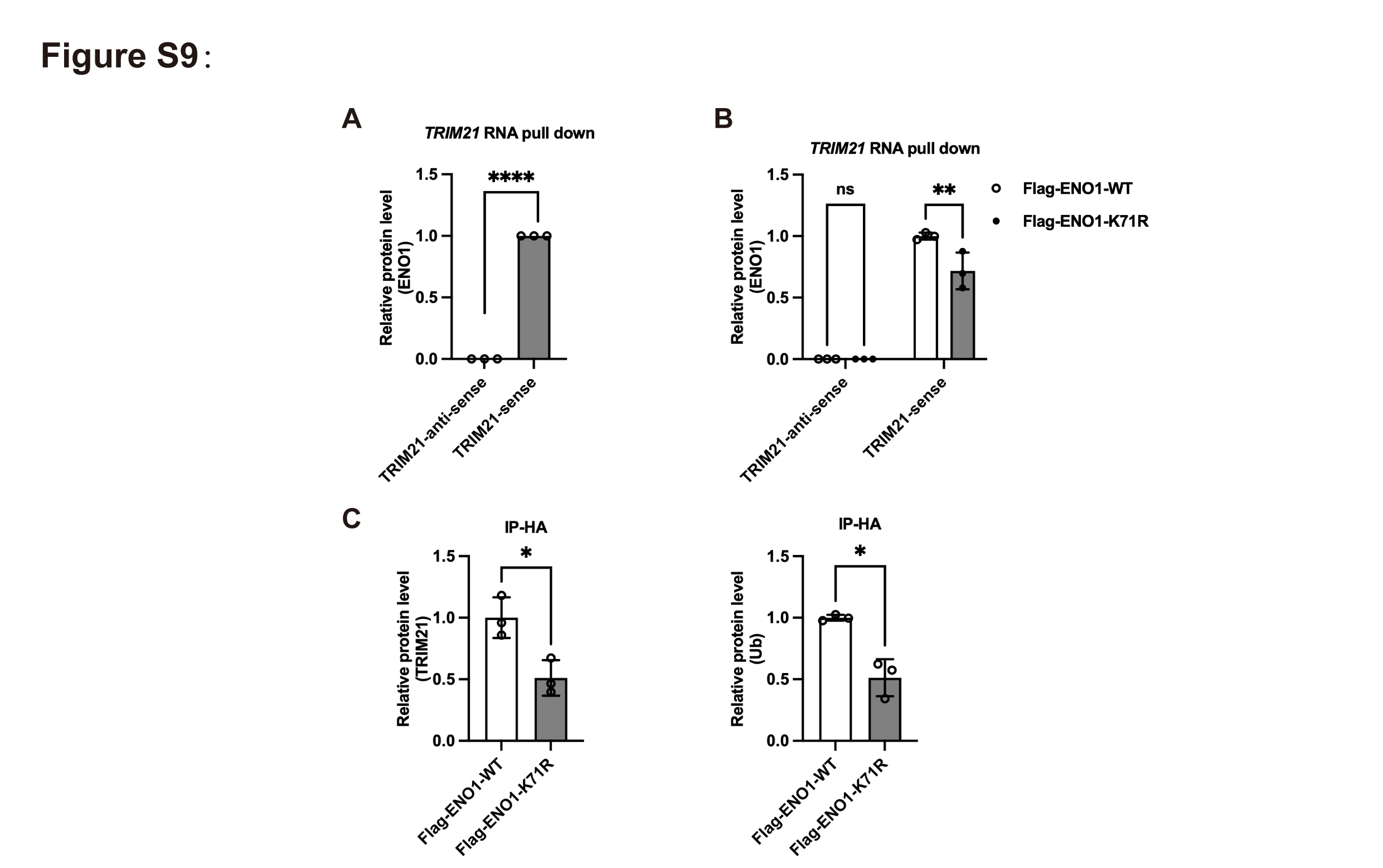


**Figure S9. Quantification of western blots in Figure S9.** (A) Relative protein level of ENO1 in Figure S9A (n=3 per group). (B) Relative protein level of ENO1 in Figure S9B (n=3 per group). (C) Relative protein level of TRIM21 and Ub in IP group in Figure S9C (n=3 per group). Data are presented as mean ± SD. ns, not significant, *P < 0.05, **P < 0.01, ***P < 0.001.


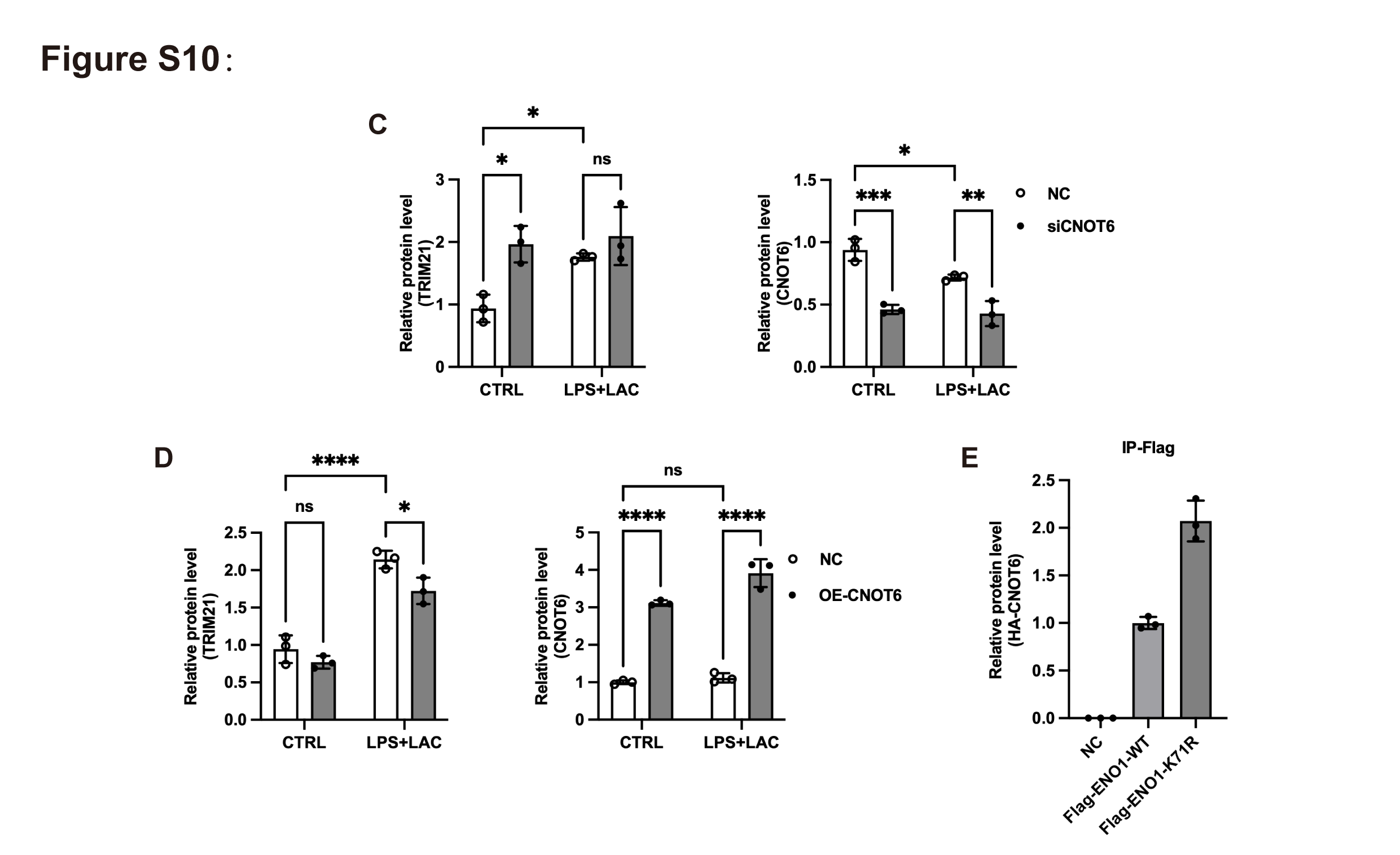


**Figure S10. Quantification of western blots in Figure S10.** (C) Relative protein level of TRIM21 and CNOT6 in Figure S10C (n=3 per group). (D) Relative protein level of TRIM21 and CNOT6 in Figure S10D (n=3 per group). (E) Relative protein level of HA-CNOT6 in IP group in Figure S10E (n=3 per group). Data are presented as the mean ± SD. *P < 0.05, ****P < 0.0001.


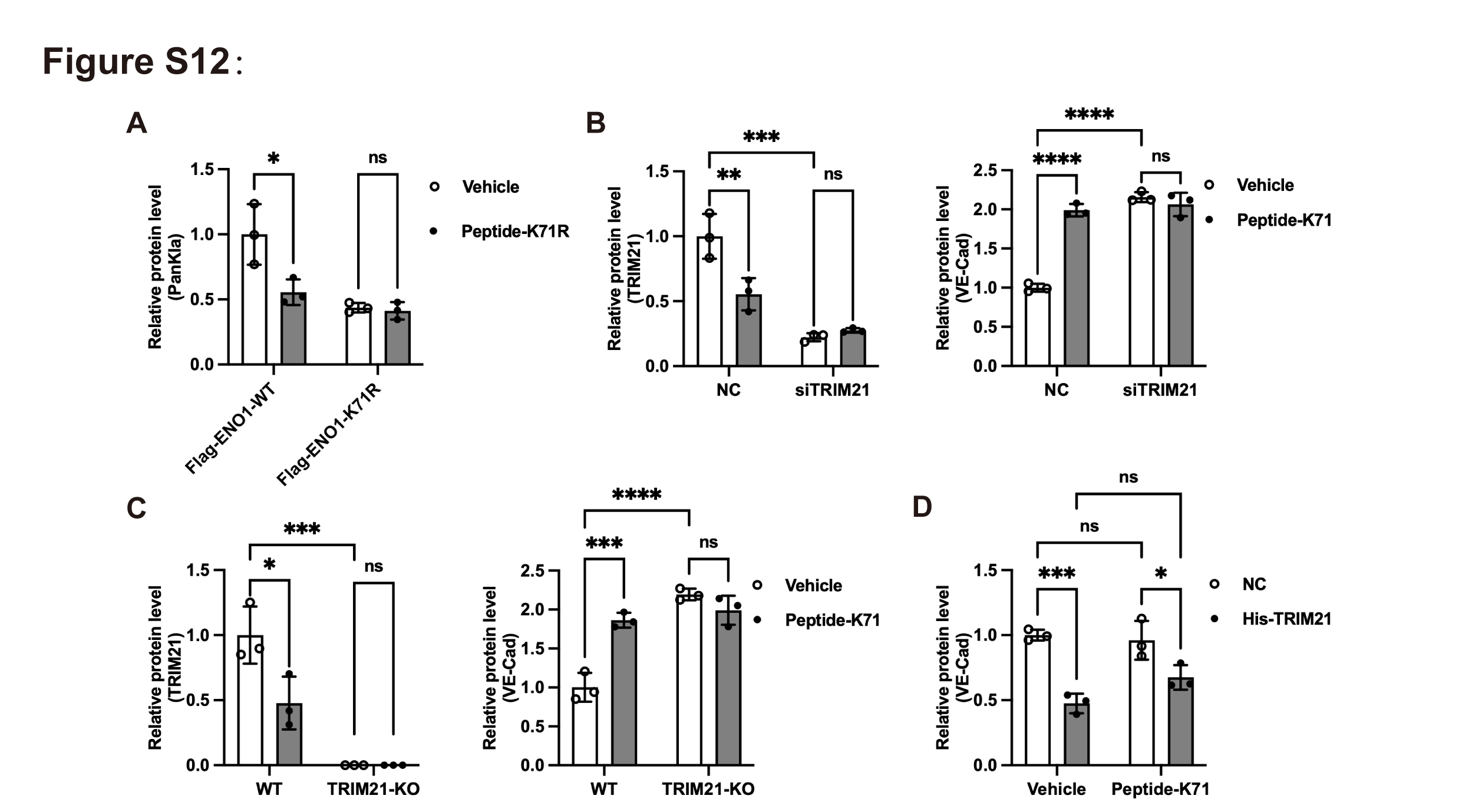


**Figure S12. Quantification of western blots in Figure S12.** (A) Relative protein level of PanKla in Figure S12A (n=3 per group). (B) Relative protein level of TRIM21 and VE-Cadherin in Figure S12B (n=3 per group). (C) Relative protein level of TRIM21 and VE-Cadherin in Figure S12C (n=3 per group). (D) Relative protein level of VE-Cadherin in Figure S12D (n=3 per group). Data are presented as mean ± SD. ns, not significant, *P < 0.05, ***P < 0.001, ****P < 0.0001.


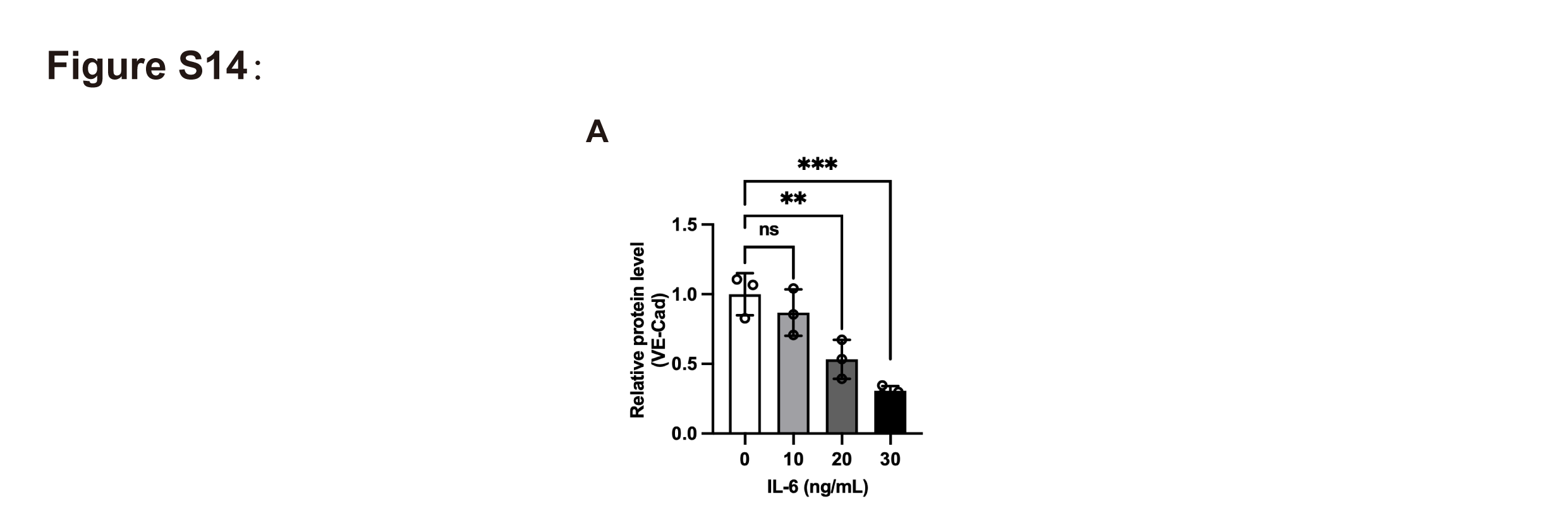


**Figure S14. Quantification of western blots in Figure S14.** (A) Relative protein level of VE-Cadherin in Figure S14A (n=3 per group). Data are presented as the mean ± SD. ns, not significant, **P < 0.01, ***P < 0.001.

Singer, M., et al. (2016). "The Third International Consensus Definitions for Sepsis and Septic Shock (Sepsis-3)." Jama 315(8): 801-810.

IMPORTANCE: Definitions of sepsis and septic shock were last revised in 2001. Considerable advances have since been made into the pathobiology (changes in organ function, morphology, cell biology, biochemistry, immunology, and circulation), management, and epidemiology of sepsis, suggesting the need for reexamination. OBJECTIVE: To evaluate and, as needed, update definitions for sepsis and septic shock. PROCESS: A task force (n = 19) with expertise in sepsis pathobiology, clinical trials, and epidemiology was convened by the Society of Critical Care Medicine and the European Society of Intensive Care Medicine. Definitions and clinical criteria were generated through meetings, Delphi processes, analysis of electronic health record databases, and voting, followed by circulation to international professional societies, requesting peer review and endorsement (by 31 societies listed in the Acknowledgment). KEY FINDINGS FROM EVIDENCE SYNTHESIS: Limitations of previous definitions included an excessive focus on inflammation, the misleading model that sepsis follows a continuum through severe sepsis to shock, and inadequate specificity and sensitivity of the systemic inflammatory response syndrome (SIRS) criteria. Multiple definitions and terminologies are currently in use for sepsis, septic shock, and organ dysfunction, leading to discrepancies in reported incidence and observed mortality. The task force concluded the term severe sepsis was redundant. RECOMMENDATIONS: Sepsis should be defined as life-threatening organ dysfunction caused by a dysregulated host response to infection. For clinical operationalization, organ dysfunction can be represented by an increase in the Sequential [Sepsis-related] Organ Failure Assessment (SOFA) score of 2 points or more, which is associated with an in-hospital mortality greater than 10%. Septic shock should be defined as a subset of sepsis in which particularly profound circulatory, cellular, and metabolic abnormalities are associated with a greater risk of mortality than with sepsis alone. Patients with septic shock can be clinically identified by a vasopressor requirement to maintain a mean arterial pressure of 65 mm Hg or greater and serum lactate level greater than 2 mmol/L (>18 mg/dL) in the absence of hypovolemia. This combination is associated with hospital mortality rates greater than 40%. In out-of-hospital, emergency department, or general hospital ward settings, adult patients with suspected infection can be rapidly identified as being more likely to have poor outcomes typical of sepsis if they have at least 2 of the following clinical criteria that together constitute a new bedside clinical score termed quickSOFA (qSOFA): respiratory rate of 22/min or greater, altered mentation, or systolic blood pressure of 100 mm Hg or less. CONCLUSIONS AND RELEVANCE: These updated definitions and clinical criteria should replace previous definitions, offer greater consistency for epidemiologic studies and clinical trials, and facilitate earlier recognition and more timely management of patients with sepsis or at risk of developing sepsis.

1. Singer, M., et al., The Third International Consensus Definitions for Sepsis and Septic Shock (Sepsis-3). Jama, 2016. 315(8): p. 801-10.

1. Singer, M., et al., The Third International Consensus Definitions for Sepsis and Septic Shock (Sepsis-3). Jama, 2016. 315(8): p. 801-10.

2. Certo, M., et al., Lactate modulation of immune responses in inflammatory versus tumour microenvironments. Nat Rev Immunol, 2021. 21(3): p. 151-161.

1. Singer, M., et al., The Third International Consensus Definitions for Sepsis and Septic Shock (Sepsis-3). Jama, 2016. 315(8): p. 801-10.

2. Certo, M., et al., Lactate modulation of immune responses in inflammatory versus tumour microenvironments. Nat Rev Immunol, 2021. 21(3): p. 151-161.

3. Cecconi, M., et al., Sepsis and septic shock. Lancet, 2018. 392(10141): p. 75-87.

1. Singer, M., et al., The Third International Consensus Definitions for Sepsis and Septic Shock (Sepsis-3). Jama, 2016. 315(8): p. 801-10.

2. Certo, M., et al., Lactate modulation of immune responses in inflammatory versus tumour microenvironments. Nat Rev Immunol, 2021. 21(3): p. 151-161.

4. Chen, Y., et al., Metabolic regulation of homologous recombination repair by MRE11 lactylation. Cell, 2024. 187(2): p. 294-311.e21.
